# **The SURVIVE study (NCT05658172): Bringing breast cancer aftercare to the 21**^**st**^**century:** Study protocol of a Phase III clinical trial comparing liquid biopsy guided vs. Standard of care surveillance for intermediate to high-risk breast cancer survivors

**DOI:** 10.1371/journal.pone.0331203

**Published:** 2025-09-09

**Authors:** Tatjana Braun, Sophia Huesmann, Kerstin Pfister, Thomas W. P. Friedl, Andreas Hartkopf, Klaus Pantel, Forca Mehmeti, Franziska Mergel, Henning Schäffler, Sabine Heublein, Lisa Wiesmüller, Brigitte Rack, Peter A. Fasching, Wolfgang Janni, Angelina Fink

**Affiliations:** 1 Department of Gynecology and Obstetrics, University Hospital Ulm, Ulm, Germany; 2 Institute of Women’s Health, University Hospital Tübingen, Tübingen, Germany; 3 Center for Experimental Medicine, Institute of Tumor Biology, University Hospital Hamburg-Eppendorf, Hamburg, Germany; 4 Department of Gynecology and Obstetrics, SLK-Kliniken Heilbronn, Heilbronn, Germany; 5 Department of Gynecology and Obstetrics, University Hospital Erlangen, Erlangen, Germany; Nelson Mandela African Institute of Science and Technology, TANZANIA, UNITED REPUBLIC OF

## Abstract

**Background:**

Current aftercare in breast cancer survivors aims to detect local recurrences or contralateral disease, while the detection of distant metastases has not been a central focus due to a lack of evidence supporting an effect on overall survival. However, the data underpinning these guidelines are mainly from trials of the 1980s/1990s and have not been updated to reflect the significant advancements in diagnostic and therapeutic options that have emerged over the past 40 years. In this trial, the aim is to test whether a liquid biopsy-based detection of (oligo-) metastatic disease at an early pre-symptomatic stage followed by timely treatment can impact overall survival compared to current standard aftercare.

**Methods:**

In this partially double-blinded superiority study, intensified liquid biopsy-guided surveillance will be assessed versus standard surveillance in medium-to-high-risk early breast cancer patients. Intensive surveillance comprises 3-monthly tests of circulating free tumor DNA (ctDNA), circulating tumor cells (CTC) and serum tumor markers CEA, CA 27.29 and CA125. Upon positivity of biomarker and/or symptoms, staging examinations are initiated. In total, 3500 patients will be randomized in a 1:1 ratio after completion of primary antineoplastic therapy. Co-primary endpoints are overall survival (OS) and the overall lead time effect. The trial will be accompanied by an extensive translational research program.

**Discussion:**

A risk-based aftercare and regular screening for asymptomatic metastatic disease with molecular markers in the absence of any radiological findings can potentially revolutionize current follow-up care of breast cancer survivors and enable potential treatment even before patients suffer from symptomatic, incurable disease.

## Introduction

Breast cancer is second place in the world’s global cancer incidence with nearly 2.3 million people newly diagnosed in 2022 [1] with a mortality of over 650 000 in the same year. Consequently, there are millions of breast cancer survivors who require follow-up care after completing their primary treatment. Current aftercare guidelines, e.g., from the American Society of Clinical Oncology [2] or the European Society of Medical Oncology [3], are focusing on detecting local recurrences or contralateral tumors, management of therapy-related toxicities and adherence as well as enhancement of a healthy lifestyle and psychological well-being. Therefore, regular imaging is only advised in the form of mammography and ultrasound of the breast. Only in case of symptoms that could be a manifestation of distant metastases such as dyspnea or bone pain, further laboratory tests or imaging via a CT or bone scan should be performed. A screening with tumor markers or a routine performance of staging examinations in asymptomatic patients is not advised because of data suggesting no benefits of these tests. These recommendations are based on data originating from two trials conducted in the 1980s and 1990s, which compared intensified breast cancer aftercare incorporating additional imaging with the standard-of-care (SoC) follow-up. Even though the results showed that distant metastases could be detected earlier in the intensive surveillance group (112 cases vs 71 cases in the SoC group) [4], no difference regarding overall survival (OS) could be observed [4,5]. The most recent meta-analysis on this topic from 2005 could also not see a difference regarding OS [6].

However, significant advancements in diagnostic and therapeutic approaches to breast cancer have emerged over the past decades. In the late 1990s, the aromatase inhibitors anastrozole and letrozole have been approved first in the metastatic setting, then in the early 2000s in the adjuvant setting. Simultaneously, trastuzumab gained approval offering targeted therapy to HER2 positive breast cancer patients. In the following years, drugs like CDK 4/6 inhibitors and checkpoint inhibitors substantially changed the therapeutic landscape, leading to an improved OS not only in the metastatic but also in the adjuvant setting [7,8]. These new drugs can prevent local recurrences as well as metastatic diseases, e.g., as shown in the MonarchE trial [9]. Moreover, another novel approach to individualized therapy in breast cancer patients using antibody-drug conjugates such as Trastuzumab Deruxtecan and Sacituzumab Govitecan has proven to significantly improve progression-free survival (PFS) and OS not only in patients with HER2 (Human Epidermal Growth Factor Receptor 2) positive but also with triple negative advanced breast cancer [10].

Regarding diagnostics, circulating tumor cells (CTCs) have been researched and proved to be a prognostic factor regarding breast cancer. Janni et al. could show that detection of CTCs – which was the case in 20.2% of patients with breast cancer stages I-III – was associated with poor prognosis at the time of primary diagnosis [11]. Risk factors for recurrence like larger tumor size, higher histological grading and involvement of several lymph nodes during primary disease were significantly correlated with the presence of at least one CTC. Independently of these risk factors, the presence of at least one CTC was still a negative prognostic factor regarding disease-free survival [HR, 1.82; 95% confidence interval (CI), 1.47-2.26], distant disease-free survival (HR, 1.89; 95% CI, 1.49-2.40), breast cancer-specific survival (HR, 2.04; 95% CI, 1.52-2.75), and OS (HR, 1.97; 95% CI, 1.51-2.59) [11]. But not only at time of primary diagnosis CTCs have proven to be of prognostic relevance. Rack et al. could show that detection of CTCs after chemotherapy was an independent negative prognostic marker regarding disease-free survival (DFS), distant DFS, breast cancer-specific survival and overall survival [12]. Trapp et al. could demonstrate that even 2 years after completion of chemotherapy CTCs still were of prognostic relevance and were associated with decreased OS and DFS [13].

Tumor markers in breast cancer are currently only used in the clinical routine to monitor disease in the metastatic setting. However, soluble MUC1 is a known biomarker for prediction of prognosis and treatment efficacy as it was shown that MUC1 levels are associated with tumor burden. The tumor marker cancer antigen (CA) 27.29 is one way to measure MUC1. In patients that were enrolled in the SUCCESS-A trial, it was shown that CA27.29 before chemotherapy was associated with DFS in the sense of higher CA27.29 levels showed poorer DFS [14].

Circulating tumor DNA (ctDNA) has also shown to be a prognostic marker in early breast cancer, with a decrease in invasive disease-free survival and overall survival if ctDNA is detected [15]. There are two approaches to detect ctDNA in patients’ peripheral blood specimens. ctDNA can be detected either with a tumor informed approach, creating a patient specific primer panel using somatic variants identified through exome sequencing of primary tumor tissue, or with a non-tumor informed approach using a predefined panel of mutations. Using a tumor-informed ctDNA test, Lipsyc-Sharf et al. could show in patients with hormone receptor positive and HER2 negative breast cancer patients five years after completion of primary therapy, that 10% (n = 8) were ctDNA positive [16]. Out of these patients, six developed metastatic disease with a median lead time from ctDNA detection to via imaging confirmed metastatic disease of about 12 months [16]. Other publications showed similar results [17]. Similarly, a non-tumor informed ctDNA test showed high sensitivity and specificity in the detection of distant metastases with a lead time of up to two years in breast cancer patients that have completed primary therapy up to four years earlier [18].

Thus, these markers of molecular residual disease might be used as an indicator of upcoming distant metastases, thereby guiding the use of imaging to detect metastatic disease at an early, pre-symptomatic stage. Initiation of therapy in this earlier stage may represent a chance to prevent stressful symptoms, prolong overall survival, or even potentially cure (pre- or oligo-) metastatic disease.

## Materials and methods

### Aim, study design, endpoints and sample size calculation

The aim of the SURVIVE study is to evaluate whether an updated and individualized breast cancer surveillance in medium and high-risk patients can improve the prognosis of these patients.

**Fig 1:**
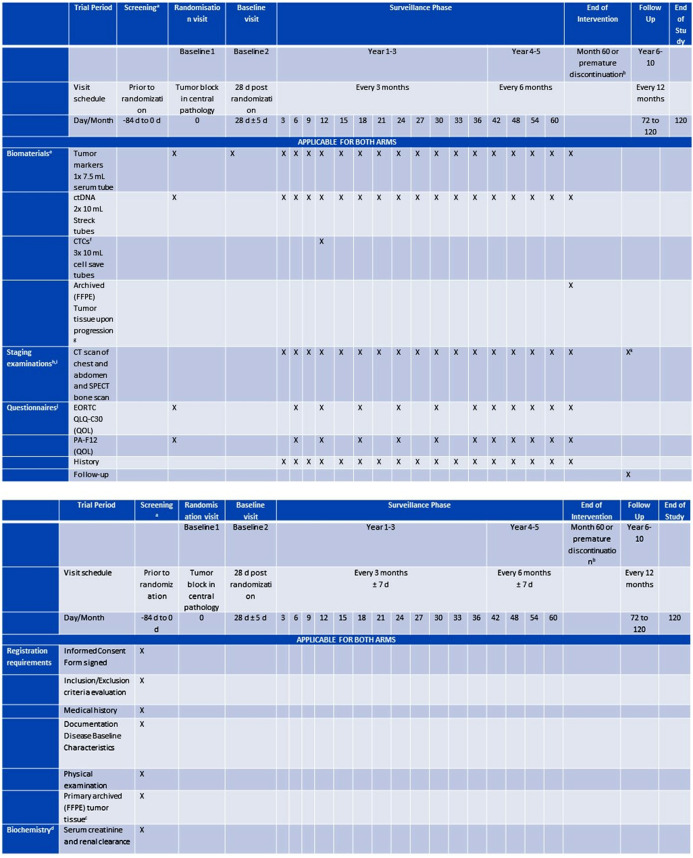
SPIRIT schedule of enrollment, interventions, and assessments. **a:** Results of screening tests or examinations performed as standard of care prior to obtaining informed consent but within the timelines may be used rather than repeating required test; **b:** Premature study intervention discontinuation due to either of the following: **1.** via imaging verified recurrence, **2.** Pregnancy, **3.** safety or medical reasons, **4.** patients’ withdrawal of consent; **c:** Primary tumor tissue according to the requirements stated in the study protocol section 11.2. Important note: in case of bilateral breast cancer, tissue from both sides is needed. For multifocal breast cancer: tissue sample of one focus is sufficient, if the tumor biology among the foci does not differ. However, if the tumor biology among the foci differs, tissue of the therapy-defining focus, according to the investigator, should be used; **d:**Results of serum creatinine and renal clearance performed within 84 days prior to obtaining informed consent may be used rather than repeating required test; **e:** Materials for blood collection and transportation devices will be provided by the Central Study Center at Dep. Ob/Gyn University Hospital Ulm; **f:** Mandatory at Month 12, additionally required at visit after first verified tumor marker increase, whenever that occurs; **g:** Highly endorsed in case of detection of recurrence/metastatic disease; **h:** Referral to radiologist from investigator or aftercare specialist (gynecologist/family physician/oncologist) – Can be triggered by either of the following: **1.** clinical indication (both arms), **2.** increase in tumor markers (only intensive surveillance arm), **3.** if presence of ctDNA was detected (only intensive surveillance arm); **i:** If CT and SPECT not possible due to, e.g., allergy to the contrast agent: PET scan or whole-body MRI, Complementary imaging tests if indicated; **j:** Available on paper, handed out at study visits; **k:** Staging examinations or Breast Imaging obtained during the standard aftercare visits should be documented in the eCRF (e.g., Bilateral mammography and supplementary bilateral breast ultrasound with axillary lymph nodes (where applicable) and/or MRI of the breasts.

**Fig 2:**
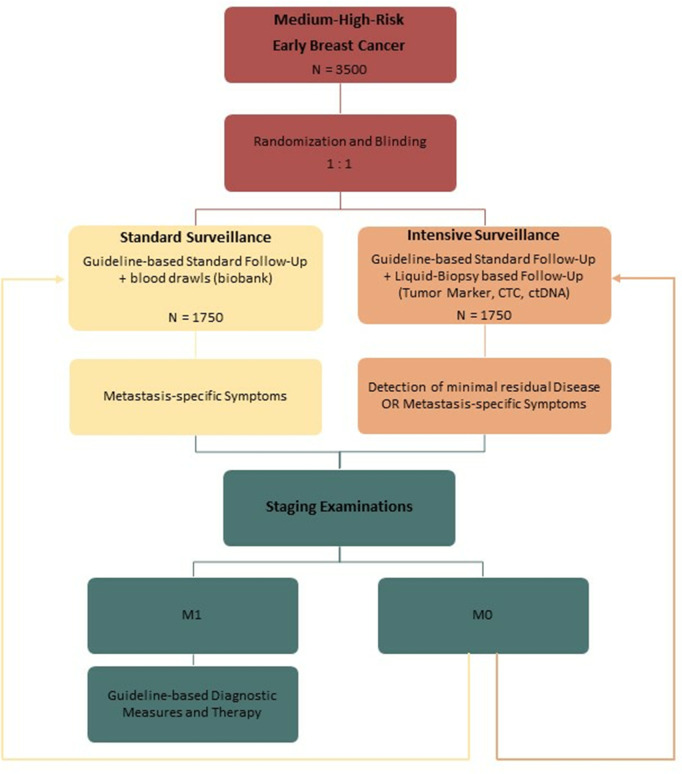
Study design. ctDNA = Circulating free tumor DNA, CTC = circulating tumor cells, M0 = no evidence of distant metastases, M1 = evidence of distant metastasis.

**Fig 3:**
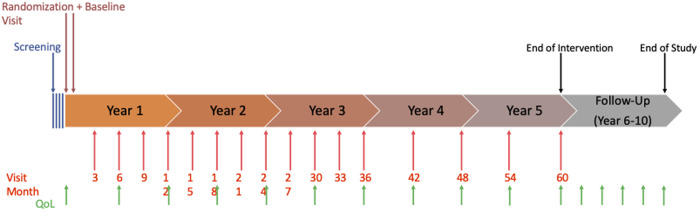
Timeline of the SURVIVE study. QoL = Quality of life.

After enrollment, all participants will follow guideline based standard follow-up care in addition to blood sample collection every 3 months for the first 3 years and every 6 months for the consecutive 2 years. In case of detection of distant metastases, contralateral or local recurrence of disease, the patients will receive guideline based diagnostic measures and therapy (Fig 1, 2). Quality of Life (QoL) will be monitored every 6 months by standardized questionnaires (QLQ-C30 and PA-F12) (Fig 1, 3). Randomization will be in a 1:1 ratio to the intensive versus standard surveillance group and is blinded for patients and participating study centers (Fig 2). In the intensive surveillance group, the blood will be tested for tumor markers CA27.29 (comparable to CA15−3), CA125, carcinoembryonic antigen (CEA), CTCs and ctDNA (Fig 1, 2). For ctDNA testing, a tumor informed approach will be used, using primary tumor tissue to create an individualized primer set for each patient. Regarding the use of the tumor marker CA 27.29 instead of CA 15.3, it has been shown that they are usually concordant, but it is not advised to use them interchangeably [19]. Therefore, one could argue if the results of this trial are transferable to CA 15.3. However, the immunoassays for CA15.3 and CA27.29 target the epitopes on the same MUC1 – which is associated with higher tumor burden – and are considered identical [20,21]. In the SOC group, the blood samples will be stored in a biobank for translational research. If there is a positive biomarker result in the Intensive Surveillance group, the treatment centers and therefore the patients will be notified of the result (and consequently unblinded), and imaging in terms of CT of chest and abdomen and bone scan is advised. If distant metastases are detected, patients should receive therapy in accordance with national guidelines. If there is no evidence of disease, the patient will continue to receive blood testing in the scheduled intervals.

Co-primary endpoints will be 5-year OS rate and the overall lead time effect. The latter is defined as the median time from molecular to via imaging verified distant recurrence Lead Time (which can be calculated only for patients in the liquid-biopsy guided arm) plus the difference in time to distant recurrence between the two study groups.

Secondary endpoints are:

- Invasive disease-free survival (IDFS)
- Distant disease-free survival (DDFS)
- Distant recurrence-free survival (DRFS)
- Breast cancer specific survival (BCSS)
- Invasive breast cancer free survival (IBCFS)
- 10-year OS rate
- Molecular to via Imaging verified Distant Recurrence Lead Time in the Interventional arm
- Quality of life (QoL) with questionnaires: EORTC QLQ-C30 and PA-F12
- Liquid biopsy sensitivity (CA27.29, CEA, CA125, CTC and ctDNA)
- Liquid biopsy specificity (CA27.29, CEA, CA125, CTC and ctDNA)
- Liquid Biopsy False-Positive Rate (CA27.29, CEA, CA125, CTC and ctDNA)
- Liquid Biopsy False-Negative Rate (CA27.29, CEA, CA125, CTC and ctDNA)
- Rate of liquid biopsy positivity (CA27.29, CEA, CA125, CTC and ctDNA)

The study is designed as a two-arm parallel, (partially) double-blinded randomized superiority study. According to the sample size calculation, approximately 3500 randomized patients (1750 in both arms) will be required to achieve 80% power at a 2-sided significance level of 5% to detect a 2.35% improvement in 5-year OS rate from 92.65% in the standard surveillance arm to 95.0% in the tumor marker, ctDNA and CTC guided surveillance arm (equivalent to a hazard ratio of 0.672) using a log-rank test. The reference value of 92.65% 5-year OS rate is based on the results from the randomized phase III Success A study that reported 5-year OS rates for 3754 intermediate-to-high-risk early breast cancer patients receiving standard chemotherapy regimen [22]. Furthermore, this sample size calculation is based on the assumptions of a uniform accrual pattern (accrual time 2 years), a dropout rate of 15% in both arms and a total study duration of 10 years.

### Study population

Participants must have histologically confirmed, intermediate to high-risk breast cancer without any evidence of distant disease. The primary antineoplastic therapy should have been completed (adjuvant chemotherapy, surgery or radiotherapy, whichever occurs last) no more than 24 months prior to study enrollment in triple-negative or HER2 positive breast cancer (Table 1). For hormone receptor positive disease, enrollment can take place up to 60 months after the end of primary anti-tumor therapy (Table 1). Enrollment of patients during any kind of adjuvant therapy except chemotherapy (e.g., but not limited to endocrine therapy, antibody therapy, CDK4/6-inhibitors, PARP inhibitors, PI3K inhibitors, antibody-drug conjugates and other novel agents) is allowed. Sufficient amount of primary tumor tissue must be available to be able to perform the tumor-informed ctDNA test (Table 1).

**Table 1:** Main inclusion and exclusion criteria.

Inclusion Criteria	Exclusion Criteria
Written informed consent for all study procedures according to local regulatory requirements prior to beginning specific protocol procedures.	Patients with a history of any secondary primary malignancy are ineligible with the following exceptions: - in situ carcinoma of the cervix or - adequately treated basal cell carcinoma of the skin or - ipsi- or contralateral non-invasive carcinoma of the breast (DCIS).
Unilateral or bilateral primary invasive carcinoma of the breast, confirmed histologically.	Patients in pregnancy or breastfeeding.
Patients with intermediate- to high-risk early breast cancer defined as either - an indication for (neo-)adjuvant chemotherapy (regardless whether performed or not), and/or - Large tumor (> 50 mm), and/or - Positive lymph nodes (> pN1mi), and/or - High grade (G3).	History of significant neurological or psychiatric disorders including psychotic disorders, dementia or seizures that would prohibit the understanding and giving of informed consent
A complete resection of the primary tumor, with resection margins free of invasive carcinoma.	Renal insufficiency with GFR < 30 mL/min.
Completion of primary anti-tumor therapy (adjuvant chemotherapy, surgery or radiotherapy, whichever occurs last) no more than 24 months previously. Enrollment of patients during any kind of adjuvant therapy except chemotherapy (e.g., but not limited to endocrine therapy, antibody therapy, CDK4/6-inhibitors, PARP inhibitors, PI3K inhibitors, antibody-drug conjugates and other novel agents) is allowed. Patients with Luminal A/B breast cancer (ER/PGR positive, HER2 negative/low) may be enrolled up to 60 months after completion of primary anti-tumor therapy.	Previous or concomitant cytotoxic or other systemic antineoplastic treatment that is not used for treating the primary breast cancer.
Availability of primary tumor tissue from core biopsy or surgical removed tissue. Important note: Tumor tissue of both sides will be needed in case of bilateral breast cancer.	
No current clinical evidence for distant metastases.	
Females or males ≥ 18 years and ≤ 75 years of age.	
Performance status ≤ 1, Eastern Cooperative Oncology Group (ECOG) scale.	
Patient must be willing and able to comply with scheduled visits, treatment plans, laboratory tests, and other study procedures.	

### Study interventions and randomization

In total, 3500 patients shall be enrolled in the SURVIVE trial and will be randomized in a 1:1 ratio in either the intensive surveillance or the standard surveillance group. Stratification factors will be hormone receptor status (negative vs positive), HER2 status (negative vs positive) and histological lymph node status at surgery ((y)pN0 vs (y)pN+).

Peripheral blood samples will be drawn from patients in both groups every 3 months for the first 3 years after study inclusion, thereafter every 6 months for another 2 years. In the Standard of care group, the blood will be stored in a biobank for further translational studies and retrospective analyses. In the Intensive Surveillance group, the blood will be analyzed regarding tumor markers (CA27.29, CEA and CA125) and ctDNA levels. CTCs analysis will be performed in every patient of the Intensive Surveillance group one year after enrollment and will furthermore be triggered after the first detection of abnormal tumor marker levels. Based on evidence for associations with tumor load, an abnormal increase in serum tumor marker levels as well as detection of ctDNA or CTCs will initiate further staging examinations for patients in the Intensive Surveillance group.

An abnormal increase in serum tumor markers is defined as a delta in increase from baseline (CA27.29 + 75% and/or CEA + 100% and/or CA125 + 150%), because current available data suggest that using kinetics improve sensitivity and specificity for the detection of distant metastases compared to static cut-offs [23–25]. One of the hypothesized reasons is heterogeneity of serum levels on an inter- and intraindividual level due to several possible interferences such as renal function, current health status (in particular infections) or comorbidities. In contrast to serum tumor markers, where a defined increase compared to individual baseline values is necessary to trigger imaging, a single positive CTC or ctDNA result is sufficient to trigger the performance of staging examinations.

Given the possibility of several subsequent positive liquid biopsy results without detection of metastases in the corresponding imaging, certain rules have been established to restrict the radiation exposure for patients. Generally, and used independently from the liquid biopsy marker, the minimum interval between two staging examinations should be at least 3 months for CT scans and at least 6 months for bone scans. In addition, there are the following biomarker-specific algorithms: If there is a serum tumor marker elevation at subsequent time points without any evidence of metastatic disease as confirmed by imaging, a maximum of three consecutive negative staging examinations will be performed before a further increase in tumor marker levels will be required (according to the deltas stated above) to trigger another imaging. If a patient is CTC-positive and the performed corresponding imaging was negative, the regular standard liquid biopsy testing will commence without further CTC testing. In case of ctDNA positivity and negative imaging the patients will continue liquid biopsy-based surveillance. If the ctDNA persists a minimum interval of three months between two CT-scans is mandatory.

If clinical symptoms arise that could indicate distant metastases, patients in both groups should undergo further staging examinations in accordance with current national guidelines. If distant metastases are verified by imaging, patients must be treated according to the current standard of care, thereby ending the intervention phase of the SURVIVE study.

QoL is assessed every six months by using the two questionnaires EORTC QLQ C30, which is well established regarding general quality of life, and the PA-F12, which focuses on fear of disease progression.

A follow-up of five years with yearly checks on patients’ well-being, QoL and status of recurrence will take place after the end of the 5-year intervention phase.

### Statistical analysis and data management plan

The primary endpoint overall survival will be analyzed based on the intention-to-treat (ITT) set, estimated by the Kaplan Meier product limit method, and 5-year OS rates, 95% confidence intervals and survival plots will be provided. Overall survival will be compared between patients in the two randomization arms using the log-rank test, and univariable cox regression as well as additional multivariable analyses adjusted for other factors will be performed using suitable regression models (cox proportional hazard regression model). Factors to be adjusted for will comprise the stratification factors hormone receptor status, HER2 status and lymph node status at surgery as well as other potentially prognostic clinic-pathological parameters such as age, body mass index, tumor size, tumor grade and tumor histology. The final variable list for the multivariable analysis will be set forth in a statistical analysis plan (SAP), which will be finalized prior to data base lock. Hazard ratios and the corresponding 95% confidence intervals will be reported as obtained by both univariable and adjusted multivariable analyses. The co-primary endpoint Overall Lead Time Effect will be calculated as described above. As it is a purely descriptive composite measure consisting of two unrelated median values calculated for different patient cohorts, no confidence intervals will be provided. Given that the co-primary endpoint Overall Lead Time Effect is purely descriptive and does not involve a statistical comparison between the two randomization arms, there is no need for adjusting the significance level for multiplicity for the primary endpoint analyses.

A three-step futility analysis to assess ctDNA positivity rate and molecular as well as overall lead time at pre-defined timepoints is planned to facilitate decisions as to whether or not the trial will be continued in case of unexpected low ctDNA positivity rates or unexpected short lead times. While all analyses regarding secondary objectives will have exploratory and hypothesis-generating character only, they will provide a wealth of important data regarding the usability of different liquid biopsy markers in the context of breast cancer surveillance and MRD detection following primary therapy in breast cancer patients.

No formal efficacy interim analysis with specifically defined stopping rules is planned. An informal, non-binding interim analyses of QoL will be performed one year after 50% of patients (n = 1750) have been randomized. Based on the individual data from the PA-F12 and EORTC QLQ C30 questionnaires obtained from these patients 12 months after randomization, differences in QoL between the two randomization arms will be evaluated (with the main focus being fear of progression and depression) to ensure that patients in the Intensive Surveillance arm do not suffer from unacceptable emotional burden as compared to the standard arm. There will be no prespecified rules for stopping the trial due to concerns regarding emotional burden for patients in the experimental arm. However, the results of the QoL interim analysis will be presented to the Independent Data Monitoring Committee (IDMC) to evaluate whether a premature termination of the trial due to any concerns regarding major psychological or physical harms is deemed necessary.

Electronic data capture is performed using secuTrial® and statistical analyses will be conducted using the statistical software package IBM SPSS Statistics (IBM Corp., Armonk, N.Y., USA).

### Regulatory, dissemination and timeline

The trial has initially been approved by the Ethics Committee of the Ulm University (Nr 341/22) on 19^th^ October 2022, and two amendments have been approved in March 2023 and May 2024 comprising among several smaller changes and modifications the extension of the enrollment period of hormone receptor positive breast cancer patients. The study will be conducted at over 100 centers which are distributed all over Germany to assure the availability to the majority of breast cancer survivors.

First patient in was Q4/2022, and with an estimated recruitment period of four years, last patient in is predicted for Q4/2026. Accordingly, end of intervention is predicted for Q4/2031. As there will be a follow-up of further five years after the end of intervention, last data will be collected approximately Q4/2036. Final results will be available after this time point.

## Discussion

Breast cancer is still ranked as number 4 in the world’s cancer mortality ranking [1] with over half a million deaths in 2022 even though there were significant improvements in the therapy of early and metastatic breast cancer.

At a localized stage, meaning the disease being confined to the breast and regional lymph nodes, mortality is significantly lower in comparison to metastatic disease. As an example, a study reported five-year OS rates of 99% for localized disease in the breast, 86% for involvement of regional lymph nodes, and only 27% for patients with distant metastases [26]. Therefore, one can hypothesize that early detection and treatment of low tumor burden could improve patient outcomes. As mentioned before, current aftercare guidelines recommend against screening for metastases in asymptomatic patients [2,3] because of old data showing no improvement of OS if metastases are diagnosed in an asymptomatic stage [4,5]. However, it is known that delayed first treatment after diagnosis is associated with worse survival rate in breast cancer patients [27].

During the last decades, primary breast cancer treatment has become more and more individualized, considering tumor-specific factors, e.g., hormone and HER2 receptor status, TNM classification, gene expression tests, and patient-specific factors, e.g., germline mutations, comorbidities, and biological age. This is leading to a personalized concept of primary therapy in breast cancer, which aims to ensure the best therapeutic concept for the individual patient regarding disease-free and overall survival but also quality of life. However, after completion of this individualized primary therapy, all patients go through the same standard aftercare, largely neglecting any remaining interindividual differences regarding tumor biology and recurrence risk.

Liquid biopsy-guided surveillance in medium- and high-risk breast cancer survivors could be an important step toward individualizing the follow-up period, enabling earlier detection of metastases and allowing treatment to begin before symptoms develop. The biomarkers most commonly used in clinical routine are serum tumor markers. In the case of CA15.3 and CEA, it has been shown that prediction of distant recurrence before the onset of symptoms or (imaging) tests after primary and/or adjuvant therapy is possible [28]. However, sensitivity and specificity of tumor markers are known to be too low for a sensible screening regarding breast cancer [29]. CTCs are also known to be a negative prognostic marker [12] but are currently not commonly used in clinical practice. Regarding ctDNA, several published studies showed the high sensitivity and specificity of ctDNA in predicting distant metastases. These studies have been summarized in a recent meta-analysis, reporting a mean lead time (i.e., the time from first detection of ctDNA to the diagnosis of clinical/radiological relapse) of 10.8 months with a range from 0 to nearly 59 months [15]. Regarding the discovery of local recurrence [16] or brain-only metastases [17], the ctDNA tests so far showed less promising detection results; however, local recurrence is usually detected by the regular performance of mammography and ultrasound of the breast that is already performed in current aftercare.

The SURVIVE trial will serve to collect important new data regarding sensitivity and specificity of these liquid biopsy markers (separately and in combination) with respect to the early detection of metastatic spread before the onset of clinical symptoms in breast cancer patients.

### Limitations and risks of the study design

In the clinical routine, serum tumor marker testing during aftercare is still in discussion [30] and in some countries like Germany there are well established guidelines advising against this approach in the absence of symptoms [31]. Nevertheless, patients as well as doctors are frequently inclined to ask for or perform tumor marker testing. This is the reason why we integrated serum tumor markers in addition to CTCs and ctDNA in the liquid biopsy marker panel used for the assessment of molecular disease in the SURVIVE trial.

Intensified surveillance strategies inherently carry certain risks, including increased cumulative radiation exposure, the potential for treatment-related morbidity resulting from invasive diagnostic procedures, and the considerable psychological burden associated with recurrent false alarms and ongoing uncertainty.

While the biomarkers used in this trial have varying rates of specificity and sensitivity (see above), an imaging is triggered by any positive liquid biopsy result. Thus, for example even a rather unspecific rise in serum tumor markers could lead to several subsequent CT scans with no detectable metastatic disease. In addition, given that the lead time of ctDNA has been shown to be as long as three years, a positive ctDNA result can also lead to several CT scans that have to be performed regularly, before distant metastases are detected via imaging.

Several studies have shown that scan-associated anxiety can cause significant distress among cancer survivors [32]. These concerns were thoroughly discussed with patient representatives to ensure that their lived experiences and preferences were incorporated into the study design. Ultimately, a consensus emerged that the potential benefits of intensified surveillance—particularly the psychological reassurance it provides and the reduction in anxiety associated with less frequent follow-up—were judged to outweigh the associated risks. Moreover, we tried to minimize unnecessary imaging by integrating several biomarker-specific algorithms defining a maximum number of CT scans per time allowed and monitor fear and stress levels of the patients using the fear of progression questionnaire PA-F12.

Increasing the number of CT scans inevitably exposes patients to a higher cumulative radiation dose, thereby elevating the risk of various long-term complications, including, among others, radiation-induced secondary malignancies [33]. However, it is important to emphasize that the staging investigations in this study are not performed routinely but are instead triggered exclusively by abnormal findings in the liquid biopsy. This is therefore not a study based on regular imaging intervals. Rather, it is anticipated that, within a defined lead time, metastatic disease will become radiologically detectable following a pathological liquid biopsy result. A key objective of the study is to demonstrate that liquid biopsy can facilitate the early identification of patients with incipient metastatic disease, thereby enabling timely intervention—potentially with a curative intent.

There is evidence that early treatment of oligometastatic breast cancer with curative intention demonstrates no survival benefit in terms of OS or PFS [34]. While other studies involving different cancer types have demonstrated a survival benefit of additional radiotherapy targeting oligometastatic disease, they have also highlighted the potential risks, particularly treatment-associated morbidity and even mortality [35,36]. However, in the context of an increasingly dynamic therapeutic landscape—characterized by the continuous development of novel and more precisely targeted treatments—further investigation of this therapeutic concept remains highly relevant and warranted.

This trial is only partially blinded, as patients in the intensive surveillance arm with a positive liquid biopsy result are informed and advised to undergo staging imaging (patients in the intensive surveillance arm without any positive liquid biopsy result and all patients in the standard surveillance arm will remain blinded throughout the study). While blinding is important to reduce and prevent bias, one could argue that patients in the standard surveillance group might have a false sense of security, believing in having negative liquid biopsy results as no information to the patient regarding the results takes place. After extensive discussions with patients’ representatives during the phase of protocol development, we reached the consensus that this is an acceptable risk the patients should we willing to take, given both the detailed information regarding this aspect in the informed consent form and a thorough education and clarification during the enrollment process.

Another potential limitation of the study lies in the heterogeneity of the study population. Patients with different breast cancer subtypes—each associated with distinct biological behavior and recurrence risk—are included at various time points during follow-up. This variability may introduce confounding factors and limit the comparability of outcomes across subgroups, potentially affecting the generalizability of the findings.

In conclusion, the SURVIVE trial investigates whether a liquid biopsy guided intensified surveillance following primary therapy improves overall survival in breast cancer patients. It is currently the only recruiting study in this setting and extent that has the potential to revolutionize current breast cancer aftercare. Furthermore, the concept of initiating therapeutic interventions based on the detection of molecular disease prior to radiologically confirmed metastases is gaining increasing attention across various malignancies. For example, in colorectal cancer, ctDNA is being used to identify patients at high risk of relapse and to guide adjuvant therapy decisions, as demonstrated by the DYNAMIC trial [37]. In breast cancer, upcoming studies such as the SURVIVE HERoes trial (NCT06643585) and the TREAT ctDNA trial (NCT05512364) will investigate the efficacy of different agents such as Trastuzumab Deruxtecan and Elacestrant in patients with MRD detected by ctDNA, aiming to prevent the development of metastatic disease before it becomes visible on imaging. These promising approaches highlight the potential of molecular surveillance to transform early cancer management and improve patient prognosis. Should the study demonstrate a survival benefit, it could revolutionize breast cancer follow-up care and significantly improve the long-term prognosis for women diagnosed with breast cancer.

## Supporting information

S1 Checklist SURVIVE SPIRIT-Checklist. (PDF)

S1 File SURVIVE Model Consent Form. (PDF)

S2 File Protocol SURVIVE Master v1.0 14.09.2022. (PDF)

S3 File Protocol SURVIVE Master v2.0 02.03.2023. (PDF)

S4 File Protocol SURVIVE Master v3.0 13.05.2024. (PDF)

S2 Checklist SURVIVE Human Participants Research Checklist. (PDF)

## References

[1] https://gco.iarc.who.int/media/globocan/factsheets/cancers/20-breast-fact-sheet.pdf Accessed 2025 January 4.

[2] Khatcheressian JL, Hurley P, Bantug E, Esserman LJ, Grunfeld E, Halberg F, et al. Breast cancer follow-up and management after primary treatment: American Society of Clinical Oncology clinical practice guideline update. J Clin Oncol. 2013;31(7):961–5. doi: 10.1200/JCO.2012.45.9859 23129741

[3] Loibl S, André F, Bachelot T, Barrios CH, Bergh J, Burstein HJ, et al. Early breast cancer: ESMO clinical practice guideline for diagnosis, treatment and follow-up. Ann Oncol. 2024;35(2):159–82. doi: 10.1016/j.annonc.2023.11.016 38101773

[4] Rosselli Del Turco M, Palli D, Cariddi A, Ciatto S, Pacini P, Distante V. Intensive diagnostic follow-up after treatment of primary breast cancer. A randomized trial. national research council project on breast cancer follow-up. JAMA. 1994;271(20):1593–7. doi: 10.1001/jama.271.20.1593 7848404

[5] Impact of follow-up testing on survival and health-related quality of life in breast cancer patients. A multicenter randomized controlled trial. The GIVIO Investigators. JAMA. 1994;271(20):1587–92. doi: 10.1001/jama.1994.03510440047031 8182811

[6] Rojas MP, Telaro E, Russo A, Moschetti I, Coe L, Fossati R, et al. Follow-up strategies for women treated for early breast cancer. Cochrane Database Syst Rev. 2005;(1):CD001768. doi: 10.1002/14651858.CD001768.pub2 15674884

[7] Piccart-Gebhart MJ, Procter M, Leyland-Jones B, Goldhirsch A, Untch M, Smith I, et al. Trastuzumab after adjuvant chemotherapy in HER2-positive breast cancer. N Engl J Med. 2005;353(16):1659–72. 16236737 10.1056/NEJMoa052306

[8] Slamon D, Lipatov O, Nowecki Z, McAndrew N, Kukielka-Budny B, Stroyakovskiy D, et al. Ribociclib plus Endocrine Therapy in Early Breast Cancer. N Engl J Med. 2024;390(12):1080–91. doi: 10.1056/NEJMoa2305488 38507751

[9] Rastogi P, O’Shaughnessy J, Martin M, Boyle F, Cortes J, Rugo HS, et al. Adjuvant abemaciclib plus endocrine therapy for hormone receptor-positive, human epidermal growth factor receptor 2-negative, high-risk early breast cancer: results from a preplanned monarchE overall survival interim analysis, including 5-Year efficacy outcomes. J Clin Oncol. 2024;42(9):987–93. doi: 10.1200/JCO.23.01994 38194616 PMC10950161

[10] Mark C, Lee JS, Cui X, Yuan Y. Antibody-drug conjugates in breast cancer: current status and future directions. Int J Mol Sci. 2023;24(18):13726. doi: 10.3390/ijms241813726 37762027 PMC10531043

[11] Janni WJ, Rack B, Terstappen LWMM, Pierga J-Y, Taran F-A, Fehm T, et al. Pooled Analysis of the Prognostic Relevance of Circulating Tumor Cells in Primary Breast Cancer. Clin Cancer Res. 2016;22(10):2583–93. doi: 10.1158/1078-0432.CCR-15-1603 26733614

[12] Rack BK, Schindlbeck C, Andergassen U, Schneeweiss A, Zwingers T, Lichtenegger W, et al. Use of circulating tumor cells (CTC) in peripheral blood of breast cancer patients before and after adjuvant chemotherapy to predict risk for relapse: The SUCCESS trial. JCO. 2010;28(15_suppl):1003–1003. doi: 10.1200/jco.2010.28.15_suppl.1003

[13] Trapp E, Janni W, Schindlbeck C, Jückstock J, Andergassen U, de Gregorio A, et al. Presence of Circulating Tumor Cells in High-Risk Early Breast Cancer During Follow-Up and Prognosis. J Natl Cancer Inst. 2019;111(4):380–7. doi: 10.1093/jnci/djy152 30312434

[14] Huebner H, Häberle L, Müller V, Schrader I, Lorenz R, Forstbauer H, et al. MUC1 (CA27.29) before and after Chemotherapy and Prognosis in High-Risk Early Breast Cancer Patients. Cancers (Basel). 2022;14(7):1721. doi: 10.3390/cancers14071721 35406491 PMC8997086

[15] Nader-Marta G, Monteforte M, Agostinetto E, Cinquini M, Martins-Branco D, Langouo M, et al. Circulating tumor DNA for predicting recurrence in patients with operable breast cancer: a systematic review and meta-analysis. ESMO Open. 2024;9(3):102390. doi: 10.1016/j.esmoop.2024.102390 38460249 PMC10940943

[16] Lipsyc-Sharf M, de Bruin EC, Santos K, McEwen R, Stetson D, Patel A, et al. Circulating tumor DNA and late recurrence in high-risk Hormone receptor-positive, human epidermal growth factor receptor 2-negative breast cancer. J Clin Oncol. 2022;40(22):2408–19. doi: 10.1200/JCO.22.00908 35658506 PMC9467679

[17] Garcia-Murillas I, Chopra N, Comino-Méndez I, Beaney M, Tovey H, Cutts RJ, et al. Assessment of Molecular Relapse Detection in Early-Stage Breast Cancer. JAMA Oncol. 2019;5(10):1473–8. doi: 10.1001/jamaoncol.2019.1838 31369045 PMC6681568

[18] Coombes RC, Page K, Salari R, Hastings RK, Armstrong A, Ahmed S, et al. Personalized detection of circulating tumor DNA Antedates Breast Cancer Metastatic Recurrence. Clin Cancer Res. 2019;25(14): 4255–63. 30992300 10.1158/1078-0432.CCR-18-3663

[19] Lin DC, Genzen JR. Concordance analysis of paired cancer antigen (CA) 15-3 and 27.29 testing. Breast Cancer Res Treat. 2018;167(1):269–76. doi: 10.1007/s10549-017-4513-0 28929449

[20] Lee JS, Park S, Park JM, Cho JH, Kim SI, Park BW. Elevated levels of preoperative CA 15-3 and CEA serum levels have independently poor prognostic significance in breast cancer. Ann Oncol. 2013;24(5):1225–31. 23230137 10.1093/annonc/mds604

[21] Hepp P, Andergassen U, Jäger B, Trapp E, Alunni-Fabbroni M, Friedl TWP, et al. Association of CA27.29 and Circulating Tumor Cells Before and at Different Times After Adjuvant Chemotherapy in Patients with Early-stage Breast Cancer - The SUCCESS Trial. Anticancer Res. 2016;36(9):4771–6. doi: 10.21873/anticanres.11034 27630326

[22] de Gregorio A, Häberle L, Fasching PA, Müller V, Schrader I, Lorenz R, et al. Gemcitabine as adjuvant chemotherapy in patients with high-risk early breast cancer-results from the randomized phase III SUCCESS-A trial. Breast Cancer Res. 2020;22(1):111. doi: 10.1186/s13058-020-01348-w 33097092 PMC7583247

[23] Stieber P, Nagel D, Blankenburg I, Heinemann V, Untch M, Bauerfeind I, et al. Diagnostic efficacy of CA 15-3 and CEA in the early detection of metastatic breast cancer-A retrospective analysis of kinetics on 743 breast cancer patients. Clin Chim Acta. 2015;448:228–31. doi: 10.1016/j.cca.2015.06.022 26160053

[24] Di Gioia D, Stieber P, Schmidt GP, Nagel D, Heinemann V, Baur-Melnyk A. Early detection of metastatic disease in asymptomatic breast cancer patients with whole-body imaging and defined tumour marker increase. Br J Cancer. 2015;112(5):809–18. doi: 10.1038/bjc.2015.8 25647014 PMC4453962

[25] Di Gioia D, Blankenburg I, Nagel D, Heinemann V, Stieber P. Tumor markers in the early detection of tumor recurrence in breast cancer patients: CA 125, CYFRA 21-1, HER2 shed antigen, LDH and CRP in combination with CEA and CA 15-3. Clin Chim Acta. 2016;461:1–7. doi: 10.1016/j.cca.2016.07.014 27451906

[26] Siegel RL, Miller KD, Jemal A. Cancer statistics, 2020. CA: A Cancer J Clinicians. 2020;70(1):7–30. 10.3322/caac.21590 31912902

[27] Ho PJ, Cook AR, Binte Mohamed Ri NK, Liu J, Li J, Hartman M. Impact of delayed treatment in women diagnosed with breast cancer: A population-based study. Cancer Med. 2020;9(7):2435–44. doi: 10.1002/cam4.2830 32053293 PMC7131859

[28] Lauro S, Trasatti L, Bordin F, Lanzetta G, Bria E, Gelibter A. Comparison of CEA, MCA, CA 15-3 and CA 27-29 in follow-up and monitoring therapeutic response in breast cancer patients. Anticancer Res. 1999;19(4C):3511–5. 10629644

[29] Zaleski M, Kobilay M, Schroeder L, Debald M, Semaan A, Hettwer K, et al. Improved sensitivity for detection of breast cancer by combination of miR-34a and tumor markers CA 15-3 or CEA. Oncotarget. 2018;9(32):22523–36. doi: 10.18632/oncotarget.25077 29854296 PMC5976482

[30] Harris L, Fritsche H, Mennel R, Norton L, Ravdin P, Taube S, et al. American society of clinical oncology 2007 update of recommendations for the use of tumor markers in breast cancer. J Clin Oncol. 2007;25(33):5287–312. doi: 10.1200/JCO.2007.14.2364 17954709

[31] S3-Leitlinie Mammakarzinom. Leitlinienprogramm Onkologie. 2021.

[32] Khatri R, Quinn PL, Wells-Di Gregorio S, Pawlik TM, Cloyd JM. Surveillance-associated anxiety after curative-intent cancer surgery: a systematic review. Ann Surg Oncol. 2025;32(1):47–62. 39343818 10.1245/s10434-024-16287-5 PMC11659363

[33] Brenner DJ, Hall EJ. Computed tomography--an increasing source of radiation exposure. N Engl J Med. 2007;357(22):2277–84. doi: 10.1056/NEJMra072149 18046031

[34] Chmura SJ, Winter K, Woodward W, Borges V, Salama J, Al-Hallaq H. NRG-BR002: A phase IIR/III trial of standard of care systemic therapy with or without stereotactic body radiotherapy (SBRT) and/or surgical resection (SR) for newly oligometastatic breast cancer (NCT02364557). JCO. 2022;40:1007–1007.

[35] Palma DA, Olson R, Harrow S, Gaede S, Louie AV, Haasbeek C, et al. Stereotactic ablative radiotherapy versus standard of care palliative treatment in patients with oligometastatic cancers (SABR-COMET): a randomised, phase 2, open-label trial. Lancet. 2019;393(10185):2051–8. doi: 10.1016/S0140-6736(18)32487-5 30982687

[36] Palma DA, Olson R, Harrow S, Gaede S, Louie AV, Haasbeek C, et al. Stereotactic Ablative Radiotherapy for the Comprehensive Treatment of Oligometastatic Cancers: Long-Term Results of the SABR-COMET Phase II Randomized Trial. J Clin Oncol. 2020;38(25):2830–8. doi: 10.1200/JCO.20.00818 32484754 PMC7460150

[37] Tie J, Cohen JD, Lahouel K, Lo SN, Wang Y, Kosmider S, et al. Circulating tumor DNA analysis guiding Adjuvant therapy in stage II colon cancer. N Engl J Med. 2022;386(24):2261–72. doi: 10.1056/NEJMoa2200075 35657320 PMC9701133

