# Supplementary material for: The SURVIVE study (NCT05658172): Bringing breast cancer aftercare to the 21stcentury: Study protocol of a Phase III clinical trial comparing liquid biopsy guided vs. Standard of care surveillance for intermediate to high-risk breast cancer survivors
Source: PLoS One. 2025 Sep 9;20(9):e0331203. doi: 10.1371/journal.pone.0331203 (PMC12419582; doi:10.1371/journal.pone.0331203)
Supplement: S1 File — (PDF) [file pone.0331203.s002.pdf]

PATIENTINNENINFORMATION UND EINWILLIGUNGSERKLÄRUNG

SURVIVE (Standard Nachsorge im Gegensatz zu einer intensivierten Nachsorge bei PatientInnen mit früher Brustkrebserkrankung)
– eine teilweise doppel-blinde, multizentrische, randomisierte, kontrollierte Überlegenheitsstudie

SURVIVE Studie

Studienzentrum:	
Prüfarzt:	
Zentrumsnummer:	
Notfalltelefon:	

Sehr geehrte/r PatientIn,

Ihr behandelnder Arzt hat Ihnen die Teilnahme an einer klinischen Studie vorgeschlagen, die wir Ihnen im Folgenden erläutern möchten.

Die häufigste bösartige Erkrankung der Frau ist der Brustkrebs. Ungefähr jede 8. Frau erkrankt im Laufe ihres Lebens an dieser Erkrankung. Männer erkranken deutlich seltener, so ist etwa jeder 800. Mann betroffen. Die Prognose ist erfreulicherweise meist sehr gut, da die Erstdiagnose häufig in einem frühen Stadium, sprich in einer Situation, in welcher der Krebs noch nicht gestreut hat, gestellt wird. Diese nicht nur in höherem Alter, sondern auch bei jungen Frauen auftretende Erkrankung bedarf jedoch einer langfristigen Nachsorge.

Während jede Brustkrebserkrankung nach der Erstdiagnose zunächst individuell und entsprechend des Risikos behandelt wird, erfolgt die Nachsorge bei Brustkrebs für jede/jeden PatientIn gleich, unabhängig vom individuellen Risiko. Die nach den aktuellen Leitlinien (wie z. B. der AGO Mamma) durchgeführte Brustkrebs-Nachsorge basiert allerdings auf älteren Studiendaten, die nicht mehr in allen Punkten dem heutigen medizinischen Wissensstand entsprechen.

Diese Studie wird vom Universitätsklinikum Ulm, Klinik für Frauenheilkunde und Geburtshilfe, Prittwitzstraße 43, 89075 Ulm, Deutschland initiiert, der Sponsor ist das Universitätsklinikum Ulm. Es handelt sich um eine vom Bundesministerium für Bildung und Forschung (BMBF) geförderten Studie, in der wir moderne Untersuchungsmethoden im Blut nutzen möchten, um zu überprüfen, ob ein an das individuelle Risiko angepasstes Nachsorgeprogramm Vorteile gegenüber einer Nachsorge gemäß der aktuellen Leitlinienempfehlung bringt.

Ihre schriftliche Einwilligungserklärung ist unabdingbar für alle weiteren Schritte. Erst wenn Sie die Einwilligungserklärung unterschrieben haben, darf Ihr Prüfarzt mit der Eintragung von Untersuchungsergebnissen im Dokumentationssystem der Studie beginnen. Dabei darf er alle bereits bekannten Werte aus der Vergangenheit, die für den Einschluss in die Studie bedeutsam sind, für die Studie verwenden. Das erspart Ihnen zusätzliche oder sogar doppelt durchgeführte Untersuchungen.

Studienablauf

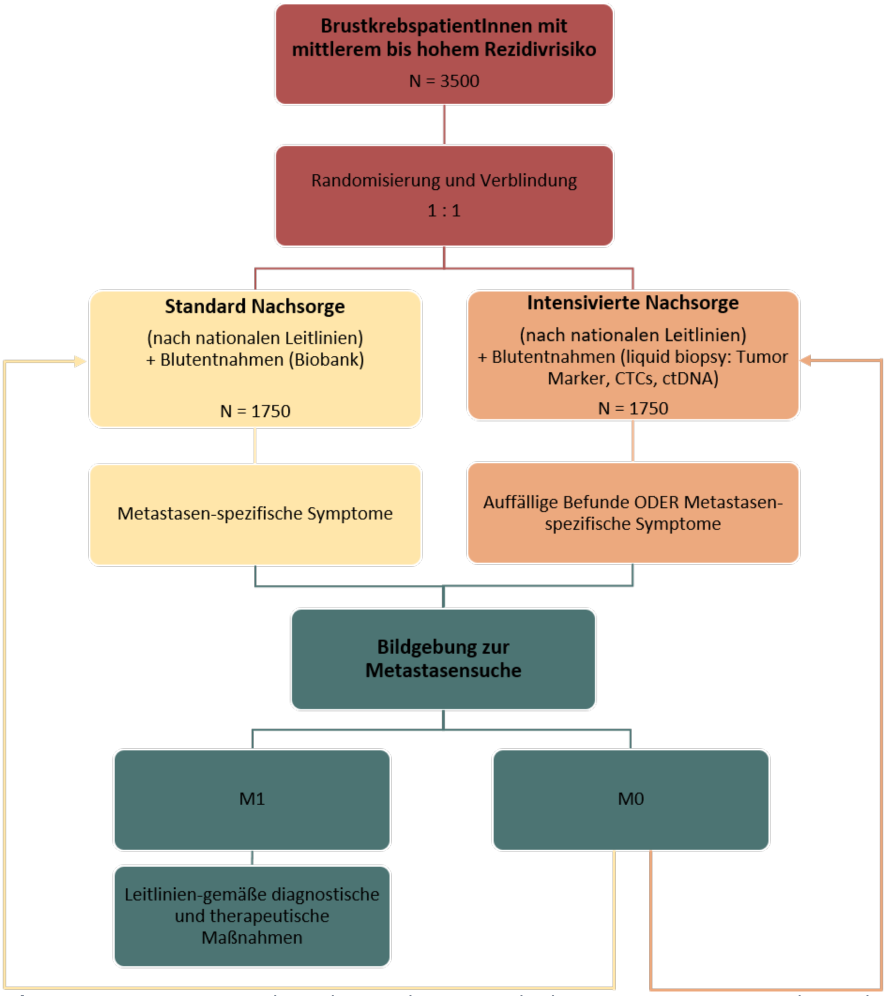

Abbildung 1: Studiendesign – Die SURVIVE Studie richtet sich an BrustkrebspatientInnen mit mittlerem bis hohem Rezidivrisiko. Insgesamt sollen 3500 PatientInnen teilnehmen. Zu Beginn Ihrer Studienteilnahme erfolgt eine zufällige Zuteilung (Randomisierung) (rot) in einen von 2 Studienarmen: Standardnachsorge (gelb) oder Intensivierte Nachsorge (orange). Im **Standardnachsorgearm** werden Sie gemäß den aktuellen Empfehlungen für die Nachsorge von BrustkrebspatientInnen untersucht und die im Rahmen der Studie abgegebenen Blutproben werden ohne weitere Untersuchung aufbewahrt. Eine weiterführende Bildgebung (z.B. eine Computertomographie (CT), Magnetresonanztomographie (MRT), Röntgen, Knochenszintigraphie, etc.) (grün) wird symptomorientiert durchgeführt. Im **Intensivierten Nachsorgearm** werden Sie gemäß den aktuellen Empfehlungen für die Nachsorge von BrustkrebspatientInnen untersucht. Zusätzlich werden die abgegebenen Blutproben auf das Vorliegen von Tumormarkern (CA27.29, CEA, CA125), zirkulierenden Tumorzellen (CTCs) und zirkulierender Tumor-DNA (ctDNA) untersucht. Sollte sich hier ein auffälliger Befund ergeben, dann erfolgt eine weiterführende Untersuchung mittels Computertomographie von Brust und Bauchraum (CT-Thorax/Abdomen) sowie Knochenszintigraphie zum Ausschluss einer erneuten Erkrankungsmanifestation. Für beide Studienarme gilt: sollte sich bei der Bildgebung eine erneute Erkrankungsmanifestation zeigen (M1), so werden die Leitlinien-gemäßen diagnostischen und therapeutischen Maßnahmen eingeleitet. Sollte die Bildgebung unauffällig sein (M0), so werden die regulären Blutentnahmen fortgeführt.

Wenn Sie an dieser Studie teilnehmen, dann werden Sie sich, gemäß den aktuellen Empfehlungen für die Nachsorge von BrustkrebspatientInnen, in den ersten drei Jahren nach Beendigung der Therapie Ihrer Brustkrebserkrankung, und nach Beginn der Studie alle 3 Monate und in den folgenden 2 Jahren alle 6 Monate bei Ihrem Frauenarzt zur (klinischen) Untersuchung vorstellen. Kontrolluntersuchungen der Brust erfolgen ebenso analog der aktuellen Empfehlung mit einer Mammographie (in Ergänzung ggfs. Ultraschall/Kernspintomographie) jährlich.

Zusätzlich und über die aktuellen Empfehlungen hinaus, werden Sie sich in Ihrem Prüfzentrum zur Blutabnahme vorstellen.

Diese umfasst je nach Zeitpunkt maximal 57,5 ml Blut (2 x 10 ml Streck Cell Free DNA BCT® ± 7,5 ml Serum ± 3 x 10 ml CellSave). Generell kann es möglich sein, dass nach ärztlichem Ermessen noch weitere Blutproben für nicht studienbedingte klinische Untersuchungen entnommen werden (z.B. zur Beurteilung von

Therapienebenwirkungen). Zusätzlich werden Sie dazu gebeten, standardisierte Fragebögen zu Ihrer Lebensqualität zu beantworten. Beim Besuch im Studienzentrum werden Sie auch zu Ihren aktuellen Medikamenteneinnahmen, zu Untersuchungen und Untersuchungsergebnissen, welche parallel zur Studie erhoben wurden, befragt.

Zu Beginn Ihrer Studienteilnahme erfolgt eine zufällige Zuteilung (Randomisierung) in einen von 2 Studienarmen:

Studienarm A (Standardarm): Sie werden gemäß den aktuellen Empfehlungen für die Nachsorge von BrustkrebspatientInnen untersucht und die abgegebenen Blutproben werden ohne weitere Untersuchung aufbewahrt. Blutproben sollen auch über das Studienende hinaus aufbewahrt werden, um für weitere zukünftige wissenschaftliche Fragestellungen genutzt werden zu können (siehe Punkt „Was passiert mit meinen Blut- und Gewebeproben?"). Eine weiterführende Diagnostik (z.B. eine Computertomographie (CT), Magnetresonanztomographie (MRT), Röntgen, Knochenszintigraphie, etc.) wird gemäß den aktuellen Leitlinien symptomorientiert durchgeführt.

Studienarm B (Interventionsarm): Sie werden gemäß den aktuellen Empfehlungen für die Nachsorge von BrustkrebspatientInnen untersucht. Zusätzlich der oben genannten Maßnahmen werden die abgegebenen Blutproben auf das Vorliegen von Tumormarkern (CA27.29, CEA, CA125), zirkulierenden Tumorzellen (CTCs) und zirkulierender Tumor-DNA (ctDNA) untersucht. Sollte sich hier ein auffälliger Befund ergeben (CTC/ctDNA) oder der Wert im Vergleich zur Voruntersuchung relevant steigen (Tumormarker), dann erfolgt eine weiterführende Untersuchung mittels Computertomographie von Brust und Bauchraum (CT-Thorax/Abdomen) sowie Knochenszintigraphie zum Ausschluss einer erneuten Erkrankungsmanifestation. Es ist zudem möglich, dass Blutproben analog zu Studienarm A auch über das Studienende hinaus aufbewahrt und im Verlauf für weitere Fragestellungen untersucht werden (siehe Punkt „Was passiert mit meinen Blut- und Gewebeproben?").

Sie und Ihr behandelnder Arzt werden nicht darüber informiert, in welchem Studienarm Sie sich befinden („Verblindung"), da das Wissen hierüber die Studienergebnisse beeinflussen könnte. (Dies ist auch der Grund für die Blutprobenentnahme im Standardarm A). Somit ist es auch nicht möglich, Ihnen die Ergebnisse der einzelnen Blutuntersuchungen mitzuteilen. Sollte es jedoch im Rahmen der Studie aufgrund der Untersuchungsergebnisse der Blutprobe notwendig sein, weitere Untersuchungen in die Wege zu leiten, wie z. B. eine Bildgebung, so ist dies nur in Studienarm B vorgesehen. In so einem Fall werden Sie erfahren, dass Sie dem interventionellen Studienarm zugeteilt sind.

Welche Risiken entstehen bei einer Studienteilnahme?

Die Risiken einer Studienteilnahme umfassen vor allem die Risiken einer konventionellen Blutentnahme (Schmerzen, Schwellungen, verstärkte Blutungen, Entwicklung eines blauen Flecks, Vernarbungen, Entzündungen, einer sehr seltenen Nervenschädigung, evtl. sogar mit chronischem Verlauf, aber auch Kreislaufbeschwerden bis hin zu Bewusstseinsverlust). Geläufige Maßnahmen um dies zu verhindern, wie Desinfektion der Blutentnahmestelle oder die Anwendung von Druck nach der Blutentnahme um Blutungen zu minimieren, werden durchgeführt.

Ein Nachteil bei Studienteilnahme könnte sein, dass Sie zusätzliche Zeit investieren müssen, um regelmäßige Visiten in Ihrem Sie betreuenden Studienzentrum wahrzunehmen. Eventuell werden Sie dadurch immer wieder daran erinnert, dass Sie an einer potentiell bedrohlichen Krebserkrankung gelitten haben. Dies geschieht zu einem Zeitpunkt, zu dem andere, nicht an der Studie teilnehmende PatientInnen, zur Nachsorge alleine zu ihrem sie behandelnden Arzt gehen. Dies könnte zu einer Verschlechterung der subjektiv empfundenen Lebensqualität

führen. Außerdem ist es möglich, dass eine Blutuntersuchung bei Ihnen zu einem positiven Testergebnis führt, ohne dass bei Ihnen in einer der daraufhin folgenden Untersuchungen mit bildgebenden Methoden Hinweise auf ein Fortschreiten Ihrer Krebserkrankung gefunden werden („falsch positives" Testergebnis). Da Sie im Rahmen der Studie über positive Testergebnisse informiert werden, könnte dies Ihre Ängste vor einem Rückfall erhöhen und damit zu einer Verschlechterung der subjektiv empfundenen Lebensqualität führen.

Wir haben die potentiellen Risiken und Vorteile einer Studienteilnahme sorgfältig abgewogen und kommen zu dem Schluss, dass bei einer Studienteilnahme die Vorteile die Risiken und Nachteile sowohl kurz- als auch langfristig überwiegen und eine Teilnahme an der SURVIVE-Studie deshalb medizinisch gerechtfertigt ist.

	Prüfzentrum			Nachsorgearzt		
	**Jahr 1–3**	**Jahr 4–5**	**Jahr 6–10**	**Jahr 1–3**	**Jahr 4–5**	**Jahr 6–10**
**Studienarm A**						
**Klinisch-orientierende Brustuntersuchung**				alle 3 Monate	alle 6 Monate	1x jährlich
**Blutentnahme (Aufbewahrung)** [1)]	alle 3 Monate	alle 6 Monate	-			-
**Blutentnahme (außerhalb Studie)**				symptomorientiert	symptomorientiert	symptomorientiert
**Mammographie und Ultraschall**				1x jährlich	1x jährlich	1x jährlich
**Erweiterte Bildgebung**				symptomorientiert	symptomorientiert	symptomorientiert
**Fragebögen (Lebensqualität, Aktuelles)**	alle 6 Monate	alle 6 Monate	1 x jährlich			
**Nachbeobachtung** [3)]						
**Studienarm B**						
**Klinisch-orientierende Brustuntersuchung**				alle 3 Monate	alle 6 Monate	1x jährlich
**Blutentnahme (TM, CTCs, ctDNA)** [1)]	alle 3 Monate	alle 6 Monate				-
**Blutentnahme (außerhalb Studie)**				symptomorientiert	symptomorientiert	symptomorientiert
**Mammographie und Ultraschall**				1x jährlich	1x jährlich	1x jährlich
**Erweiterte Bildgebung**	entsprechend Laborergebnis	entsprechend Laborergebnis		symptomorientiert	symptomorientiert	symptomorientiert
**Fragebögen (Lebensqualität, Aktuelles)**	alle 6 Monate	alle 6 Monate	1x jährlich			
**Nachbeobachtung** [3)]						
	1) Die studienspezifische Blutentnahme erfolgt obligat am Prüfzentrum. Die regulären Nachsorgeuntersuchungen finden weiter beim nachsorgenden Arzt (Frauenarzt, Onkologe, Hausarzt, etc.) statt.					

Unabhängig davon, welchem Studienarm Sie zugehörig sind, ist eine Erhebung der Lebensqualität mittels Fragebögen (in dieser Studie der EORTC-QLQ-C30 und der PA-F-12) vorgesehen um eventuell entstehende Belastungssituationen aufgrund der Erkrankung, der Nachsorge, der Therapie und auch der zusätzlichen Untersuchungen im Rahmen der Studie zu erfassen. Das Ausfüllen dieser Fragebögen erfolgt in Papierform (ca. 10-12 min für den EORTC-QLQ-C30 und ca. 3-5 min für den PA-F-12). Außerdem werden Sie zu weiteren aktuellen Untersuchungsergebnissen und Therapien befragt. Die Abfrage dieser Fragebögen kann optional auch über eine digitale Gesundheitsapplikation erfolgen. Diese Applikation („App") kann über Handy, perspektivisch auch über Tablet und Computer geladen werden und kann das Studienzentrum und Sie bei der Terminplanung unterstützen.

Die Eingabe und Speicherung der mit Hilfe einer Studien-ID pseudonymisierten klinischen Basisdaten (Pseudonymisierung: Ersetzen von Name und/oder anderen eindeutigen Identifikationsmerkmalen durch ein Pseudonym - meist ein aus einer Buchstaben- oder Zahlenkombination bestehender Code - um die Feststellung der Identität eines Patienten/einer Patientin auszuschließen oder wesentlich zu erschweren) erfolgt im Health Care Portal durch das Studienpersonal der Studienzentren. Die Daten werden auf einem nach ISO-27001 und ISO/IEC 20000 zertifizierten Rechenzentrum (Hetzner Cloud GmbH) mit Server-Standort in Deutschland gespeichert und gesichert. Ein Vertrag zur Auftragsverarbeitung zwischen dem IT-Dienstleister und Hetzner liegt vor. Die Datenübertragung erfolgt kryptographisch verschlüsselt über das https-Protokoll. Die Datenspeicherung erfolgt ebenfalls verschlüsselt. Die verschlossenen Serverräume und einzelne Server sind zugangskontrolliert.
In der Applikation („App") werden neben den Fragebögen personenbezogene Daten von Ihnen gespeichert, wie zum Beispiel Name, klinische IDs, E-Mail-Adresse, Telefonnummer (optional, wenn durch Sie hinterlegt), um die Kommunikation mit dem Studienteam zu gewährleisten (z. B. für Erinnerungen). Hierzu wird aus einem Programm angestoßen, das aus der separaten Datenbank („Personal DB") die erforderliche E-Mail-Adresse zu einer App-ID nutzt. Der IT-Dienstleister betreibt die Datenbank, welche bei der Hetzner Cloud GmbH liegt und auf einem dedizierten Server über eine Firewall abgesichert ist.
In die Auswertung der Studie fließen auch statistische Erhebungen zur Nutzung der Applikation ein. Für diesen Zweck werden folgende Daten erhoben: Anzahl und Dauer der Nutzung, Nutzung der Kommunikation, Nutzungsverhalten, Komplettierung der Fragebögen. Die Nutzerstatistiken der App-Benutzung für die Qualitätssicherung (Verfügbarkeit, App-Abstürze, Fehlerberichte) sowie die Evaluation werden ebenso ausschließlich aus pseudonymisierten Daten erstellt.

Sollte sich im Laufe der Studie eine Metastasierung zeigen, so ist für die betreffende Patientin / den betreffenden Patienten die Studie beendet und eine Therapie wird gemäß der aktuellen Leitlinie empfohlen. Ferner ist eine weitere Studienteilnahme nicht möglich, wenn das Primärtumorgewebe wiederholt nicht analysiert werden kann, der (Gesundheits-)Zustand einer Patientin / eines Patienten die weitere Studienteilnahme nicht ermöglicht oder wenn es neue Erkenntnisse hinsichtlich der Studienintervention oder Brustkrebserkrankungen gibt, welche eine weitere Studienfortführung nicht rechtfertigen. Außerdem erfolgt der Studienausschluss, wenn die Einwilligung der Patientin / des Patienten widerrufen wird.

Die geplante Studienteilnahmedauer beträgt insgesamt 10 Jahre – davon sind 5 Jahre Nachsorgeprogramm und Probenentnahme (wie bereits oben geschildert) und im Anschluss 5 Jahre Follow-Up (Nachbeobachtungsperiode).

Sollten sich im Laufe der Zeit Änderungen in der Nachsorge und Behandlung des Brustkrebses ergeben, so wird dies zukünftig im Rahmen der Studie berücksichtigt und mit aufgenommen. Dies könnte dazu führen, dass weitere Untersuchungen veranlasst werden. Sollten die Ergebnisse aus den Blutuntersuchungen die wissenschaftlich begründete Grundlage für eine Therapie oder die Teilnahme an einer weiteren Studie darstellen, werden wir Sie entsprechend informieren.

Was passiert mit meinen Blut- und Gewebeproben?

Im Interventionsarm (Studienarm B) wird zu Studienbeginn Ihr Tumorgewebe auf vorhandene individuelle Tumor-spezifische Marker genetisch untersucht. Anhand dieser Marker werden die im Verlauf der Studie wiederholt entnommenen Blutproben auf das Vorliegen von zirkulierender Tumor-DNA (ctDNA) untersucht. Zusätzlich werden in Ihrem Blut Tumormarker (CA27.29, CEA, CA125) und zirkulierende Tumorzellen (CTCs) analysiert.

Im Standardarm (Studienarm A) wird ebenfalls Blut entnommen, allerdings wird dieses aufbewahrt und nicht auf die oben genannten Parameter untersucht.

Unabhängig vom Studienarm kann es im Rahmen der Nachsorge ggf. notwendig sein, weitere Routinelaborparameter (z. B. Leber-, Nierenwerte oder Blutbild) zu bestimmen.

Das Material, welches im Rahmen der Studienuntersuchungen nicht verwendet wurde (aufbewahrte Blutproben aus Studienarm A und übrig gebliebenes Material aus Studienarm B) werden in einer so genannten Biobank aufbewahrt und für zukünftige translationale Forschung (Forschung, mit deren Hilfe die im Rahmen von Grundlagenforschung gewonnenen Erkenntnisse in die klinische Anwendung übertragen werden sollen) und retrospektive Analysen (Auswertungen, die erst nach erfolgter Datensammlung initiiert und durchgeführt werden) verwendet.

Ergebnisse aus derartigen Studien können wertvolle Erkenntnisse hinsichtlich weiterer Biomarker sowie über die Brustkrebsentstehung und den Krankheitsverlauf liefern. Auch die in Zukunft geplanten Forschungsprojekte möchten an diese Informationen anknüpfen und die Untersuchungen weiterführen. Ein weiteres Ziel der Forschung ist es, potentielle prädiktive Faktoren (d.h. Faktoren, welche es erlauben, den Verlauf der Brustkrebserkrankung bzw. das Ansprechen auf bestimmte Therapien vorherzusagen) zu identifizieren, die Einfluss auf den Krankheitsverlauf und Behandlungserfolg nehmen könnten. Hierfür sind diese aufbewahrten Blutproben ebenfalls wertvoll.

Wir fragen Sie nach einer Erlaubnis zur Verwendung Ihrer Bioproben und Daten. Diese werden für medizinische Forschung bereitgestellt, die die Vorbeugung, Erkennung und Behandlung von Erkrankungen verbessern soll. Sie sollen im Sinne eines möglichst großen Nutzens für die Allgemeinheit für viele verschiedene medizinische Forschungszwecke verwendet werden. Diese können sich sowohl auf bestimmte Krankheitsgebiete (z.B. Krebsleiden) als auch auf heute zum Teil noch unbekannte Krankheiten und genetische Zusammenhänge beziehen. Weil sich in der Forschung immer wieder neue Fragen ergeben, kann es sein, dass Ihre Proben und Daten auch für medizinische Forschungsvorhaben verwendet werden, die man heute noch nicht absehen kann. Ihre Bioproben und Daten werden nicht für Forschungsvorhaben verwendet, die von der Ethik-Kommission, die das Vorhaben bewertet, als unethisch erachtet werden. **Möglicherweise werden an Ihren Bioproben auch genetische Untersuchungen durchgeführt, und zwar unter Umständen auch eine Untersuchung Ihrer gesamten Erbsubstanz (Genom).**

Wichtig: Aus logistischen Gründen ist es der Biobank der SURVIVE-Studie nicht möglich, individuelle Eingrenzungen (z.B. Ausschluss bestimmter Forschung, Ausschluss der Weitergabe der Materialien an Dritte) vorzunehmen. **Wenn Sie mit der beschriebenen Art und Dauer der Nutzung nicht in vollem Umfang einverstanden sind, sollten Sie Ihre Einwilligung nicht erteilen.**

Diese wissenschaftlichen Untersuchungen sollen gegebenenfalls auf der Grundlage noch abzuschließender Verträge in Zusammenarbeit mit externen (Industrie-)Partnern durchgeführt werden. Wissenschaftliche Ergebnisse, die in Zusammenarbeit mit Industriepartnern gewonnen werden, sollen vor allem zur Entwicklung von neuen Medikamenten beitragen. Um eventuelle Entdeckungen möglichst effektiv für PatientInnen einsetzen zu

können, kann eine kommerzielle Nutzung der gewonnenen Ergebnisse erforderlich werden. Sofern dies rechtlich zulässig ist, wird die Universität Ulm Schutzrechte (Patente) begründen und industriellen Partnern Nutzungsrechte einräumen.

Für die Verwendung Ihres Gewebes im Rahmen von wissenschaftlichen Untersuchungen ist der Abschluss eines Übereignungsvertrags notwendig, der Ihnen mit dieser Patienteninformation durch Ihren behandelnden Arzt zur Unterschrift vorgelegt wird.

Die Analysen Ihres Tumorgewebes und im Rahmen der Analysen auf zirkulierende Tumor-DNA können neben den oben genannten Parametern auch die Keimbahn DNA (Ihre Erbsubstanz) und Proteine (Eiweiße) umfassen, sind aber nicht darauf beschränkt. Wir wissen heute, dass Gene eine Möglichkeit der Diagnosestellung im Hinblick auf das zukünftige Verhalten einer Krebserkrankung bieten können. Daher untersuchen wir Ihr Krebsgewebe genetisch. Sämtliche Zellen unseres Körpers erhalten Informationen, wie sie arbeiten und funktionieren sollen. Dies geschieht über ein Molekül, das als Desoxyribonukleinsäure (DNA) bezeichnet wird. Die DNA ist eine Liste mit Anweisungen, wobei die Anweisungen als Gene bezeichnet werden. Gene beeinflussen unser Wachstum und unsere Entwicklung. Keine andere Person besitzt genau die gleichen Gene wie Sie, es sei denn, Sie haben einen identischen Zwilling. Diese Unterschiede bedeuten, dass einige Menschen bestimmte Erkrankungen eher bekommen als andere. Einige Gene können bei mehr als einer Erkrankung wichtig sein. Gentests untersuchen Ihre Gene oder das genetische Profil Ihres Tumors entweder nur in einem Gen oder mehreren speziellen Genen oder allen Genen, also Ihre gesamte DNA. Gentests sind ein unerlässlicher Bestandteil dieser Prüfung. Wenn Sie keine Einwilligung in diese Tests erteilen möchten, können Sie an dieser Prüfung nicht teilnehmen. Alle Gentests, die im Rahmen dieser Studie an dem Tumorgewebe und den abgenommenen Blutproben durchgeführt werden, gelten rein zu Forschungszwecken und Sie erhalten diese Ergebnisse nicht. Auch werden diese Informationen auch nicht in Ihrer Krankenakte dokumentiert.

Die Proben werden für maximal 25 Jahre nach Studienende, also insgesamt 35 Jahre für jede Patientin / jeden Patienten die über die Gesamt-Studiendauer an den Visiten teilnimmt, aufbewahrt, um zu einem späteren Zeitpunkt Analysen zu Fragestellungen bei gleichen oder verwandten Krankheitsbildern durchführen zu können. Spätestens dann werden die Bioproben vernichtet und die Daten gelöscht. Ergebnisse aus derartigen Studien können eventuell wertvolle Erkenntnisse hinsichtlich weiterer Biomarker liefern.

Nach Entnahme wird die Blutprobe sofort mit einer Identifizierungsnummer versehen (pseudonymisiert), sodass Ihr Name und weitere personenbezogene Daten nicht mehr erkennbar sind. Ihre Proben werden, nach Abschluss des Übereignungsvertrages, Eigentum des Universitätsklinikums Ulm. Sie werden im Onkologischen Labor unter Verantwortung von Frau Prof. Wiesmüller aufbewahrt und unter Umständen für Untersuchungen an anderen Stellen in pseudonymisierter Form, gegebenenfalls auch ins europäische und nicht-europäische Ausland wie die Vereinigten Staaten von Amerika (USA), verschickt. Für die Weitergabe von Proben und Daten in Drittländer mit möglicherweise geringerem Datenschutzniveau benötigen wir Ihre ausdrückliche Zustimmung in der Einwilligungserklärung, da hier Ihre Rechte möglicherweise nicht gleichwertig durchgesetzt werden können wie in der Europäischen Union (EU). Die SURVIVE-Kooperationspartner im außereuropäischen Ausland werden jedoch geeignete Maßnahmen ergreifen, um einen vergleichbaren Datenschutzstandard wie in der EU zu gewährleisten. Die Universitätsfrauenklinik Ulm übernimmt hierfür als Studieninitiator die Verantwortung.

Dabei werden die gewonnenen Daten unter Umständen auch mit medizinischen Daten in anderen Datenbanken verknüpft, sofern die gesetzlichen Voraussetzungen hierfür erfüllt sind. Biomaterialien und Daten, die an Forscher herausgegeben wurden, dürfen nur für den vorbestimmten Forschungszweck verwendet und vom Empfänger nicht zu anderen Zwecken weitergegeben werden. Nicht verbrauchtes Material wird an den Sponsor (Universitätsklinikum Ulm) zurückgegeben. Gleichwohl haben Sie jederzeit das Recht beim Sponsor, die Vernichtung Ihrer Proben zu verlangen. Ergebnisse aus schon stattgefundenen Analysen dürfen mit Ihrer Zustimmung dann auch weiterhin in pseudonymisierter Form verwendet werden.

Die Untersuchung Ihrer Blutproben kann zur Entwicklung von Ergebnissen führen, die von kommerziellem Wert

für den Sponsor oder die beteiligten Unternehmen sein können. Der Sponsor sieht weder heute noch in der Zukunft vor, Ihnen eine Entschädigung, Nutzungsgebühr oder einen anderweitigen geldwerten Vorteil zu zahlen, der sich aus einem Artikel, Verfahren oder einem anderen Produkt ergeben könnte, das aus der Forschung an Ihren Proben, Informationen oder Daten, die von einer derartigen Forschung abgeleitet werden, hervorgeht.

Welchen Nutzen habe ich oder die Allgemeinheit durch die Lagerung der Bioproben (Biobank)?

Persönlich können Sie für Ihre Gesundheit keinen unmittelbaren Vorteil oder Nutzen aus der Spende Ihrer Proben und Daten erwarten. Deren Auswertung dient ausschließlich Forschungszwecken und nicht dazu, Rückschlüsse auf Ihre Gesundheit zu ziehen.

Es ist jedoch im Einzelfall möglich, dass ein Forscher zu der Einschätzung gelangt, dass ein Auswertungsergebnis für Ihre Gesundheit von erheblicher Bedeutung sein könnte. Das ist insbesondere der Fall, wenn sich daraus ein dringender Verdacht auf eine schwerwiegende, bisher möglicherweise nicht erkannte Krankheit ergibt, die behandelt oder deren Ausbruch verhindert werden könnte. In einem solchen Fall kann eine Rückmeldung an Sie erfolgen. Bitte kreuzen Sie in der Einwilligungserklärung an, ob Sie in einem solchen Fall eine Rückmeldung erhalten möchten (siehe Einwilligungserklärung). Sie können Ihre Entscheidung für oder gegen eine Rückmeldungsmöglichkeit jederzeit durch Mitteilung an uns ändern. Beachten Sie dabei jedoch, dass Sie Gesundheitsinformationen, die Sie durch eine solche Rückmeldung erhalten, unter Umständen bei anderen Stellen (z.B. vor Abschluss einer Kranken- oder Lebensversicherung) offenbaren müssen und dadurch Nachteile erleiden können.

Mit der Überlassung der Bioproben an die SURVIVE-Studie, werden diese Eigentum der im Rahmen der SURVIVE-Studieentstehenden Biobank. Ferner ermächtigen Sie die SURVIVE-Studieninitiatoren Ihre Daten zu nutzen. Für die Überlassung Ihrer Bioproben und Daten erhalten Sie kein Entgelt. Sollte aus der Forschung ein kommerzieller Nutzen erzielt werden, werden Sie daran nicht beteiligt.

Für die Allgemeinheit zielen medizinisch-wissenschaftliche Forschungsvorhaben auf eine Verbesserung unseres Verständnisses der Krebsentstehung und der Diagnosestellung und auf dieser Basis auf die Entwicklung von verbesserten Behandlungs- und Vorbeugungsmaßnahmen.

Wer hat Zugang zu den Bioproben und Daten der Biobank?

Voraussetzung für die Verwendung der Bioproben und Daten für ein konkretes medizinisches Forschungsprojekt ist grundsätzlich, dass das Forschungsvorhaben durch eine Ethik-Kommission bewertet wurde.

Sollten sich relevante Ergebnisse zeigen, so erfolgen wissenschaftliche Veröffentlichungen ausschließlich anonymisiert, also in einer Form, die keine Rückschlüsse auf Ihre Person zulässt. Das gilt insbesondere auch für genetische Informationen. Möglich ist allerdings eine Aufnahme genetischer Informationen in besonders geschützte wissenschaftliche Datenbanken, die für die Allgemeinheit nicht zugänglich sind.

Ist meine Studienteilnahme freiwillig und kann ich meine Einwilligung widerrufen?

Freiwilligkeit:

An diesem Forschungsprojekt nehmen Sie freiwillig teil. Ihre Einwilligung können Sie jederzeit und ohne Angabe von Gründen widerrufen.

Widerrufrecht:

Im Falle eines Widerrufs haben Sie zwei Möglichkeiten: Entweder werden alle Ihre Daten, welche bis zum Zeitpunkt des Widerrufs erhoben wurden, gelöscht und entsprechende Proben vernichtet. Alternativ erlauben Sie uns, die bisher erhobenen Daten pseudonymisiert weiter zu verwenden und bereits abgenommene Proben aufzuheben und gegebenenfalls zu einem späteren Zeitpunkt auszuwerten.

Dieser eventuelle Widerruf hat keine Auswirkungen auf Ihre aktuelle und zukünftige medizinische Betreuung.

Wer entscheidet, ob ich aus der Studie ausscheide?

Die Teilnahme an dieser Studie ist freiwillig. Sie können jederzeit, auch ohne Angabe von Gründen, Ihre Teilnahme beenden, ohne dass Ihnen dadurch irgendwelche Nachteile bei Ihrer medizinischen Behandlung entstehen. In diesem Falle wird Sie der behandelnde Arzt nach bestem Wissen und Gewissen weiterbehandeln. Sie sollten jedoch Ihren behandelnden Prüfarzt von Ihrer Entscheidung in Kenntnis setzen.

Unter gewissen Umständen ist es aber auch möglich, dass der Prüfarzt oder der Sponsor entscheidet, Ihre Teilnahme an der klinischen Prüfung vorzeitig zu beenden, ohne dass Sie auf die Entscheidung Einfluss haben. Die Gründe hierfür können z. B., aber nicht ausschließlich sein:

- Ihre weitere Teilnahme an der klinischen Prüfung ist ärztlich nicht mehr vertretbar;
- Das Tumorgewebe ist nicht ausreichend für die Herstellung der Bluttests (zum Beispiel bei vollständigem Ansprechen einer Chemotherapie, sogenannte pathologische Komplettremission)
- Bei Ihnen wird bildgebend eine Fernmetastasierung festgestellt;
- Die gesamte klinische Prüfung wird abgebrochen.

Der Prüfarzt wird mit Ihnen besprechen, wie und wo Ihre weitere Behandlung stattfindet.

Werden mir neue Erkenntnisse mitgeteilt und an wen wende ich mich bei Fragen?

Weitere Informationen, sowie Antworten auf Ihre Fragen zu dieser Studie oder zu Ihren Rechten als StudienteilnehmerIn erhalten Sie von Ihrem behandelnden Prüfarzt/Prüfärztin. Sie werden über alle wichtigen Erkenntnisse sofort informiert, die während des Verlaufs der Studie gewonnen werden und die Ihre Bereitschaft zur weiteren Teilnahme an dieser Studie beeinflussen könnten.

Sollten während des Verlaufes des Forschungsprojektes Fragen auftauchen, so können Sie jederzeit Frau Dr. Sophia Huesmann telefonisch unter 0731-500 58536 und per E-Mail über survive.studie@uniklinik-ulm.de erreichen. In Notfällen gilt folgende Nummer: 0731-500 58692.

Sie haben stets die Möglichkeit den auf Seite 1 genannten oder einen anderen Prüfarzt/Prüfärztin zu weiteren Beratungsgesprächen zu konsultieren.

Entstehen für mich durch die Teilnahme an der Studie zusätzliche Kosten und erhalte ich eine Aufwandsentschädigung?

Für die in der Studie notwendigen Untersuchungen (sowohl Untersuchung von Blutproben als auch ggf. erweiterte Bildgebung mittels z. B. Computertomographie, Knochenszintigraphie oder weitere Untersuchungen) fallen für Sie keine zusätzlichen Kosten an.

Sie erhalten keinerlei Aufwandsentschädigung für die Teilnahme an dieser Studie.

Bin ich während der Studie versichert?

Das Universitätsklinikum Ulm und dessen an der Studie mitwirkende Mitarbeiter (Studienärzte, sonstiges Personal) sind haftpflichtversichert für den Fall, dass Sie durch deren Verschulden einen Schaden erleiden. Ferner genießen Sie während der Teilnahme an dem Forschungsprojekt vom Sponsor (Universitätsklinikum Ulm) initiierten Versicherungsschutz. Dieser besteht für alle Gesundheitsschädigungen, die Folge von den bei dieser klinischen Studie angewandten Verfahren sind, oder die durch Maßnahmen hervorgerufen werden, die im Zusammenhang mit dieser klinischen Studie durchgeführt werden. Der Umfang des Versicherungsscheines beträgt pro PatientIn maximal 500.000,00 Euro.

Eine Wege- und Unfallversicherung besteht für Sie auf dem direkten Weg zum und vom Studienzentrum. Diese ist pro PatientIn auf 100.000,00 Euro limitiert.

Eine Gesundheitsschädigung, die mutmaßlich auf die klinische Studie zurückzuführen ist, muss innerhalb einer Woche dem Versicherer angezeigt werden. Dies kann durch Sie selbst oder über Ihren Prüfarzt in der Klinik erfolgen.

Gerne erhalten Sie eine Kopie der geltenden Versicherungsbedingungen, welcher Sie auch die Kontaktdaten des Versicherers entnehmen können.

Versicherer:	Chubb European Group SE, Direktion für Deutschland, Baseler Straße 10, 60329 Frankfurt am Main, Germany
Versicherungsschein Nummer:	DELSCA45932
Versicherungsnehmer:	Universitätsklinikum Ulm, Albert-Einstein-Allee 29, 89081 Ulm, Germany

Was geschieht mit meinen Daten und welche Rechte habe ich?

Datenverarbeitung und - Speicherung:
Alle Personen, welche Sie im Rahmen dieses Projekts betreuen, unterliegen der beruflichen Schweigepflicht nach § 203 StGB und sind auf das Datengeheimnis bzw. das Datenschutzgesetz verpflichtet, das es verbietet, persönliche oder gesundheitsbezogene Informationen an unbeteiligte Dritte weiterzugeben.

Während der Studie werden Gesundheitsdaten wie medizinische Befunde und genetische Daten sowie persönliche Informationen (wie Alter, Geschlecht, ethnische Herkunft) von Ihnen erhoben und in Ihrer persönlichen Akte niedergeschrieben oder elektronisch gespeichert. Die für die Studie wichtigen Daten werden zusätzlich in pseudonymisierter Form gespeichert, ausgewertet und ggf. weitergegeben. Pseudonymisierung bedeutet, persönliche Daten wie der Name und das Geburtsdatum z.B. durch einen Code zu ersetzen, wodurch eine direkte Zuordnung der erhobenen Daten zu einer konkreten Person durch Ditte verhindert wird. Ihre identifizierenden Daten werden hierfür durch einen Nummern- und/oder Buchstabencode ersetzt; die Angabe des Geburtsdatums wird auf das Geburtsjahr beschränkt. Ausschließlich in Ihrem individuellen lokalen Studienzentrum ist eine Liste hinterlegt, auf der Ihr Name dem Nummern- und/oder Buchstabencode zugeordnet ist. Das ist notwendig, damit Ihnen diese personenbeziehbaren Daten, falls erforderlich, durch das für Sie zuständige lokale Studienzentrum wieder zugeordnet werden können. Diese Liste wird im Studienzentrum gesondert aufbewahrt und unterliegt dort technischen und organisatorischen Maßnahmen, die gewährleisten, dass die identifizierenden Daten Ihnen durch unbefugte Personen nicht zugeordnet werden können.

Die Verwendung dieser studienbezogenen Daten erfolgt nach gesetzlichen Bestimmungen. Rechtsgrundlage für die Verarbeitung sind Ihre freiwillige Einwilligung gemäß Art. 6 Abs. 1 lit. a und Art. 9 Abs. 2 lit. a Datenschutzgrundverordnung (DSGVO) sowie Art. 6 Abs. 1 lit. c DSGVO.

Die Daten werden zu jeder Zeit vertraulich behandelt. Die Daten werden in pseudonymisierter Form an den Initiator der Studie (Universitätsklinikum Ulm, Klinik für Frauenheilkunde und Geburtshilfe, Prittwitzstraße 43, 89075 Ulm, Verantwortliche: Frau Dr. Sophia Huesmann) bzw. von ihr beauftragte Stellen zum Zweck der wissenschaftlichen Auswertung, der Korrelation von Befunden und zur Ergreifung von Maßnahmen, falls sich ein auffälliger Wert ergibt, weitergeleitet. Zugriff auf die identifizierenden Daten haben nur die zuständigen Personen im jeweiligen Studienzentrum.

In wissenschaftlichen Veröffentlichungen werden nur anonymisierte Daten verwendet, so dass ein Rückschluss auf einzelne StudienteilnehmerInnen nicht möglich ist.

Ihre Daten sind gegen unbefugten Zugriff Dritter gesichert. Sie werden nur in Übereinstimmung mit den in der Einwilligungserklärung genannten Bedingungen und den entsprechenden Datenschutzbestimmungen verwendet. Eine Zuordnung zu Ihrer Person darf nur zu gesetzlich vorgesehenen Zwecken erfolgen oder wenn Sie, nach Aufklärung, eingewilligt haben.

Zuständige und zur Verschwiegenheit verpflichtete Mitarbeiter des Initiators der Studie oder von ihm zum Zweck der wissenschaftlichen Auswertung beauftragte Unternehmen (Näheres dazu in der datenschutzrechtlichen Einwilligungserklärung) können, auch nachdem alle relevanten Daten bereits übermittelt wurden, Einsicht in die beim Studienzentrum vorhandenen Behandlungsunterlagen nehmen, um die Datenübertragung zu überprüfen. Durch Ihre Unterschrift entbinden Sie zu diesem Zweck Ihre Ärzte von der ärztlichen Schweigepflicht. Sofern zur Einsichtnahme autorisierte Personen nicht der obengenannten beruflichen Schweigepflicht unterliegen, stellen personenbezogene Daten, von denen sie bei der Kontrolle Kenntnis erlangen, Betriebsgeheimnisse dar, die geheim zu halten sind.

Verantwortlicher für die Datenverarbeitung gem. Art. 4 Abs. 7 DSGVO ist das Universitätsklinikum Ulm für die Universität Ulm, Medizinische Fakultät (Universitätsklinikum Ulm, 89070 Ulm, info.allgemein@uniklinik-ulm.de). Verantwortlicher für die Datenverarbeitung in der Studie intern ist Herr Professor Dr. Wolfgang Janni, Direktor der Frauenklinik, Tel: 0731-500-58500, E-Mail: direktion.frauenklinik@uniklinik-ulm.de. Bei Fragen zur Nutzung oder Verarbeitung Ihrer Daten wenden Sie sich bitten an ihn oder die oben genannten Studienärzte.

Die Daten werden bis zu 25 Jahre nach Beendigung oder Abbruch der Studie pseudonymisiert aufbewahrt (insgesamt also maximal 35 Jahre). Sie sind gegen unbefugten Zugriff gesichert. Danach werden sie gelöscht.

Die Verwendung dieser studienbezogenen Daten erfolgt nach gesetzlichen Bestimmungen. Rechtsgrundlage für die Verarbeitung sind Ihre freiwillige Einwilligung gemäß Art. 6 Abs. 1 lit. a und Art. 9 Abs. 2 lit. a Datenschutzgrundverordnung (DSGVO).

Sind mit der Datenverarbeitung Risiken verbunden?

Bei jeder Erhebung, Speicherung, Nutzung und Übermittlung von Daten bestehen Vertraulichkeitsrisiken (z. B. die Möglichkeit, die betreffende Person zu identifizieren). Diese Risiken lassen sich nicht völlig ausschließen und steigen, je mehr Daten miteinander verknüpft werden können. Der Initiator der Studie versichert Ihnen, alles nach dem Stand der Technik Mögliche zum Schutz Ihrer Privatsphäre zu tun und Daten nur an Stellen weiterzugeben, die ein geeignetes Datenschutzkonzept vorweisen können. Medizinische Risiken sind mit der Datenverarbeitung nicht verbunden.

Diese pseudonymisierten Daten können auch in Länder außerhalb des EU-Binnenraumes weitergegeben werden, z. B. aber nicht ausschließlich nach England, die Schweiz oder die Vereinigten Staaten von Amerika (USA). In diesen Ländern besteht möglicherweise ein geringeres Datenschutzniveau, dadurch kann es zu Einschränkungen bei der Durchsetzung Ihrer Rechte (z. B. Datenauskunft) geben. Der Initiator der Studie trifft mit dem Empfänger der Daten selbst vertragliche Regelungen, in denen dieser gewährleistet, das EU-Datenschutz-Niveau, soweit rechtlich möglich, einzuhalten. Mit Ihrer Einwilligung stimmen Sie zu, dass die Daten auch in diese Länder übermittelt werden dürfen.

Beachten Sie: Ihre Daten werden nur in pseudonymisierter Form weitergegeben. Der Code (das Pseudonym) kann nur innerhalb der EU in Ihrem Studienzentren entschlüsselt werden, um die pseudonymisierten Daten Ihnen zuzuordnen (siehe oben).

Ihre Rechte:

Sie haben das Recht, vom Verantwortlichen (siehe unten) Auskunft über die von Ihnen gespeicherten personenbezogenen Daten anzufordern, sowie die Überlassung einer kostenlosen Kopie der gespeicherten Daten zu verlangen. Ein Widerruf der Einverständniserklärung und damit eine Löschung der Daten im Rahmen der hier beschriebenen wissenschaftlichen Auswertung ist so lange möglich wie die Zuordnungsmöglichkeit von Studiennummer zu Person existiert (25 Jahre nach Abschluss der Nachbeobachtung, also insgesamt 35 Jahre). Weiterhin haben Sie die folgenden Rechte: Auskunft (Art. 15 DSGVO und §34 BDSG), Widerspruch (Art. 21 DSGVO und §36 BDSG), Datenübertragbarkeit (Art. 20 DSGVO), Löschung (Art. 17 DSGVO und §35 BDSG), Einschränkung der Verarbeitung (Art. 18 DSGVO) und Berichtigung (Art. 16 DSGVO). Nachdem Ihre Daten anonymisiert wurden, ist eine Löschung der Daten nicht mehr möglich, da diese Daten nicht mehr personenbeziehbar sind.

Für die Ausübung Ihrer Rechte wenden Sie sich bitte im Regelfall an das Prüfzentrum, da dieses zuordnen kann, welche Daten zu Ihrer Person gehören.

Bei Anliegen zur Datenverarbeitung und zur Einhaltung der datenschutzrechtlichen Anforderungen können Sie sich auch an folgende Datenschutzbeauftragte wenden:

a) Datenschutzbeauftragter des Studienzentrums: Studienzentrale Universitätsfrauenklinik Ulm, Prittwitzstraße 43, 89075 Ulm, Tel: 0731-500-58652, E-Mail: studienzentrale.ufk@uniklinik-ulm.de

b) Datenschutzbeauftragter des Initiators der Studie: Universitätsklinikum Ulm, Datenschutzbeauftragter, 89070 Ulm, Datenschutz@uniklinik-ulm.de oder Brief mit dem Zusatz „Datenschutzbeauftragter“ an das Universitätsklinikum Ulm, Albert-Einstein-Allee 29, 89081 Ulm.

Sie haben außerdem das Recht sich bei der zuständigen Aufsichtsbehörde für den Datenschutz zu beschweren (Landesbeauftragter für den Datenschutz und die Informationsfreiheit in Baden-Württemberg, Postfach 10 29 32, 70025 Stuttgart, Tel.: 0711 / 61 55 41 - 716, Mail: Poststelle@lfdi.bwl.de).

EINWILLIGUNGSERKLÄRUNG

SURVIVE (Standard Nachsorge im Gegensatz zu einer intensivierten Nachsorge bei PatientInnen mit früher Brustkrebserkrankung) – eine teilweise doppel-blinde, multizentrische, randomisierte, kontrollierte Überlegenheitsstudie

Name des Patienten in Druckbuchstaben: ....

- Ich bin von Herrn / Frau ______________________ über Wesen, Bedeutung und Tragweite der Studie sowie die sich für mich daraus ergebenden Anforderungen aufgeklärt worden. Ich habe darüber hinaus den Text der Patientenaufklärung und dieser Einwilligungserklärung gelesen.
- Ich hatte ausreichend Zeit, Fragen zu stellen und mich zu entscheiden. Aufgetretene Fragen wurden mir vom Studienarzt beantwortet.

Ich hatte zusätzliche Fragen:

....

....

- Ich weiß, dass ich meine freiwillige Mitwirkung jederzeit beenden kann, ohne dass mir daraus Nachteile entstehen.

Ich erkläre mich bereit, an der Studie teilzunehmen.

**Einwilligungserklärung zur Verarbeitung Ihrer personenbezogenen Daten**

1. Ich willige ein, dass personenbezogene Daten über mich, insbesondere zu ethnischer Herkunft, meinen Gesundheitsdaten (Krankengeschichte, Medikamenteneinnahme, sonstige medizinische Befunde) und genetische Befunde und Daten wie in der Informationsschrift beschrieben erhoben und in Papierform sowie auf elektronischen Datenträgern im Institut für Frauengesundheit (IFG) GmbH Erlangen aufgezeichnet werden. Zu diesem Zweck entbinde ich die mich behandelnden Ärzte von der ärztlichen Schweigepflicht.
   Soweit erforderlich, dürfen die erhobenen Daten pseudonymisiert (verschlüsselt) weitergegeben werden an die Universitätsfrauenklinik Ulm, Prittwitzstraße 43, 89075 Ulm, Deutschland und/oder von diesem beauftragte Stellen zum Zweck der wissenschaftlichen Auswertung, auch in Länder außerhalb des EU-Binnenraumes.

2. Ich bin darüber aufgeklärt worden, dass meine Daten auch in Drittländer und an Empfänger weitergegeben werden, für die kein Angemessenheitsbeschluss der Europäischen Kommission und auch keine anderen, gleichwertigen Datenschutzgarantien vorliegen. Ich bin darüber aufgeklärt worden, dass ich ohne meine Einwilligung in die Weitergabe meiner Daten in diese Länder nicht an dieser klinischen Prüfung teilnehmen kann.

3. Außerdem willige ich ein, dass autorisierte und zur Verschwiegenheit verpflichtete Beauftragte des Initiators der Studie Einsicht in die Behandlungsunterlagen bei meinem behandelnden Arzt nehmen, soweit dies zur Überprüfung der Datenübertragung erforderlich ist. Für diese Maßnahme entbinde ich die jeweiligen Ärzte von der Schweigepflicht.

4. Ich bin darüber aufgeklärt worden, dass ich meine Einwilligung jederzeit widerrufen kann. Im Falle des Widerrufs werden keine weiteren Daten mehr erhoben. Ich kann – entsprechend meinen Vorgaben – entweder die Löschung meiner Daten verlangen oder der weiteren Nutzung der bereits erhobenen Daten unter Pseudonym zustimmen.

5. Ich willige ein, dass die Daten nach Beendigung oder Abbruch der klinischen Prüfung 25 Jahre pseudonymisiert aufbewahrt werden. Im Anschluss werden alle personenbezogenen Daten gelöscht.

6. Ich bin nach einem eventuellen Widerruf meiner Einwilligung zur Studienteilnahme mit der Weiterverwendung der bis dahin erhobenen Daten in pseudonymisierter Form einverstanden:

   Ja ☐ Nein ☐

7. Ich willige ein, dass mein Frauenarzt über meine Teilnahme der klinischen Prüfung informiert werden darf.

Ja ☐ Nein ☐

Name Frauenarzt: ____________________

8. Ich willige ein, dass mein Hausarzt über meine Teilnahme der klinischen Prüfung informiert werden darf.

Ja ☐ Nein ☐

Name Hausarzt: ____________________

9. Ich willige ein, dass Gesundheitsdaten von mitbehandelnden Ärzten/ folgenden Ärzten erhoben werden. Insoweit entbinde ich diese Ärzte von der Schweigepflicht.

Ja ☐ Nein ☐

Falls divergierend zu 7. Und 8.: Name/ Funktion: ____________________

10. Ich willige ein, dass mein Biomaterial gemäß den Angaben der Informationsschrift verwendet und weitergegeben wird. Zudem willige ich ein, dass personenbezogene Daten anonymisiert an entsprechende Einrichtungen/Dritte weitergeleitet werden dürfen, auch in Länder außerhalb des EU-Binnenraumes.

11. Ich willige ein, dass meine **Biomaterialien** gemäß den Angaben der Informationsschrift aufbewahrt, verwendet und weitergegeben werden. Ich bin darüber aufgeklärt worden, dass meine Biomaterialien auch in Drittländer und an Empfänger weitergegeben werden, für die kein Angemessenheitsbeschluss der Europäischen Kommission und auch keine anderen, gleichwertigen Datenschutzgarantien vorliegen. Ich bin darüber aufgeklärt worden, dass ich ohne meine Einwilligung in die Weitergabe meiner Biomaterialien in diese Länder nicht an dieser klinischen Prüfung teilnehmen kann.

12. Sollten bei der Analyse der Biomaterialien relevante Ergebnisse festgestellt werden, so willige ich ein, dass ich eventuell zu einem späteren Zeitpunkt erneut kontaktiert werde zum Zweck der Rückmeldung für mich wichtiger gesundheitsrelevanter Ergebnisse:

Ja ☐ Nein ☐

13. Ich willige ein, dass im Falle von einer Neudiagnose einer malignen Erkrankung, die im Rahmen der Untersuchungen dieser Studie nachgewiesen werden, dem Krebsregister gemeldet werden dürfen.

14. Ich stimme einem Datenabgleich mit den Krebsregistern und OnkoZert zu.

Kontaktdaten des behandelnden Arztes:

____
Name und Vorname in Druckschrift

____
Adresse

____
Telefon/Fax/Email

Ich willige in die Verarbeitung der genannten Daten ein.

Ein Exemplar der Informationsschrift und der Einwilligungserklärung habe ich erhalten. Ein Exemplar verbleibt im Prüfzentrum.

Unterschrift des Teilnehmers/der Teilnehmerin

____
Name und Vorname in Druckschrift

____
Datum und Unterschrift

Erklärung und Unterschrift des aufklärenden Arztes/der aufklärenden Ärztin

Ich habe das Aufklärungsgespräch geführt und die Einwilligung eingeholt.

____
Name und Vorname in Druckschrift

____
Datum und Unterschrift

EINWILLIGUNG IN DIE ÜBEREIGNUNG VON KÖRPERMATERIAL ZU

WISSENSCHAFTLICHEN ZWECKEN

Zwischen dem **Universitätsklinikum Ulm**, handelnd für die Abteilung **Klinik für Frauenheilkunde und Geburtshilfe,** Prittwitzstraße 43, 89075 Ulm, vertreten durch Herrn Prof. Dr. Wolfgang Janni und

Frau/Herr____(Name, Vorname, Geburtsdatum)

wird folgendes vereinbart:

Frau/Herr ____

wird dem Universitätsklinikum Ulm das Eigentum an folgendem Körpermaterial unentgeltlich übertragen:

- **Venenblut und Plasma (insgesamt ca. 30 ml Blut pro Blutprobenentnahme, maximal bis zu 60 ml Blut zu Einzelzeitpunkten) zur Biomarker Analyse: bei Studieneinschluss, in den Jahren 1-3 alle 3 Monate, in den folgenden 2 Jahren alle 6 Monate und im Falle eines Rezidivs**

- **Gewebeproben, die aus bei Ihnen bereits durchgeführten diagnostischen Verfahren verfügbar sind (z. B. Biopsien, Operationen). Hierfür ist keine zusätzliche Gewebeentnahme notwendig**

Die Gewebeproben sind für die Herstellung des Bluttests unabdingbar und daher von großer Bedeutung für die SURVIVE-Studie. Sollte nur wenig Tumormaterial vorhanden sein, zum Beispiel weil Ihr Tumor bei einer Chemotherapie gut angesprochen hat, so ist es eventuell notwendig, alles verfügbare Material für die Herstellung der Bluttests zu verwenden. Auch dann kann die Gesamtmenge an Tumormaterial zu wenig sein, so dass der Bluttest und somit die SURVIVE-Studie nicht durchgeführt werden kann.
Die Blutproben werden teils in externen Laboren verarbeitet und gelagert, teils im Labor der Sektion für Gynäkologische Onkologie der Klinik für Frauenheilkunde und Geburtshilfe, Universitätsklinikum Ulm, (Leitung: Frau Prof. Wiesmüller). Das Ziel ist die Identifizierung von Tumormarkern (CA27.29, CEA, CA125), zirkulierenden Tumorzellen (CTC) und zirkulierender Tumor-DNA (ctDNA) um eine eventuelle erneute Erkrankungsmanifestation möglichst frühzeitig zu erkennen. Die Blutanalysen dienen dazu, weiterführende Untersuchung mittels Bildgebungsverfahren (CT Thorax/Abdomen, Knochenszintigraphie) zur Bestätigung bzw. zum Ausschluss einer erneuten Erkrankungsmanifestation in die Wege leiten zu können, sollten sich in den Blutproben Auffälligkeiten ergeben. Blutproben für dieses Forschungsprogramm werden zu Beginn der Studie, in den Jahren 1-3 alle 3 Monate, und in den folgenden 2 Jahren alle 6 Monate gesammelt. Unter Umständen sind bei nicht eindeutigen Ergebnissen weitere Blutentnahmen zur Kontrolle notwendig. Die Untersuchungen der Blutproben und des Tumorgewebes werden zum Teil auch in Zusammenarbeit mit Industriepartnern durchgeführt. Weiterführende Analysen können Nukleinsäure- (Tumor DNA, Keimbahn DNA) und Eiweißuntersuchungen (Proteine) umfassen, müssen aber nicht darauf beschränkt bleiben.

Informationen zu Ihrer Person werden nur in pseudonymisierter Form gespeichert. Die Untersuchungen werden nach Studieneinschluss begonnen und auffällige Ergebnisse dem Arzt der Patientin / des Patienten gemeldet. Forschungsergebnisse können anonymisiert durch Veröffentlichung und/oder Präsentationen auf wissenschaftlichen Tagungen berichtet werden. Für Forschungsergebnisse, welche aus den o.g. Analysen entstehen, wird das Universitätsklinikum Ulm, soweit rechtlich möglich, Schutzrechte begründen und industriellen Kooperationspartnern hieran Nutzungsrechte einräumen. Weitere datenschutzrechtliche Informationen erhalten Sie in der Aufklärung zur Studienteilnahme.

Sie haben jederzeit - auch nachträglich - das Recht, Ihr Einverständnis zu widerrufen und den Übereignungsvertrag

ganz oder teilweise zu kündigen. Hieraus werden für Sie keine Nachteile entstehen. Wenn Sie es wünschen, werden Ihre bisher asservierten Biomaterialien dann vernichtet.

Die Proben werden für maximal 25 Jahre nach Ende der Studie (also maximal 35 Jahre) aufbewahrt und anschließend vernichtet. Mit der EDV-mäßigen Speicherung und Verarbeitung der erhobenen Daten bin ich einverstanden.

Für den Fall, dass alles eingelagerte Tumormaterial für die im Rahmen der SURVIVE-Studie durchgeführten Untersuchungen aufgebraucht werden müsste, stimme ich diesem Vorgehen zu:

Ja ☐ Nein ☐

____

Name und Vorname **PatientIn** in Druckschrift

____

Datum und Unterschrift

____

Name und Vorname **ÄrztIn** in Druckschrift

____

Datum und Unterschrift
