# Supplementary material for: The SURVIVE study (NCT05658172): Bringing breast cancer aftercare to the 21stcentury: Study protocol of a Phase III clinical trial comparing liquid biopsy guided vs. Standard of care surveillance for intermediate to high-risk breast cancer survivors
Source: PLoS One. 2025 Sep 9;20(9):e0331203. doi: 10.1371/journal.pone.0331203 (PMC12419582; doi:10.1371/journal.pone.0331203)
Supplement: S3 File — (PDF) [file pone.0331203.s004.pdf]

SURVIVE (Standard Surveillance vs. Intensive Surveillance in Early Breast Cancer) – a partially double-blinded, multi-center, randomized, controlled superiority study

SURVIVE
Studie

**Protocol Version:**	**2.0**
**Protocol Version Date:**	**02. März 2023**
**BMBF Funding code:**	**01KD2202**
**ClinicalTrials.gov ID:**	**NCT05658172**
**DRKS-ID:**	**DRKS00030745**

VERSION HISTORY

Version 1.0, 14.09.2022
Primary submission approval: approved Ethics Review

Version 2.0, 02.03.2023
Most important changes:

- Inclusion of patients possible immediately after completion of primary therapy
- Modification of study visit names
- No confirmatory blood sampling after first tumor marker increase
- Inclusion of luminal breast cancer patients (ER/PGR positive, HER2 negative) with completed primary therapy within last 5 years
- QoL questionnaires handed out on paper
- Removal of staging examination at inclusion
- Addition of study visit table
- Addition of insurance details
- Addition of german synopsis
- Update of figures

1 ADDRESSES AND RESPONSIBILITIES

Sponsor
Universitätsklinikum Ulm
(Germany)

Sponsor Representative
Prof. Dr. Wolfgang Janni
University Hospital Ulm
Dpt. Obstetrics & Gynecology

Study Directors
Dr. Sophia Huesmann, Prof. Dr. Wolfgang Janni
University Hospital Ulm
Dpt. Obstetrics & Gynecology

Study Office
Dpt. Obstetrics & Gynecology

Protocol Officer
Dr. Sophia Huesmann

Project Management, Monitoring and Randomization
Institut für Frauengesundheit (IFG) GmbH Erlangen

ClinSol GmbH & Co. KG; Clinical Research Solutions

Würzburg
Germany

Statistics

PD Dr. Thomas Friedl, Universitätsklinikum Ulm
Dpt. Obstetrics & Gynecology

2 PROTOCOL BOARD

Prof. Dr.	Wolfgang	Janni	Ulm
Prof. Dr.	Brigitte	Rack	Ulm
Prof. Dr.	Andreas	Hartkopf	Ulm
Prof. Dr.	Peter A.	Fasching	Erlangen
Prof. Dr.	Elisabeth	Wiesmüller	Ulm
PD Dr.	Thomas W. P.	Friedl	Ulm
Dr.	Sophia T.	Huesmann	Ulm
Dr.	Angelina	Fink	Ulm
Dr.	Tatjana	Braun	Ulm
	Forca	Mehmeti	Ulm
	Natalie	Uhl	Ulm

3 STEERING COMMITTEE

Prof. Dr.	Ute-Susann	Albert	Würzburg
Prof. Dr.	Dirk	Bauerschlag	Kiel
Prof. Dr.	Jens-Uwe	Blohmer	Berlin
Prof. Dr.	Wilfried	Budach	Düsseldorf
Prof. Dr.	Peter	Dall	Lüneburg
Prof. Dr.	Nina	Ditsch	Augsburg
Prof. Dr.	Matthias W.	Beckmann	Erlangen
Prof. Dr.	Michael	Friedrich	Krefeld
Prof. Dr.	Bernd	Gerber	Rostock
Prof. Dr.	Kristin	Härtl	München
Prof. Dr.	Nadja	Harbeck	München
Prof. Dr.	Jörg	Heil	Heidelberg
Prof. Dr.	Jens	Huober	St. Gallen
Prof. Dr.	Christian	Jackisch	Offenburg
Prof. Dr.	Cornelia	Kolberg-Liedtke	Essen
Prof. Dr.	Sherko	Kümmel	Essen
Prof. Dr.	Thorsten	Kühn	Esslingen
Prof. Dr.	Sibylle	Loibl	Neu-Isenburg
Prof. Dr.	Diana	Lüfter	Berlin
Prof. Dr.	Michael Patrick	Lux	Paderborn
Prof. Dr.	Nicolai	Maass	Kiel
Prof. Dr.	Christoph	Mundhenke	Bayreuth
Prof. Dr.	Hans	Neubauer	Düsseldorf
Prof. Dr.	Klaus	Pantel	Hamburg
Prof. Dr.	Tjoung-Won	Park-Simon	Hannover
Prof. Dr.	Toralf	Reimer	Rostock
Prof. Dr.	Kerstin	Rhiem	Köln
Prof. Dr.	Achim	Rody	Lübeck
Prof. Dr.	Marcus	Schmidt	Mainz
Prof. Dr.	Andreas	Schneeweiß	Heidelberg
Prof. Dr.	Florian	Schütz	Speyer
Prof. Dr.	Christine	Solbach	Frankfurt am Main
Prof. Dr.	Erich-Franz	Solomayer	Homburg
Prof. Dr.	Elmar	Stickeler	Aachen
Prof. Dr.	Marc	Thill	Frankfurt a. M.
Prof. Dr.	Christoph	Thomssen	Halle
Prof. Dr.	Michael	Untch	Berlin
Prof. Dr.	Markus	Wallwiener	Heidelberg
Prof. Dr.	Isabell	Witzel	Hamburg
Prof. Dr.	Achim	Wöckel	Würzburg
PD Dr.	Maggie	Banys-Paluchowsky	Lübeck
PD Dr.	Eva Maria	Fallenberg	München
PD Dr.	David	Krug	Kiel

PD Dr.	Oleg	Gluz	Mönchengladbach	
PD Dr.	Hans-Christan	Kolberg	Bottrop	
Dr.	Ingo	Bauerfeind	Landshut	
Dr.	Ralph	Wirtz		Stratifyer
	Renate	Haidinger		Brustkrebs Deutschland
	Sabine	Kirton		Frauenselbsthilfe Krebs
	Gertrud	Rust		mamazone
	Doris	Schmitt		Stiftung PATH
	Eva	Schumacher-Wulf		Mamma Mia! Magazine

4 APPROVAL SIGNATURES

SURVIVE (Standard Surveillance vs. Intensive Surveillance in Early Breast Cancer) – a partially double-blinded, multi-center, randomized, controlled superiority study

Protocol Version: **2.0**
Protocol Version Date: **02.03.2023**

I, the undersigned, have read this protocol and confirm that to the best of my knowledge it accurately describes the planned conduct of the study.

Signature: Date:

____________________________________ ______________________
Dr. Sophia Huesmann, Prof. Dr. Wolfgang Janni
Study Chairs

____________________________________ ______________________
Dr. Udo X. Kaisers
Chief Medical Director University Hospital Ulm
Representing the sponsor

____________________________________ ______________________
Bettina Rottke
Commercial Director University Hospital Ulm
Representing the sponsor

____________________________________ ______________________
ZKS Ulm

____________________________________ ______________________
PD Dr. Thomas Friedl
Study Statistician

5 PROTOCOL SYNOPSIS

**Study Title**	SURVIVE (Standard Surveillance vs. Intensive Surveillance in Early Breast Cancer)
**Sponsor**	University Hospital Ulm
**Rationale**	Breast cancer is the world's most common cancer entity with a median age of 64 at first diagnosis, affecting all age groups after puberty. While the incidence of breast cancer increased over the last decades, treatment options for breast cancer patients continue to improve, providing a growing range of therapeutic agents.<br>After patients with early breast cancer have completed primary therapy, guidelines limit routine surveillance in breast cancer survivors to clinical surveillance and breast imaging. Screening for distant metastases is initiated only in patients with specific symptoms. This approach is based on two large cohort studies performed in the 1980s suggesting no superiority of intensified screening for distant metastases versus standard surveillance for overall survival (OS). However, patients frequently feel left-alone during this stage of their disease. Furthermore, diagnostic and therapeutic options for the treatment of early (oligo-) metastatic disease have dramatically improved over the past decades (e. g., HER2-neu targeted treatment, endocrine combination treatment with CDK4/6-inhibitors, cytostatic conjugates, PARP-inhibitors, stereotactic radiotherapy, etc.). We hypothesize, in consideration of modern therapeutic agents, that earlier detection of distant (oligo-) metastases and therefore earlier initiation of therapy in a pre-symptomatic stage leads to improved overall survival (OS) today. We suggest the evaluation of a liquid biopsy-guided follow-up surveillance method for the detection of distant (oligo-) metastases before the onset of symptoms, analyzing the tumor markers mucin-1 (MUC1)- based cancer antigen 27.29 (CA27.29), mucin-16 (MUC16)- based CA125 and carcinoembryonic antigen (CEA) serum levels as well as circulating tumor cells (CTCs) and circulating tumor DNA (ctDNA) in a multicenter, prospective, partially double-blinded, controlled randomized study for medium- and high-risk early breast cancer patients. This study could contribute to a paradigm shift in the current follow-up care of medium and high-risk early breast cancer survivors.
**Study Overview**	This is a partially double-blinded, multi-center, randomized, controlled superiority study to evaluate the potential benefits of intensified surveillance versus standard surveillance in medium-risk and high-risk early breast cancer patients.<br>3500 patients will be randomized in a 1:1 ratio after completion of primary anti-tumor therapy (adjuvant chemotherapy, surgery or radiotherapy, whichever occurs last) to receive:<br>- **Standard Surveillance** according to national guidelines or<br>- **Intensive Surveillance** with additional testing of blood samples for prospective tumor markers (CA27.29, CA125, CEA), CTC and ctDNA

	In both study arms patients will continue to receive standard surveillance according to national guidelines, including clinical follow-up visits every 3 months for the first 3 years and every 6 months for the following 2 years. Additionally, blood samples will be drawn and Quality of Life (QoL) will be analyzed at these clinical follow-up visits in both arms.<br>In the **Standard Surveillance** arm blood samples will be stored in a biobank.<br>In the **Intensive Surveillance** arm blood samples will be tested for prospective tumor markers (CA27.29, CA125, CEA), CTCs and ctDNA. Abnormal findings of either marker (CA27.29 or CA125 or CEA or CTC or ctDNA) will trigger diagnostic imaging. Additionally, blood samples will be stored in a biobank for retrospective analysis.<br>In both study arms detection of distant recurrence via imaging will terminate the surveillance protocol and treatment will be initiated according to national guidelines.<br>Planned enrollment period is approximately 24 months, total study duration is approximately 144 months (2-year recruitment period, 5-year interventional period, 5-year follow up period). In terms of long-term follow-up after end of study, patients have the possibility to participate in the patient self-reporting registry (Patientenselbstauskunft).
**Intervention**	All participating patients with no clinical evidence of disease after primary anti-tumor therapy (adjuvant chemotherapy, surgery or radiotherapy, whichever occurs last) will follow the German AGO-guideline based standard follow-up surveillance in addition to a blood withdrawal of 27,5 mL whole blood (2 x 10 mL Streck Cell-Free DNA BCT®, 1 x 7,5 mL Serum; every 3 months for the first 3 years after enrollment, every 6 months for the consecutive 2 years). In case of M1-detection or local recurrence of disease, the patients will receive the guideline-based diagnostic measures and therapy.<br>Quality of Life (QoL) will be monitored using established questionnaires (EORTC QLQ-C30 and PA-F12) every 6 months. All patients will be asked questions directed to performed examinations (blood or imaging) and symptoms outside the study within the last months since the last visit.<br>Patients will be randomized 1:1 to an intensive versus standard surveillance group. Randomization will be blinded for patients and treatment centers. However, when imaging is triggered by a positive biomarker result in patients in the Intensive Surveillance arm, these patients have to be unblinded.<br><br>**Intensive Surveillance:** Prospective tumor marker (CA27.29, CA125, CEA), CTC and ctDNA testing of the blood samples. Abnormal findings (as defined below) of either marker (CA27.29 and/or CA125 and/or CEA and/or CTC and/or ctDNA) will be regarded as molecular relapse and trigger diagnostic imaging.<br><br>**Tumor marker:** A sample of peripheral blood is taken after randomization for baseline determination (2 blood drawings 28 ± 5 days apart; mean value for CA27.29, CEA and CA125 determines baseline) and thereafter every 3 months for

	3 years, thereafter every 6 months for another 2 years. Patients with an abnormal tumor marker serum level increase from baseline (CA27.29 +75% or CEA +100% or CA125 +150%) will undergo complete standard staging examinations (as a minimum CT scan of the chest and abdomen + SPECT bone scan). In case of M0, the patient will continue liquid-biopsy based tumor marker testing. If tumor marker elevation continues, a maximum of 3 consecutive negative staging examinations will be performed before another rise in tumor marker will be mandatory (according to the deltas stated above) to trigger further imaging. The minimum interval between two CT scans should be at least 3 months, and SPECT bone scans should be at least 6 months apart at any time point.<br><br>**CTC:** If an abnormal tumor marker value is detected for the first time, additional blood for CTC-analysis will be collected at the next regular study visit. Furthermore, all patients of the experimental arm will receive CTC-testing 1 year after study enrollment. If ≥ 1 CTC is detected (irrespective of whether there is a confirmed abnormal tumor marker increase), patients will undergo complete standard staging examinations. In case of M0, the patient will continue liquid-biopsy based testing in the same intervals as before (every 3 or 6 months). The minimum interval between two CT scans should be at least 3 months, and SPECT bone scans should be at least 6 months apart at any time point.<br><br>**ctDNA:** Presence of ctDNA will be measured every 3 months for 3 years, thereafter every 6 months for another 2 years. If ctDNA is detected, patients will undergo complete standard staging examinations. In case of M0, the patient will continue liquid-biopsy based ctDNA testing. In case of persisting ctDNA, a maximum of 3 consecutive negative staging examinations will be performed before staging intervals will be extended to every 6 months for another 3 consecutive scans. If ctDNA is still persisting and patients show no radiological sign of recurrence, staging intervals will be extended to once a year. The minimum interval between two CT scans should be at least 3 months, and SPECT bone scans should be at least 6 months at any time point.<br><br>**Standard Surveillance:** Blood samples will not be analyzed immediately and will therefore not trigger imaging. A biobank will be established for retrospective and translational studies. This procedure is necessary to ensure the partially double-blinded study design.<br><br>**Duration of intervention per patient:** 5 years.<br><br>Patients will be randomized 1:1 to the intensive versus standard surveillance group. Randomization will be blinded for patients and treatment centers. However, when imaging is triggered by a positive biomarker result in patients in the Intensive Surveillance arm, these patients have to be unblinded.

**Primary Objectives and Endpoints**	**Two Co-Primary-Endpoints will be investigated:**<br><br>**Overall Survival (OS)**<br><ins>Objective:</ins><br>To compare 5-year OS rates between patients in the **Standard Surveillance** arm versus patients in the liquid-biopsy guided **Intensive Surveillance** arm.<br><ins>Endpoint:</ins><br>OS is defined as time from randomization until the death of the patient independent of cause of death. If a patient is not known to have died, OS is censored at the date of last contact.<br><br>**Overall Lead Time Effect**<br><ins>Objective:</ins><br>To determine the Overall Lead Time Effect generated due to tumor marker/CTC/ctDNA guided **Intensive Surveillance** compared to **Standard Surveillance** after primary therapy in early breast cancer patients.<br><ins>Endpoint:</ins><br>This endpoint is a composite measure, defined as the median time from molecular to via Imaging verified Distant Recurrence Lead Time (calculated only for patients in the liquid-biopsy guided Intensive Surveillance arm; see secondary endpoint definition below) + Difference in time to distant recurrence between the two arms (i.e., difference between median time from randomization to distant recurrence for all patients with distance recurrence in the Standard Surveillance arm and median time from randomization to distant recurrence for all patients with distant recurrence in the liquid-biopsy guided Intensive Surveillance arm; see Figure 2). The Overall Lead Time Effect will be assessed for all markers in combination.
**Secondary Objectives and Endpoints**	**Invasive disease-free survival (IDFS)**<br><ins>Objective:</ins><br>To compare IDFS between patients in the **Standard Surveillance** arm versus patients in the liquid-biopsy guided **Intensive Surveillance** arm.<br><ins>Endpoint:</ins><br>IDFS is defined as time from randomization until first IDFS event, including any invasive ipsilateral, regional, contralateral, and distant disease recurrence, second primary tumors, or death from any cause as event; non-invasive, in-situ cancer events are excluded. If a patient has not had an event, IDFS is censored at the date of last adequate tumor assessment.<br><br>**Distant disease-free survival (DDFS)**<br><ins>Objective:</ins><br>To compare DDFS between patients in the **Standard Surveillance** arm versus patients in the liquid-biopsy guided **Intensive Surveillance** arm.<br><ins>Endpoint:</ins><br>DDFS is defined as time from randomization until first DDFS event including

	metastasis, second primary tumors and death from any cause as event. If a patient has not had an event, DDFS is censored at the date of last adequate tumor assessment.<br><br>**Distant recurrence-free survival (DRFS)**<br><u>Objective:</u><br>To compare DRFS between patients in the **Standard Surveillance** arm versus patients in the liquid-biopsy guided **Intensive Surveillance** arm.<br><u>Endpoint:</u><br>DRFS is defined as time from randomization until first DRFS event including metastasis and second primary tumors; death from any cause is not included as event. If a patient has not had an event, DRFS is censored at the date of last adequate tumor assessment.<br><br>**Breast cancer specific survival (BCSS)**<br><u>Objective:</u><br>To compare BCSS between patients in the **Standard Surveillance** arm versus patients in the liquid-biopsy guided **Intensive Surveillance** arm.<br><u>Endpoint:</u><br>BCSS is defined as time from randomization until breast cancer associated death of the patient. If a patient is not known to have died, BCSS is censored at the date of last contact. If a patient has died for reasons not associated with breast cancer (by clinical assessment), BCSS is censored at the date of death.<br><br>**Invasive breast cancer free survival (IBCFS)**<br><u>Objective:</u><br>To compare IBCFS between patients in the **Standard Surveillance** arm versus patients in the liquid-biopsy guided **Intensive Surveillance** arm.<br><u>Endpoint:</u><br>IBCFS is defined as time from randomization until first IBCFS event, including any invasive ipsilateral, regional, contralateral and distant disease recurrence or death from any cause as event; non-invasive, in-situ cancer events are excluded. If a patient has not had an event, IBCFS is censored at the date of last adequate tumor assessment.<br><br>**Overall Survival (OS) after 10 Years**<br><u>Objective:</u><br>To compare 10-year OS rates between patients in the **Standard Surveillance** arm versus patients in the liquid-biopsy guided **Intensive Surveillance** arm.<br><u>Endpoint:</u><br>OS is defined as time from randomization until the death of the patient independent of cause of death. If a patient is not known to have died, OS is censored at the date of last contact.

	**Molecular to via Imaging verified Distant Recurrence Lead Time in the Interventional arm**<br>Objective:<br>To determine the Lead Time (median, interquartile, range, 95% confidence interval) generated in the liquid-biopsy guided **Intensive Surveillance** arm.<br>Endpoint:<br>The Lead Time is defined as time from first molecular relapse (definitions see above and in Figure 2) to via imaging verified distant recurrence for all patients in the liquid-biopsy guided Intensive Surveillance arm for whom a via imaging verified distant recurrence is documented during the 5-year interventional period of the study. If a patient has a via imaging verified distant recurrence but no documented molecular relapse, the Lead Time for this patient is set to '0'. This secondary endpoint will be assessed for all markers in combination and, where applicable, for each marker separately.<br><br>**Quality of life (QoL) with questionnaires: EORTC QLQ-C30 and PA-F12**<br>Objective:<br>To compare QoL between patients in the **Standard Surveillance** arm versus patients in the liquid-biopsy guided **Intensive Surveillance** arm.<br>Endpoint:<br>QoL will be monitored in both groups using 2 questionnaires which are to be completed every 6 months (EORTC QLQ-C30 and PA-F12) in the interventional phase (first five years) and once a year during the five years of follow-up. QoL data will be collected on paper or via a digital health application.<br><br>**Liquid biopsy sensitivity (CA27.29, CEA, CA125, CTC and ctDNA)**<br>Objective:<br>To determine sensitivity (i.e., the true positive rate) of the liquid biopsy markers in combination (and individually where applicable) in the liquid-biopsy guided **Intensive Surveillance** arm.<br>Endpoint:<br>Sensitivity is defined as the proportion of all patients in the liquid-biopsy guided Intensive Surveillance arm with a distant recurrence as verified by imaging during the 5-year interventional period of the study that had a positive biomarker result (i.e., molecular relapse) within 36 months before the recurrence as verified by imaging occurred (or within 60 months before the recurrence as verified by imaging occurred if in the first 36 months at least once a year a positive biomarker is measured).<br><br>**Liquid biopsy specificity (CA27.29, CEA, CA125, CTC and ctDNA)**<br>Objective:<br>To determine specificity (i.e., the true negative rate) of the liquid biopsy markers in combination (and individually where applicable) in the liquid-biopsy guided **Intensive Surveillance** arm.

	<u>Endpoint:</u><br>Specificity is defined as the proportion of all patients in the liquid-biopsy guided Intensive Surveillance arm with no recurrence as verified by imaging during the 5-year interventional period of the study that had only negative biomarker results (i.e., no indication of molecular relapse) within 36 months before the end of the 5-year interventional period of the study.<br><br>**Liquid Biopsy False-Positive Rate (CA27.29, CEA, CA125, CTC and ctDNA)**<br><u>Objective:</u><br>To determine the false-positive rate of the liquid biopsy markers in combination (and individually where applicable) in the liquid-biopsy guided **Intensive Surveillance** arm.<br><u>Endpoint:</u><br>False-positive rate is defined as the proportion of all patients in the liquid-biopsy guided Intensive Surveillance arm with a positive biomarker result (i.e., molecular relapse) during the 5-year interventional period of the study that had no recurrence as verified by imaging within 36 months after first molecular relapse.<br><br>**Liquid Biopsy False-Negative Rate (CA27.29, CEA, CA125, CTC and ctDNA)**<br><u>Objective:</u><br>To determine the false-negative rate of the liquid biopsy markers in combination (and individually where applicable) in the liquid-biopsy guided **Intensive Surveillance** arm.<br><u>Endpoint:</u><br>False-negative rate is defined as the proportion of all patients in the liquid-biopsy guided Intensive Surveillance arm with only negative biomarker results (i.e., no indication of molecular relapse) that had a recurrence as verified by imaging during the 5-year interventional period of the study.<br><br>**Rate of liquid biopsy positivity (CA27.29, CEA, CA125, CTC and ctDNA)**<br><u>Objective:</u><br>To determine the overall rate of positive liquid biopsy marker results in combination (and individually where applicable) in the liquid-biopsy guided **Intensive Surveillance** arm.<br><u>Endpoint:</u><br>The overall rate of liquid biopsy positivity is defined as the proportion of all patients in the liquid-biopsy guided Intensive Surveillance arm that had at least one positive biomarker result (i.e., molecular relapse) during the 5-year interventional period of the study.
**Translational Research Objectives and Endpoints**	<u>Objective:</u><br>To ensure the possibility of retrospective studies during and after the ongoing study, a biobank will be implemented.

	<u>Endpoint:</u><br>A biobank will allow the storage of unused biomaterials from both, the Intensive Surveillance arm as well as the Standard Surveillance arm. These materials will be used for future retrospective studies.
**Inclusion Criteria**	Patients will be eligible for study participation only if they comply with the following criteria:<br>1. Written informed consent for all study procedures according to local regulatory requirements prior to beginning specific protocol procedures.<br>2. Unilateral or bilateral primary invasive carcinoma of the breast, confirmed histologically.<br>3. Patients with intermediate- to high-risk early breast cancer defined as either<br>- an indication for (neo-)adjuvant chemotherapy (regardless whether performed or not), and/or<br>- Large tumor (> 50 mm), and/or<br>- Positive lymph nodes (> pN1mi), and/or<br>- High grade (≥ G3).<br>4. A complete resection of the primary tumor, with resection margins free of invasive carcinoma.<br>5. Completion of primary anti-tumor therapy (adjuvant chemotherapy, surgery or radiotherapy, whichever occurs last) no more than 24 months previously. Enrollment of patients during any kind of adjuvant therapy except chemotherapy (e.g., but not limited to endocrine therapy, antibody therapy, CDK4/6-inhibitors, PARP inhibitors, PI3K inhibitors, antibody-drug conjugates and other novel agents) is allowed. Patients with Luminal A/B breast cancer (ER/PGR positive, HER2 negative/low) may be enrolled up to 60 months after completion of primary anti-tumor therapy.<br>6. Availability of primary tumor tissue from core biopsy or surgical removed tissue (FFPE Slide (≥ 6 mm³, min. 10 slides, thickness: 5 µm-10 µm, area >150 mm² and 1 H&E stained slide, minimum 20% tumor content) or FFPE Block (≥ 6 mm³ thickness: 100 µm, area: >150 mm² and 1 H&E stained slide, minimum 20% tumor content) or Genomic DNA extracted from FFPE slides or block (≥ 600 ng, Minimum volume: 25 µL, concentration: 20 ng/µL, buffer: 10 mM Tris pH 8, 1 mM EDTA)) at timepoint of enrollment<br>- Patients with primary systemic therapy: tissue from core biopsy<br>- Patients receiving surgery as primary therapy: surgically removed cancer tissue or tissue from core biopsy.<br>Important note: Tumor tissue of both sides will be needed in case of bilateral breast cancer.<br>7. No current clinical evidence for distant metastases.<br>8. Females or males ≥ 18 years and ≤ 75 years of age.

	9. Performance status ≤ 1, Eastern Cooperative Oncology Group (ECOG) scale.<br>10. Patient must be willing and able to comply with scheduled visits, treatment plans, laboratory tests, and other study procedures.
**Exclusion Criteria**	Patients will be ineligible for study participation if they comply with the following criteria:<br>1. Patients with a history of any secondary primary malignancy are ineligible with the following exceptions:<br>- in situ carcinoma of the cervix or<br>- adequately treated basal cell carcinoma of the skin or<br>- ipsi- or contralateral non-invasive carcinoma of the breast (DCIS).<br>2. Patients in pregnancy or breastfeeding.<br>3. History of significant neurological or psychiatric disorders including psychotic disorders, dementia or seizures that would prohibit the understanding and giving of informed consent.<br>4. Renal insufficiency with GFR < 30 mL/min.<br>5. Previous or concomitant cytotoxic or other systemic antineoplastic treatment that is not used for treating the primary breast cancer.
**Sample Size Determination**	The study is designed as a two-arm parallel, (partially) double-blinded randomized superiority study. To detect a 2.35% improvement in 5-year OS rate from 92.65% in the standard surveillance arm to 95.0% in the tumor marker, ctDNA and CTC guided surveillance arm (equivalent to a hazard ratio of 0.672), approximately 3500 patients (1750 in both arms) will be required to achieve 80% power at a 2-sided significance level of 5%, assuming a uniform accrual pattern (accrual time 2 years), a dropout rate of 15% in both arms and a total study duration of 10 years. The reference value of 92.65% 5-year OS rate is based on the recently published results from the randomized phase III Success A study (Clinicaltrials.gov NCT02181101) that reported 5-year OS rates for 3754 intermediate-to-high-risk early breast cancer patients receiving standard chemotherapy regimen.
**Randomization**	Patients will be randomized centrally using permuted block list only if they fulfil the inclusion criteria and are not precluded from participation by any of the exclusion criteria.
**Stratification Factors**	Randomization will be stratified using block randomization by:<br>• Hormone receptor status (negative vs positive)<br>• HER2 status (negative vs positive)<br>• Histological lymph node status at surgery ((y)pN0 vs (y)pN+)
**Statistical Methods**	Study Populations:<br>The following study population sets will be examined:<br>• Intention to Treat (ITT) Set: All randomized patients with signed informed consent

	• Per Protocol (PP) Set: All patients of the ITT set who did not violate any inclusion or exclusion criterion and who were treated strictly according to protocol.<br>If necessary, further data sets required for additional analyses may be specified in separate analysis plans.<br><br>**Primary endpoint analysis and population:**<br>The primary endpoint overall survival will be analyzed based on the ITT set, estimated by the Kaplan Meier product limit method, and 5-year OS rates, 95% confidence intervals and survival plots will be provided. Overall survival will be compared between patients in the two randomization arms using the log-rank test, and univariable cox regression as well as additional multivariable analyses adjusted for other factors will be performed using suitable regression models (cox proportional hazard regression model). Hazard ratios and the corresponding 95% confidence intervals will be reported as obtained by both univariable and adjusted multivariable analyses.<br>The co-primary endpoint Overall Lead Time Effect will be calculated as described above. As it is a purely descriptive composite measure consisting of two unrelated median values calculated for different patient cohorts, no confidence intervals will be provided.<br><br>**Secondary endpoint analysis:**<br>All analyses regarding the secondary objectives will have exploratory character only. All secondary endpoints and other outcomes that are calculated based on frequencies/rates will be summarized using frequency tables presenting absolute and relative frequencies together with appropriate confidence intervals.<br><br>Comparisons between groups will be performed using suitable non-parametric statistical tests such as Fisher's exact test, $\chi2$-test, or Cochran-Mantel-Haenszel test. Continuous variables will be summarized by descriptive statistics (number of valid and missing observations, mean, standard deviation, median, interquartiles, minimum, and maximum) and compared between arms using suitable non-parametric tests such as Wilcoxon-Mann-Whitney. Secondary survival endpoints (IDFS, DDFS, DRFS, BCSS) will be analyzed in the same way as the primary endpoint OS. Tumor marker (CA27.29, CEA, CA125) levels, presence and number of CTCs as well as presence of ctDNA measured at different time points will be evaluated in a descriptive way, and the temporal changes will be described and analyzed using appropriate statistical models (e.g., generalized linear mixed models). Quality of life data will be analyzed according to the instructions of the validated questionnaires used (EORTC QLQ-C30 and PA-F12). Additional exploratory analyses (e.g., subgroup analyses) will be performed as appropriate.<br>More details regarding the statistical analyses will be provided in a Statistical Analysis Plan (SAP) which will be finalized prior to data base lock.

**Interim Analyses**	No formal efficacy interim analysis is planned.<br><br>A three-step futility analysis for ctDNA is planned (as per request of the contributing company):<br><br>**Step 1:**<br>Analysis of minimum requirement regarding ctDNA positivity rate.<br><br>• Analysis is performed based on the first 150 patients enrolled in the experimental arm to calculate the ctDNA positivity rate.<br>• Analysis is considered to be affirmative to continue ctDNA testing within the study,<br>- if the ctDNA positivity rate measured 3 months after recruitment is > 5% for these 150 patients and<br>- if the ctDNA positivity rate measured 12 months after recruitment is > 10% for these 150 patients.<br><br>**Step 2:**<br>Analysis of minimum requirement regarding molecular to via imaging verified distant recurrence lead time (from the time of ctDNA positive result – i.e., molecular relapse - to the time of image-verified distant relapse; see detailed definition in “Secondary Objectives and Endpoints”)<br><br>• Analysis is performed after 30 patients in the experimental arm had a ctDNA positive result and a via imaging verified distant recurrence (without additional treatment intervention) and a minimum follow-up time of 6 months (as from the first ctDNA-positive result), to calculate the median lead time from the time of the first ctDNA positive result to the time of via imaging verified distant relapse for these 30 patients.<br>• Analysis is considered to be affirmative to continue ctDNA testing within the study, if this lead time is > 6 months.<br><br>**Step 3:**<br>Analysis of minimum requirement regarding overall lead time (molecular relapse to via imaging verified distant recurrence lead time + Difference in median time to distant recurrence between the study arms; see detailed definition in “Primary Objectives and Endpoints” above)<br><br>• Analysis is performed after 50 patients in the experimental arm had a ctDNA positive result and a via imaging verified distant recurrence and a minimum follow-up time of 12 months (as from the first ctDNA positive result), to calculate the overall lead time (molecular relapse to via imaging verified distant recurrence lead time + Difference in median time to distant recurrence between the study arms) for these 50 patients.

	• Analysis is considered to be affirmative to continue ctDNA testing within the study, if this overall lead time is > 9 months.<br><br>If these requirements for continuation of ctDNA testing are not fulfilled, termination of ctDNA testing in this trial might be considered pending decision of the steering board and the company.
**Biomaterial**	- Primary tumor tissue from core biopsy or surgical removed tissue (FFPE Slide (≥ 6 mm³, min. 10 slides, thickness: 5 µm-10 µm, area >150 mm² and 1 H&E stained slide, minimum 20% tumor content) or FFPE Block (≥ 6 mm³ thickness: 100 µm, area: >150 mm² and 1 H&E stained slide, minimum 20% tumor content) or Genomic DNA extracted from FFPE slides or block (≥ 600 ng, Minimum volume: 25 µL, concentration: 20 ng/µL, buffer: 10 mM Tris pH 8, 1 mM EDTA)) at timepoint of enrollment<br>▪ Patients with primary systemic therapy: tissue from core biopsy<br>▪ Patients receiving surgery as primary therapy: surgically removed cancer tissue or tissue from core biopsy.<br>Important note: Tumor tissue of both sides will be needed in case of bilateral breast cancer.<br>- Two Streck Cell-Free DNA BCT® tubes for ctDNA (circulating tumor DNA) analysis at timepoint of enrollment and every 3 months for 3 years, thereafter every 6 months up to 5 years.<br>- One standard serum sampling tube (7,5 mL whole blood) for detection of tumor markers at timepoint of enrollment, 4 weeks after enrollment and every 3 months for 3 years, thereafter every 6 months up to 5 years.<br><br>Additionally, another serum sampling tube will be drawn 2 weeks after significant increase of one of the tumor markers for confirmation.<br>- Three whole blood samples in 10 mL CellSave preservative tubes for detection of CTCs (Circulating Tumor Cells) one year after study inclusion. Patients with detection of a tumor marker rise for the first time will have one additional blood sampling for CTCs (three whole blood samples in 10 mL CellSave preservative tubes) at the next regular study visit. If ≥ 1 CTC is detected and staging is M0, patients will continue liquid biopsy testing as before.<br>Blood samples from patients in the control group will not be analyzed immediately but stored for later retrospective analyses in the context of translational research projects.
**Enrollment period**	Approximately 24 months.
**Follow-up**	After the interventional part of the study is concluded with the last liquid biopsy taken at the end of Year 5, follow-up of five years without any intervention is intended. Follow-up data is collected via digital health application, telephone calls or on paper. The assessments for the follow-up visits are described in the study protocol.<br>In terms of long-term follow-up after end of study, patients have the possibility to participate in the patient self-reporting registry (Patientenselbstauskunft).

**Number of sites**	It is planned to conduct the study with up to 150 study sites in Germany.<br>These study sites have already participated in various studies within the AGO B, SUCCESS and DETECT study groups, and the SURVIVE study is supported by several German breast-cancer patient advocacy groups (mamazone e. V., PATH-Foundation, Brustkrebs Deutschland e. V., Frauenselbsthilfe Krebs e. V., MammaMia!).
**Timelines:**	FPI Q4/2022<br>LPI Q4/2024<br>LP EOT / EOI Q4/2029<br><br>End of Study (EOS) Q4/2035

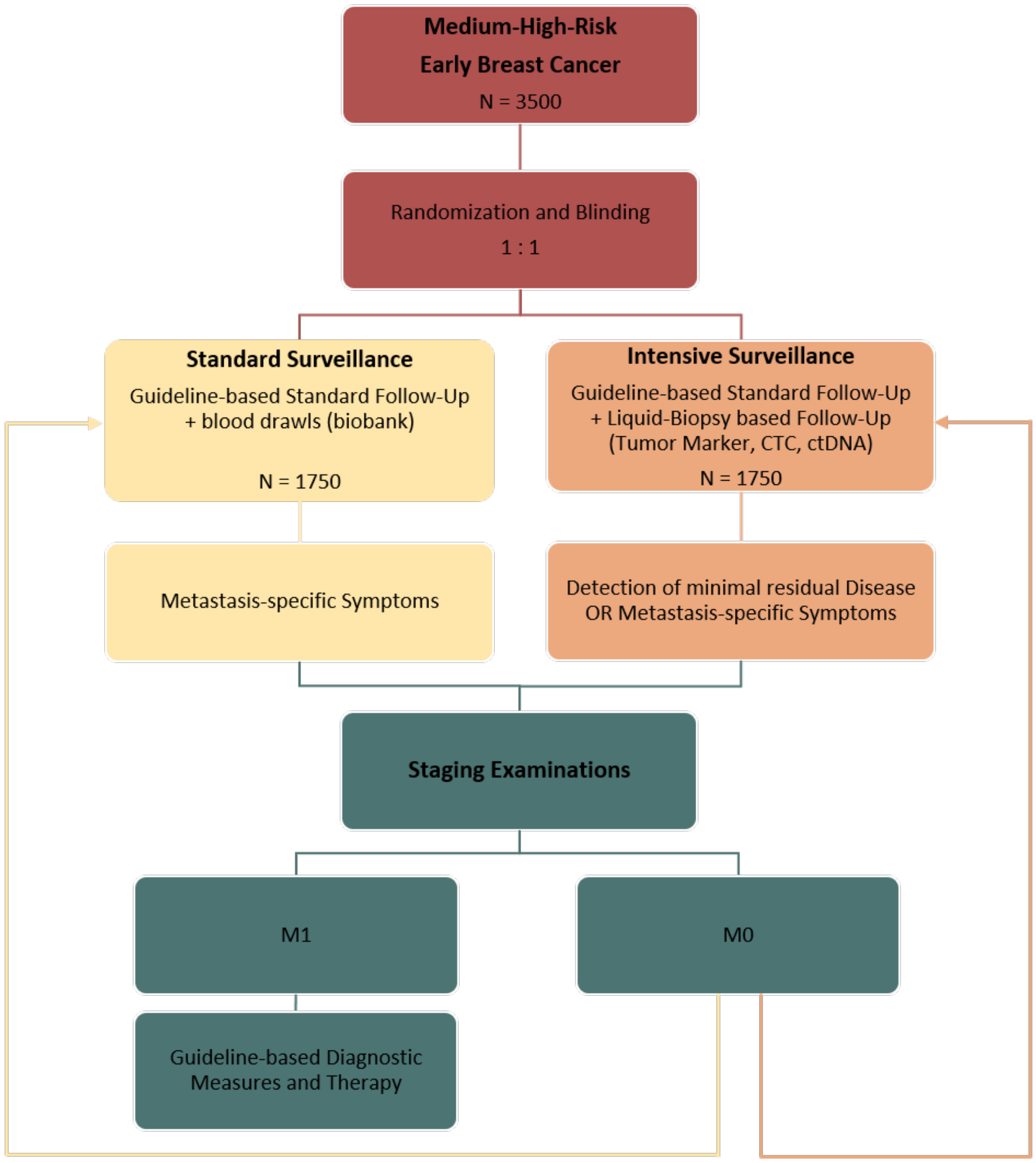

Figure 1: SURVIVE Study Design

6 TABLE OF CONTENTS

1 ADDRESSES AND RESPONSIBILITIES 3
2 PROTOCOL BOARD 5
3 STEERING COMMITTEE 6
4 APPROVAL SIGNATURES 8
5 PROTOCOL SYNOPSIS 9
6 TABLE OF CONTENTS 23
7 INTRODUCTION 27

7.1 Prevalence, Incidence, Mortality of Breast Cancer 27
7.2 Burden of disease 27
7.3 Impact of the Study 27
7.4 Standard Follow-Up Surveillance in Early Breast Cancer Patients 28

7.4.1 Rationale for Tumor markers in Liquid biopsy-based Follow-Up 29
7.4.2 Rationale for CTCs in Liquid biopsy-based Follow-Up 30
7.4.3 Rationale for ctDNA in Liquid biopsy-based Follow-Up 30

7.5 Appropriateness of the Primary Endpoint 31
7.6 Risk – Benefit Analysis for the Participants 32
7.7 Interpretation of Potential Study Results 33

8 STUDY OBJECTIVES 34

8.1 Primary Objectives 34
8.2 Secondary Objectives 34
8.3 Translational Research Objectives 35

9 STUDY ENDPOINTS 36

9.1 Primary Endpoints 36
9.2 Secondary Endpoints 36
9.3 Translational Research Endpoints 38

10 STUDY DESIGN 39

10.1 Description of the Study Design 39
10.2 Study design 40

11 STUDY POPULATION 41

11.1 Number of Patients 41
11.2 Inclusion Criteria 41
11.3 Exclusion Criteria 42

12 STUDY INTERVENTIONS 43

12.1 Standard Surveillance 43
12.2 Intensive Surveillance 44

12.2.1 Determination of Tumor markers 44

12.2.2 Determination of CTC levels 45
12.2.3 Determination of ctDNA levels 46
12.2.4 Overview of Requirements for Collection of Biomaterials 48
12.2.5 Blinding 48

12.3 Staging Examinations 48
12.4 Study visits 48

12.4.1 Study Inclusion / Baseline Visit 49
12.4.2 Visits During Interventional Part of the Study 49
12.4.3 End of Intervention (EOI) 50

12.5 Study Procedures 51

12.5.1 Patient Entry Procedures 54

12.5.1.1 Patient Consent Form and Screening 54
12.5.1.2 Randomization and Stratification 55

12.5.2 Screening 55
12.5.3 Evaluation during Interventional Phase 57
12.5.4 Evaluation after End of Intervention - Follow-up 58
12.5.5 Long-Term Follow-up After End of Study 59
12.5.6 Study visits table 60

13 COLLECTION OF BIOMATERIALS AND TRANSLATIONAL RESEARCH 63

13.1 Overview of Requirements for Collection of Biomaterials 63

13.1.1 Tumor Material 64

13.1.1.1 Mandatory tumor material 64
13.1.1.2 Optional tumor material 64

13.1.2 Blood Samples 64

13.1.2.1 Mandatory blood samples for all patients 64

13.1.2.1.1 Tumor Marker Analysis 65
13.1.2.1.2 CTC Analysis 66
13.1.2.1.3 ctDNA Analysis 67

13.1.2.2 Optional blood samples 68

14 DISCONTINUATION OF STUDY INTERVENTION, PATIENT, STUDY AND SITE 69

14.1 Patients without Intervention 69
14.2 Premature Intervention Discontinuation 69
14.3 Investigator-Initiated Discontinuation of Study Intervention 69
14.4 Patient-Initiated Study Discontinuation vs Withdrawal of Consent 69
14.5 Premature Termination of Study 70
14.6 Premature Termination of Study at a Particular Site 70

15 END OF INTERVENTION (EOI) 71
16 END OF STUDY (EOS) 72

17 ASSESSMENT OF OUTCOME 73
17.1 Evaluation of Local Recurrence 73
17.2 Evaluation of Distant Metastases 73
17.3 Evaluation of Biomarkers 73
18 ASSESSMENT OF HEALTH-RELATED QUALITY OF LIFE 75
18.1 Rationale 75
18.2 Questionnaires 76
18.3 Digital Health Application 76
18.4 Analysis 77
19 DATA HANDLING AND DATA QUALITY ASSURANCE 78
19.1 Data Management and Documentation 78
19.1.1 Data Entry and Queries 78
19.1.2 Data Validation 78
19.1.3 Registration / Recruitment Stop 78
19.1.4 Database Lock 78
19.2 Privacy Protection and Data Safety 79
19.2.1 Pseudonymization 79
19.2.2 User Access Control 79
19.3 Record retention after study completion 79
20 STATISTICS 80
20.1 Randomization and stratification 80
20.2 Analysis Sets 80
20.2.1 Intent-to-Treat (ITT) Set 80
20.2.2 Per Protocol (PP) Set 80
20.3 Sample Size Determination 80
20.4 Patient disposition, data re-coding and display 81
20.5 Analysis of Primary Endpoint(s) 81
20.6 Analysis of Secondary Endpoints 81
20.7 Pre-planned subgroup analyses 82
20.8 Interim/Futility Analyses 82
20.8.1 Interim analyses for efficacy 82
20.8.2 Futility analyses 82
20.8.3 Interim analysis for Quality of Life (QoL) 83
21 INDEPENDENT DATA MONITORING COMMITTEE (IDMC) 84
21.1 IDMC Members and Mission 84
21.2 Documentation Provided to the IDMC 84
21.3 Recommendations of the IDMC 84

22 ADMINISTRATIVE EXECUTION 85

22.1 Monitoring and Auditing 85
22.2 Sponsor's Responsibilities 85
22.3 Investigator's Responsibilities 85
22.4 Patient Informed Consent 86
22.5 Ethics and Regulatory Considerations 86
22.6 Declaration of Helsinki 87
22.7 Study Conduct 87
22.8 Modification of the Protocol 87
22.9 Study Documents 87
22.10 Case Report Forms 87
22.11 Necessary Documents 88
22.12 Archiving 88
22.13 Use of Information and Publication 88
22.14 Finance and Insurance 89

23 INSURANCE COVER 90
24 GLOSSARY OF ABBREVIATIONS AND ACRONYMS 91
25 INVESTIGATOR SIGNATURE PAGE 94
26 APPENDICES 96

26.1 Appendix A: Quality of Life Questionnaires 96

26.1.1 EORTC-QLQ C30 96
26.1.2 PA-F12 98

26.2 Appendix B: Assessment of Performance status and activities of daily living 99

26.2.1 Determination of Performance Status 99

26.3 Appendix C: Deutsche Synopse 100

27 REFERENCES 113

7 INTRODUCTION

Breast cancer is the world's most common cancer entity. After primary therapy of early breast cancer, guidelines limit routine surveillance in breast cancer survivors to clinical surveillance in combination with breast imaging. This approach is based on two large, but outdated cohort studies conducted in the 1980s suggesting no superiority of intensified screening for distant metastasis versus standard surveillance for OS. To date, no other large prospective studies have been conducted addressing the matter. Thus, screening for distant metastases is initiated only in patients with specific symptoms.
We aim to establish a liquid biopsy guided follow-up surveillance method for the sensitive and specific detection of distant metastases before the onset of symptoms. We hypothesize, in consideration of modern therapeutic agents, that earlier detection of distant (oligo-) metastasis and therefore earlier initiation of therapy in a pre-symptomatic stage will improve OS. Furthermore, we expect patient's Quality of Life (QoL) to increase due to intensified follow-up examinations exceeding clinical evaluation.

7.1 Prevalence, Incidence, Mortality of Breast Cancer

Newly diagnosed per year – worldwide: ~ 2.26 million; in Germany: ~ 69 700.
5-year prevalence – worldwide: ~ 7.79 million; in Germany: ~ 300 000.
Incidence Rate – worldwide: 47.8 per 100 000; in Germany: 82.2 per 100 000.
Mean age at first diagnosis: 64 years, affecting all age groups after puberty.
Mortality Rate – worldwide: 13.6 per 100 000 (~ 685 000); in Germany: 16.0 per 100 000 (~ 20 600) (1).

7.2 Burden of disease

On the global incidence of 1.7 million newly diagnosed patients in 2016, breast cancer caused 15.1 million (95% CI, 14.3-16.2 million) DALYs (disability-adjusted life-years), of which 95% came from YLLs (years of life lost) and 5% from YLDs (years lived with disability) (2). Treatment costs and therefore the socioeconomical burden of breast cancer generally increases with higher disease stages at diagnosis. The mean treatment costs of FIGO stage I disease were $29,724. At stages II, III and IV mean costs were 32%, 95%, and 109% higher than those of stage I disease, and the mean treatment costs of locoregional advanced disease and distant breast cancer were 41% and 165% higher than those of local disease. Earlier detection of breast cancer increases the likelihood of cure and therefore reduces treatment costs (3,4).

7.3 Impact of the Study

Routine surveillance in breast cancer survivors is limited to clinical examinations every three months and breast imaging once a year. Screening for distant metastases is only performed in patients with metastasis-specific symptoms. To address patients' needs and to improve OS, we propose a liquid biopsy guided follow-up surveillance method for the detection of distant (oligo-)metastases to enable earlier initiation of therapy in a pre-symptomatic stage.

If liquid biopsy-guided follow-up is found to detect metastatic disease with high sensitivity and specificity, this study could contribute to a paradigm shift in the current follow-up of breast cancer survivors. Such an intensified follow-up could, in turn, lead to an improvement in the QoL of patients as they are regularly screened for a relapse and therefore, if the screening is negative, have the assurance of being disease free. On the other hand, if screening is positive, early detection of distant recurrence before the onset of symptoms would allow earlier initiation of therapy, thereby delaying the onset of symptoms, prolonging survival, and potentially still curing patients. We therefore expect the patients' QoL to improve by the intervention alone, and not only in case of an increased OS. Furthermore, earlier detection of distant recurrence and (oligo-)metastatic disease might reduce the need for cytotoxic chemotherapy and may provide a chance of full recovery in the future, even at the stage of secondary disease. In the long term, this could contribute to a reduction of treatment costs and to more cost-efficient therapies as well as proposing an important link between breast centers and usually resident follow-up doctors.

7.4 Standard Follow-Up Surveillance in Early Breast Cancer Patients

Follow-up surveillance in patients with early breast cancer today is based on two large prospective randomized studies conducted in the 1980s in Italy (5,6). Both aimed to assess different follow-up policies and their impact on survival. In the GIVIO trial (published in 1995), 1320 women with breast cancer stage I-III were randomized to an intensive surveillance including bone scans, liver sonograms and chest X-rays, or to a control regimen. After a median follow-up of 71 months, OS did not differ between groups with 20% and 18% deaths in the intensive and the control group, respectively. No significant differences were apparent in time to detection of recurrence between the two groups (5). In the other trial, conducted by Del Turco et al. and Palli et al. (published in 1994), 1243 women with early breast cancer were also randomized to conventional or intensive follow-up including bone scans and chest X-rays. Increased detection of isolated pulmonary and bone metastases was evident in the intensive compared to the clinical follow-up group (112 vs. 71 cases), while no difference was observed for other sites and for local and/or regional recurrences. The 5-year relapse-free survival rate was significantly higher for the clinical follow-up group, with patients in the intensive follow-up group showing earlier detection of recurrences, whereas no difference in 5-year OS (81,4% (intensive) vs. 80,5% (conventional)) was observed (6).
Based on this evidence, national and international guidelines recommend standard follow-up without imaging (except for mammography) for asymptomatic breast cancer patients. In Germany patients are encouraged to visit their doctors every three months for the first three years after diagnosis and every six months for the consecutive two years. Here, a clinical evaluation on the patients' health will be performed, adherence to any adjuvant therapy will be checked, follow-up subscriptions will be issued and yearly mammograms will be scheduled.
However, these trials have been conducted in an era of outdated technology and limited therapeutic options. It is possible that recent improvements in diagnostics and early, target-oriented treatments could lead to long-term remissions and improve both, survival and QoL outcomes. Some of the most important improvements in breast cancer treatment include: enhancement of endocrine therapy (aromatase-inhibitors, GnRH-analogues, new SERMs, CDK 4/6 inhibitors, mTOR inhibitors, PI3K-inhibitors), modern cytostatic treatment regimens (including taxane-based chemotherapy, Capecitabine, Gemcitabine, Vinorelbine, Eribulin) and reduced cytotoxicity due to supporting agents

(especially modern antiemetics), biological targeted therapies like monoclonal antibodies (Trastuzumab, Pertuzumab, Atezolizumab) and antibody-drug-conjugates (Trastuzumab-emtansine, Trastuzumab-deruxtecan, Sacituzumab-govitecan), immunotherapies (Atezolizumab, Pembrolizumab) other targeted treatments like tyrosine-kinase-inhibition or PARP-inhibition as well as improved locoregional therapy. Many of these procedures have shown to improve overall survival, especially when conducted in early phases of metastatic disease (7–12). In addition, survival depends on when treatment is started after diagnosis of metastatic disease (13).
Despite the feasibility of these new therapies, there have not been any new studies about the impact of intensified follow-up for breast cancer patients yet, with the last Cochrane review dating back to 2016, lacking additional recommendations (14). Thus, research is necessary to improve breast cancer follow-up strategies, as aftercare visits today are heterogeneous. We aim to establish a concept to improve the follow-up surveillance for patients with early breast cancer and to change the current standard of care for this patient group. As cancer therapies become increasingly individualized, molecular biomarkers represent a key technique in aiming to predict the prognosis or monitor treatment response.

7.4.1 Rationale for Tumor markers in Liquid biopsy-based Follow-Up

Soluble MUC1 has been discussed as a biomarker for predicting prognosis, treatment efficacy, and monitoring disease activity in breast cancer patients, as clear associations were found between tumor size/burden and soluble MUC1 levels (15–19). Immunoassays for CA15.3 and CA27.29 target the epitopes on the same MUC1 and are considered identical. They are well-known assays that allow the detection of circulating MUC1 antigen in peripheral blood. CEA levels are usually less commonly elevated than the MUC1-assay, but studies suggest an additional value through gaining sensitivity by testing CEA levels in addition to CA27.29/CA15.3 (20). As with MUC1 levels, a clear correlation between tumor size/burden and CEA levels is reported (15,21). While the American Society of Clinical Oncology (ASCO) recommends using CA27.29/CA15.3 and CEA only in conjunction with diagnostic imaging for treatment monitoring of patients with metastatic disease, their role during follow-up of early-stage breast cancer remains unclear (20,22). Several studies could show that an increase in CA27.29/CA15.3 and CEA after primary and/or adjuvant therapy may predict distant recurrence before the onset of symptoms or (imaging) tests (23,17). However, prospective randomized clinical trials to demonstrate whether detection and treatment of these asymptomatic metastases impact on the most significant outcomes (OS, QoL) are lacking. MUC16-based CA125 is commonly used to monitor ovarian cancer, but has rarely been studied in detecting breast cancer recurrence. Yet, it has shown sensitivity in breast cancer (24,25), especially when used in addition to MUC1 based CA27.29/CA15.3 and CEA, through which sensitivity of detecting metastasis could be increased by 21.3%, while maintaining a high specificity (26). Heterogeneity of CA125 in breast cancer is mainly associated with the sites of metastasis as high CA125 levels are associated with visceral or pleural, rather than with bone or soft tissue involvement. In general, elevated levels of these tumor markers are associated with worse prognosis (27,28), and retrospective studies suggest that not a cut-off based evaluation, but a kinetics based approach (increase-delta from an individual baseline) should be used to increase sensitivity and specificity in detecting metastatic disease or disease progression (26,29,30). The most feasible cut-off values for increasing tumor markers have been reported as 75% for CA15.3, 100% for CEA and 150% for CA125 (29), where a sensitivity of 72.3% and a specificity of 99% could be

achieved (data from the “Münchner Nachsorgestudie Großhadern”, Dr. P. Stieber, personal communication).
While interchanging CA15.3 and CA27.29 testing is not advised, results are usually similar and concordant (20,31,32). An application of the presented principles for CA15.3 on CA27.29 testing appears unproblematic.

7.4.2 Rationale for CTCs in Liquid biopsy-based Follow-Up

An early hematogenic spread of tumor cells is observed in some patients with early breast cancer. These cells can be detected in the peripheral blood, are known as circulating tumor cells (CTCs) and are reported widely as a valuable prognostic factor, both at time of primary diagnosis and in the context of distant metastases (33–35). In metastasized patients, the detection of CTCs itself and especially over 5 CTCs in 7,5 mL of blood is reportedly associated with poor prognosis and a lower life expectancy (36,37). Currently, the AGO-guidelines assess the detection of CTCs in metastasized patients as useful in particular cases to assist clinical decision-making. In the SUCCESS-trial conducted by our study group, we demonstrated the prognostic relevance of circulating tumor cells in peripheral blood of early breast cancer patients before and after adjuvant treatment. Persistence of CTCs after adjuvant chemotherapy demonstrated an unfavorable prognosis (38), which confirmed the data of other studies suggesting CTCs as an independent prognostic factor for early relapses and aggressive tumor behavior (39–41).

7.4.3 Rationale for ctDNA in Liquid biopsy-based Follow-Up

Breast cancer genomes may harbor from few to hundreds of rearrangements and mutations per tumor (42). Whereas somatic point mutations can be present in various tumor types and individuals, chromosomal rearrangements are individual and highly tumor specific, serving as a distinctive genetic signature of a tumor (43). ctDNA has been shown to mirror the mutational signature of the primary tumor (analyzed via exome sequencing), is detectable the in blood plasma of patients with advanced malignancies and shows increasing potential as a non-invasive biomarker for monitoring tumor growth and response to treatment (44). During follow-up, where patients’ plasma usually contains very few tumor-derived DNA fragments, detection of ctDNA can be challenging. However, by using information from primary tumor tissue, it is possible to design personalized sequencing panels to track patient-specific tumor mutations and to highly increase sensitivity (45)(46). The use of ctDNA during breast cancer follow-up via personalized ctDNA profiling has been described as highly sensitive, generating a lead-time in the ctDNA detection preceding the clinical detection of metastasis up to two (47), respectively three years (48). Both studies showed a specificity of 100%, with no non-relapsing patients being ctDNA-positive at any timepoint. Nevertheless, there are to date no prospective, randomized clinical trials to analyze the clinical validity of conventional tumor markers, CTCs and ctDNA combined with diagnostic imaging that will eventually lead to a therapeutic intervention, which is where our proposed study will close the gap.
To support our approach, we conducted a blinded pilot study during the development phase of this study. During this pilot study, a retrospective analysis of tumor and blood samples from early breast cancer patients with known (but blinded) clinical outcome was conducted using the RaDaR™ (Residual Disease and Recurrence) assay by Inivata Inc. It was shown that in 15 of 21 patients with confirmed

clinical distant recurrence, ctDNA could be detected. Out of the 6 remaining patients, 5 suffered from local recurrence without evidence of distant metastasis. These results indicate that the RaDaR™ assay is well suited as a ctDNA-based approach for early detection of disease relapse (49).

7.5 Appropriateness of the Primary Endpoint

Overall Survival is defined as the time from randomization in this study until death of any cause. OS is considered to be a gold-standard endpoint in oncological studies leading to a good comparability to other studies on a global level. Furthermore, it is easily measured, unbiased and clinically significant. To evaluate the benefit of an intensified, liquid-biopsy based surveillance, the OS seems to be appropriate. Recurrence- or disease-free survival (DFS) as well as distant disease-free survival (DDFS) as a surrogate marker for overall survival are not adequate in this study, as in case of positive lead time, DFS and DDFS are shortened in the experimental group, while OS might be positively influenced at the same time because of early treatment intervention.

As our second primary objective, we will determine whether an Overall Lead-Time effect can be generated in the follow-up of early breast cancer patients due to tumor marker/CTC/ctDNA guided surveillance compared to standard surveillance after primary therapy. Overall Lead Time Effect is a composite measure, defined as the time from molecular to via imaging verified distant recurrence lead time plus the difference in time to distant recurrence between the two arms (i.e., difference between median time from randomization to distant recurrence for all patients with distant recurrence in the Standard Surveillance arm and median time from randomization to distant recurrence for all patients with distant recurrence in the liquid-biopsy guided Intensive Surveillance arm; see Figure 2). This will be assessed only for all markers in combination (as this composite endpoint cannot be calculated for each marker separately). The Overall Lead Time Effect will be a surrogate parameter for the time gained by using liquid biopsy to detect a recurrence. As we hope to detect distant recurrence of disease earlier, these patients will be treated earlier with the applicable therapy regimen. It has been shown that a delay in treatment can result in poorer overall survival in invasive non-metastatic as well as metastatic breast cancer. Delayed first treatment (>90 vs ≤30 days postdiagnosis) was associated with worse OS in patients with both invasive nonmetastatic and metastatic breast cancer (50), while another study in metastatic breast cancer patients found that a treatment delay of 12-24 weeks compared to a treatment delay of 4-12 weeks was associated with greater risk of death from first treatment (13). Thus, in both of these studies, the time lost due to later start of therapy was associated with a poorer OS. By investigating and evaluating the Overall Lead Time Effect, we aim to quantify the time gained through intensified liquid biopsy-based monitoring to treat the recurrent breast cancer earlier and therefore improve OS.

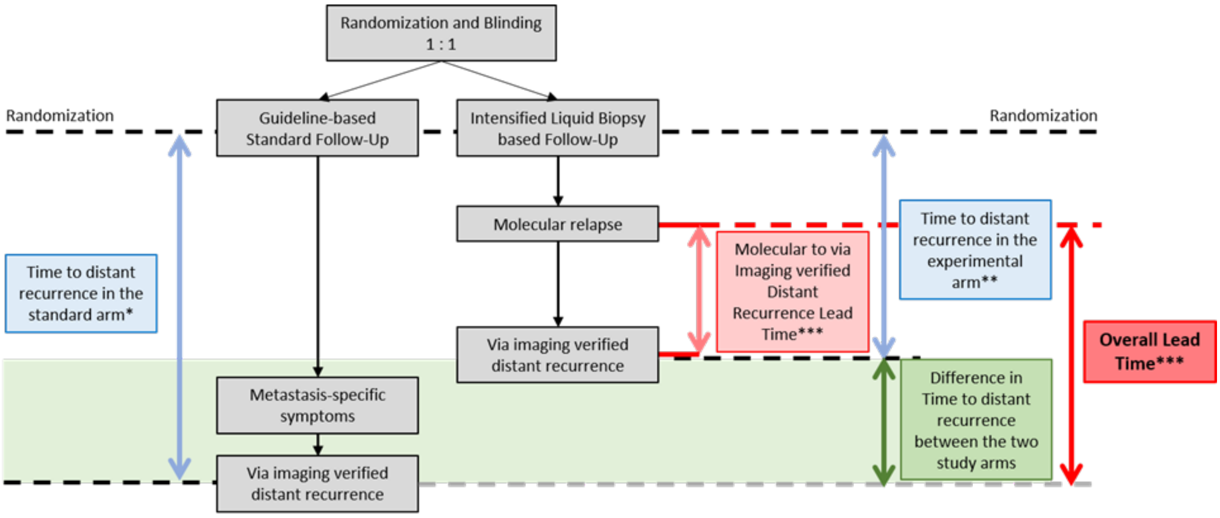

* median calculated for all patients in the standard arm with a via imaging verified distant recurrence
** median calculated for all patients in the experimental arm with a via imaging verified distant recurrence
*** median calculated for all patients in the experimental arm with a via imaging verified distant recurrence

Figure 2: SURVIVE Study Design with indicated Overall Lead Time and other relevant timepoints for calculation

7.6 Risk – Benefit Analysis for the Participants

In addition to routine aftercare practice, patients participating in this study will receive regular blood-drawals to have the chance of being monitored by liquid-biopsy based procedures. If imaging is triggered and metastases are detected, the time to receive treatment is potentially shorter compared to the Standard Surveillance arm. This may have an impact on OS on the one hand, but will introduce anticancer therapy at an earlier timepoint, causing side effects and possibly a deterioration of QoL.
In the Standard Surveillance arm, the patients will have no direct benefit from participating in this study. Nevertheless, the standard of current aftercare in breast cancer patients is always met, thus they have no disadvantage to patients not participating in this study, receiving standard aftercare.
Risks participating in this study mainly arise from drawing blood, where swelling, pain, bleeding, hematoma, infection or fainting may be commonly observed. However, standard procedures of care e.g., disinfection of the site and using pressure to prevent bleeding and consecutive hematoma will be taken by the participating study centers. If imaging is triggered, a CT scan will be performed. The American Food and Drug Administration (FDA) describes the risks of a CT scan as the possibility of finding something benign that could lead to unnecessary diagnostics and as a source of radiation (51). The risk of developing radiation-induced cancer is said to be very low in standard CT scans (52) and widely discussed. As a preemptive measure, we limit the number of consecutive CT scans to a maximum of four CT scans per year. Further disadvantages by participating in this study may include: Extra commitment and time spent by additional appointments at the study center and constant reminders of a potentially threatening illness at a stage in life, where most patients have carried on with their lives and would otherwise solely see their personal Ob/Gyn for aftercare visits. This may result in a deterioration of QoL compared to patients not participating in this study.

Reasons for discontinuation of the study are outlined in section 14.
Balancing the risk of interventions and potential benefits, we conclude that the anticipated benefit of earlier detection of recurrence outweighs the risks and is medically justified.

7.7 Interpretation of Potential Study Results

Different scenarios of results may lead to the following differential conclusions.
A higher overall survival could be seen in the Intensive Surveillance arm which would lead to a change in standard surveillance for breast cancer and make way to establish liquid biopsy as a screening tool. If a higher or equal overall survival could be seen in the Standard Surveillance arm, standard of care will be proven and nothing will change in the current standard. This interpretation is also valid for an observed difference in the secondary objectives such as Invasive disease-free survival (IDFS), distant disease-free survival (DDFS) and distant recurrence-free survival (DRFS). If a higher Overall Lead Time Effect can be shown in the Intensive Surveillance group, the use of liquid-biopsy can be proven as a useful tool to detect recurrence of disease earlier and might lead to earlier treatment intervention. If no Overall Lead Time Effect can be seen, standard of care will persist. If sensitivity and specificity of the liquid biopsy markers used (CA27.29, CEA, CA125, CTC, ctDNA) is seen as high for one marker or a combination of markers, establishing these specific markers in the aftercare as an extra tool to monitor patients is a possibility. If sensitivity and/or specificity of one or multiple markers is seen as low, the concerning marker may be discarded as a screening tool. Depending on the Molecular to via Imaging verified Recurrence Lead Time in the Intensive Surveillance arm, possible timepoints for future treatments of the concerning patients can be hypothesized and establish further grounds for future interventional drug studies.
Regarding QoL, the influence of blood sampling, liquid biopsy results and imaging on quality of live has to be studied and monitored thoroughly. If a higher QoL is seen in the Intensive Surveillance arm, further encouragement to implement liquid biopsy could be given, as liquid biopsy and consecutive imaging if necessary, could elevate the patients' feeling of being closely watched and not left alone. If QoL is lower in the Intensive Surveillance arm, liquid biopsy and consecutive imaging could be seen as a source of frightening patients and reminding them that they had cancer instead of reassuring them of their health. The potential influence has to be balanced with potential benefits and risks outside the spectrum of QoL.
As the field of research regarding liquid-biopsy based decision-making on therapeutic interventions is rapidly evolving, a therapeutic intervention for patients with positive findings in their liquid biopsy during this study could be a possibly scenario and would require an amendment of this protocol.

8 STUDY OBJECTIVES

8.1 Primary Objectives

Overall Survival (OS)
To compare 5-year OS rates between patients in the **Standard Surveillance** arm versus patients in the liquid-biopsy guided **Intensive Surveillance** arm.

Overall Lead Time Effect
To determine the Overall Lead Time Effect generated due to tumor marker/CTC/ctDNA guided **Intensive Surveillance** compared to **Standard Surveillance** after primary therapy in early breast cancer patients.

8.2 Secondary Objectives

Invasive disease-free survival (IDFS)
To compare IDFS between patients in the **Standard Surveillance** arm versus patients in the liquid-biopsy guided **Intensive Surveillance** arm.

Distant disease-free survival (DDFS)
To compare DDFS between patients in the **Standard Surveillance** arm versus patients in the liquid-biopsy guided **Intensive Surveillance** arm.

Distant recurrence-free survival (DRFS)
To compare DRFS between patients in the **Standard Surveillance** arm versus patients in the liquid-biopsy guided **Intensive Surveillance** arm.

Breast cancer specific survival (BCSS)
To compare BCSS between patients in the **Standard Surveillance** arm versus patients in the liquid-biopsy guided **Intensive Surveillance** arm.

Invasive breast cancer free survival (IBCFS)
To compare IBCFS between patients in the **Standard Surveillance** arm versus patients in the liquid-biopsy guided **Intensive Surveillance** arm.

Overall Survival (OS) after 10 Years
To compare 10-year OS rates between patients in the **Standard Surveillance** arm versus patients in the liquid-biopsy guided **Intensive Surveillance** arm.

Molecular to via Imaging verified Recurrence Lead Time in the Interventional Arm
To determine the Lead Time (median, interquartile, range, 95% confidence interval) generated in the liquid-biopsy guided **Intensive Surveillance** arm.

Quality of life (QoL) with questionnaires: EORTC QLQ-C30 and PA-F12
To compare QoL between patients in the **Standard Surveillance** arm versus patients in the liquid-biopsy guided **Intensive Surveillance** arm.

Liquid biopsy sensitivity (CA27.29, CEA, CA125, CTC and ctDNA)
To determine sensitivity (i.e., the true positive rate) of the liquid biopsy markers in combination (and individually where applicable) in the liquid-biopsy guided **Intensive Surveillance** arm.

Liquid biopsy specificity (CA27.29, CEA, CA125, CTC and ctDNA)
To determine specificity (i.e., the true negative rate) of the liquid biopsy markers in combination (and individually where applicable) in the liquid-biopsy guided **Intensive Surveillance** arm.

Liquid Biopsy False-Positive Rate (CA27.29, CEA, CA125, CTC and ctDNA)
To determine the false-positive rate of the liquid biopsy markers in combination (and individually where applicable) in the liquid-biopsy guided **Intensive Surveillance** arm.

Liquid Biopsy False-Negative Rate (CA27.29, CEA, CA125, CTC and ctDNA)
To determine the false-negative rate of the liquid biopsy markers in combination (and individually where applicable) in the liquid-biopsy guided **Intensive Surveillance** arm.

Rate of liquid biopsy positivity (CA27.29, CEA, CA125, CTC and ctDNA)
To determine the overall rate of positive liquid biopsy marker results in combination (and individually where applicable) in the liquid-biopsy guided **Intensive Surveillance** arm.

8.3 Translational Research Objectives

To ensure the possibility of retrospective studies during and after the ongoing study, a biobank will be implemented.

9 STUDY ENDPOINTS

9.1 Primary Endpoints

Overall Survival (OS)
OS is defined as time from randomization until the death of the patient independent of cause of death. If a patient is not known to have died, OS is censored at the date of last contact.

Overall Lead Time Effect
This endpoint is a composite measure, defined as the median time from molecular to via Imaging verified Recurrence Lead Time (calculated only for patients in the liquid-biopsy guided Intensive Surveillance arm; see secondary endpoint definition below) + Difference in time to distant recurrence between the two arms (i.e., difference between median time from randomization to distant recurrence for all patients with distant recurrence in the Standard Surveillance arm and median time from randomization to distant recurrence for all patients with distant recurrence in the liquid-biopsy guided Intensive Surveillance arm; see Figure 2). The Overall Lead Time Effect will be assessed for all markers in combination.

9.2 Secondary Endpoints

Invasive disease-free survival (IDFS)
IDFS is defined as time from randomization until first IDFS event, including any invasive ipsilateral, regional, contralateral, and distant disease recurrence, second primary tumors, or death from any cause as event; non-invasive, in-situ cancer events are excluded. If a patient has not had an event, IDFS is censored at the date of last adequate tumor assessment.

Distant disease-free survival (DDFS)
DDFS is defined as time from randomization until first DDFS event including metastasis, second primary tumors and death from any cause as event. If a patient has not had an event, DDFS is censored at the date of last adequate tumor assessment.

Distant recurrence-free survival (DRFS)
DRFS is defined as time from randomization until first DRFS event including metastasis and second primary tumors; death from any cause is not included as event. If a patient has not had an event, DRFS is censored at the date of last adequate tumor assessment.

Breast cancer specific survival (BCSS)
BCSS is defined as time from randomization until breast cancer associated death of the patient. If a patient is not known to have died, BCSS is censored at the date of last contact. If a patient has died for reasons not associated with breast cancer (by clinical assessment), BCSS is censored at the date of death.

Invasive breast cancer free survival (IBCFS)
IBCFS is defined as time from randomization until first IBCFS event, including any invasive ipsilateral, regional, contralateral and distant disease recurrence or death from any cause as event; non-invasive, in-situ cancer events are excluded. If a patient has not had an event, IBCFS is censored at the date of last adequate tumor assessment.

Overall Survival (OS) after 10 Years
OS is defined as time from randomization until the death of the patient independent of cause of death. If a patient is not known to have died, OS is censored at the date of last contact.

Molecular to via Imaging verified Recurrence Lead Time in the Interventional arm
The Lead Time is defined as time from first molecular relapse (definitions see above and in Figure 2) to via imaging verified recurrence for all patients in the liquid-biopsy guided Intensive Surveillance arm for whom a via imaging verified recurrence is documented during the 5-year interventional period of the study. If a patient has a via imaging verified recurrence but no documented molecular relapse, the Lead Time for this patient is set to ‘0’. This secondary endpoint will be assessed for all markers in combination and, where applicable, for each marker separately.

Quality of life (QoL) with questionnaires: EORTC QLQ-C30 and PA-F12
QoL will be monitored in both groups using 2 questionnaires which are to be completed every 6 months (EORTC QLQ-C30 and PA-F12) in the interventional phase (first five years) and once a year during the five years of follow-up. QoL data will be collected on paper or via a digital health application.

Liquid biopsy sensitivity (CA27.29, CEA, CA125, CTC and ctDNA)
Sensitivity is defined as the proportion of all patients in the liquid-biopsy guided Intensive Surveillance arm with a recurrence as verified by imaging during the 5-year interventional period of the study that had a positive biomarker result (i.e., molecular relapse) within 36 months before the recurrence as verified by imaging occurred (or within 60 months before the recurrence as verified by imaging occurred if in the first 36 months at least once a year a positive biomarker is measured).

Liquid biopsy specificity (CA27.29, CEA, CA125, CTC and ctDNA)
Specificity is defined as the proportion of all patients in the liquid-biopsy guided Intensive Surveillance arm with no recurrence as verified by imaging during the 5-year interventional period of the study that had only negative biomarker results (i.e., no indication of molecular relapse) within 36 months before the end of the 5-year interventional period of the study.

Liquid Biopsy False-Positive Rate (CA27.29, CEA, CA125, CTC and ctDNA)
False-positive rate is defined as the proportion of all patients in the liquid-biopsy guided Intensive Surveillance arm with a positive biomarker result (i.e., molecular relapse) during the 5-year interventional period of the study that had no recurrence as verified by imaging within 36 months after first molecular relapse.

Liquid Biopsy False-Negative Rate (CA27.29, CEA, CA125, CTC and ctDNA)
False-negative rate is defined as the proportion of all patients in the liquid-biopsy guided Intensive Surveillance arm with only negative biomarker results (i.e., no indication of molecular relapse) that had a recurrence as verified by imaging during the 5-year interventional period of the study.

Rate of liquid biopsy positivity (CA27.29, CEA, CA125, CTC and ctDNA)
The overall rate of liquid biopsy positivity is defined as the proportion of all patients in the liquid-biopsy guided Intensive Surveillance arm that had at least one positive biomarker result (i.e., molecular relapse) during the 5-year interventional period of the study.

9.3 Translational Research Endpoints

A biobank will allow the storage of unused biomaterials from both, the Intensive Surveillance arm as well as the Standard Surveillance arm. These materials will be used for future retrospective studies.

10 STUDY DESIGN

10.1 Description of the Study Design

This is a partially double-blinded, multi-center, randomized, controlled superiority study to evaluate the potential benefits of intensified surveillance versus standard surveillance in medium-risk and high-risk early breast cancer patients.

3500 patients will be randomized after completion of primary anti-tumor therapy (adjuvant chemotherapy, surgery or radiotherapy, whichever occurs last) in a 1:1 ratio to receive:

- **Standard Surveillance** according to national guidelines or
- **Intensive Surveillance** with additional testing of blood samples for prospective tumor markers (CA27.29, CA125, CEA), CTC and ctDNA

In both study arms, patients will continue to receive standard surveillance according to national guidelines, including clinical follow-up visits every 3 months for the first 3 years and every 6 months for the following 2 years after study enrollment. Additionally, blood samples will be drawn and QoL will be analyzed at these clinical follow-up visits in both arms.

In the **Standard Surveillance** group all blood samples will be stored in a biobank.

In the **Intensive Surveillance** group blood samples will be tested for prospective tumor markers (CA27.29, CA125, CEA), CTCs and ctDNA. Abnormal findings of either marker (CA27.29 or CA125 or CEA or CTC or ctDNA) will trigger diagnostic imaging. Additionally, blood samples will be stored in a biobank for retrospective analysis. In both study arms detection of distant recurrence will terminate the surveillance protocol and treatment will be initiated according to national guidelines.

Blinding will be partial, as individual patients in the **Intensive Surveillance** arm, where the liquid biopsy shows an indication for molecular residual disease (MRD), will receive further assessments and must therefore be unblinded.

After the 5-year interventional period, 5 years of follow-up will follow where data is collected once a year (questionnaire directed towards the patient's living status, wellbeing and QoL).

Planned enrollment period is approximately 24 months, total study duration is approximately 148 months (2-year recruitment period, 5-year interventional period, 5-year follow up period). In terms of long-term follow-up after end of study, patients have the possibility to participate in the patient self-reporting registry (Patientenselbstauskunft).

Randomization will be stratified using block randomization by:

- Hormone receptor status (negative vs positive)
- HER2 status (negative vs positive)
- Histological lymph node status at surgery ((y)pN0 vs (y)pN+)

10.2 Study design

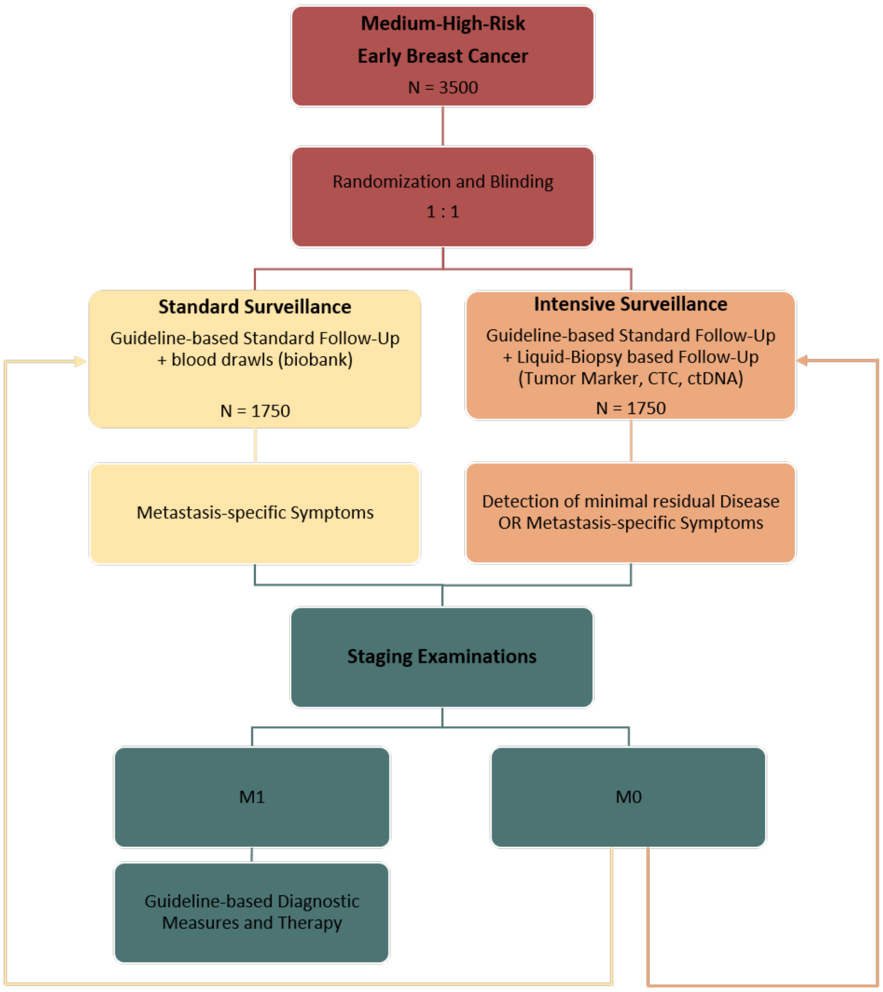

Figure 3: Study Design

11 STUDY POPULATION

Investigators should consider each of the criteria outlined below when selecting a patient for this study. In order to participate in the study, a patient must fulfill all inclusion criteria and must not meet any of the exclusion criteria. Investigators should also consider all other relevant medical and non-medical factors, as well as the risks and benefits of the study, when deciding if a patient is an appropriate candidate for this study.

11.1 Number of Patients

Target accrual for this study will be 3500 randomized patients with a hypothesized number of 3850 patients assessed for eligibility, corresponding to an expected screening failure rate of approximately 10%. Patients who have been randomized but did not complete all follow-up visits with the appropriate assessments are considered drop-outs.

If a personalized ctDNA assay cannot be established for a patient in the Intensive Surveillance arm due to problems related to quality and/or quantity of the collected primary tumor tissue, a second primary tumor tissue sample from this patient will be obtained to facilitate another try at establishing a personalized ctDNA assay. If it still is not possible, this already randomized patient will be accounted for as drop-out.

11.2 Inclusion Criteria

Patients will be eligible for study participation only if they comply with the following criteria:

1. Written informed consent for all study procedures according to local regulatory requirements prior to beginning specific protocol procedures.
2. Unilateral or bilateral primary invasive carcinoma of the breast, confirmed histologically.
3. Patients with intermediate- to high-risk early breast cancer defined as either
    - an indication for (neo-)adjuvant chemotherapy (regardless whether performed or not), and/or
    - Large tumor (> 50 mm), and/or
    - Positive lymph nodes (> pN1mi), and/or
    - High grade (≥ G3).

   Indication to (neo-)adjuvant chemotherapy is seen as stated in the German S3 guideline for breast cancer (53) as well as stated in the guidelines from the AGO (54).
4. A complete resection of the primary tumor, with resection margins free of invasive carcinoma.
5. Completion of primary anti-tumor therapy (adjuvant chemotherapy, surgery or radiotherapy, whichever occurs last) no more than 24 months previously. Enrollment of patients during any kind of adjuvant therapy except chemotherapy (e.g., but not limited to endocrine therapy, antibody therapy, CDK4/6-inhibitors, PARP inhibitors, PI3K inhibitors, antibody-drug conjugates and other novel agents) is allowed. Patients with Luminal A/B breast cancer (ER/PGR positive, HER2 negative/low) may be enrolled up to 60 months after completion of primary anti-tumor therapy.

6. Availability of primary tumor tissue from core biopsy or surgical removed tissue (FFPE Slide (≥ 6 mm³, min. 10 slides, thickness: 5 µm-10 µm, area >150 mm² and 1 H&E stained slide, minimum 20% tumor content) or FFPE Block (≥ 6 mm³ thickness: 100 µm, area: >150 mm² and 1 H&E stained slide, minimum 20% tumor content) or Genomic DNA extracted from FFPE slides or block (≥ 600 ng, Minimum volume: 25 µL, concentration: 20 ng/µL, buffer: 10 mM Tris pH 8, 1 mM EDTA)) at timepoint of enrollment.

   - Patients with primary systemic therapy: tissue from core biopsy
   - Patients receiving surgery as primary therapy: surgically removed cancer tissue or tissue from core biopsy.

   Important note: Tumor tissue of both sides will be needed in case of bilateral breast cancer.
7. No current clinical evidence for distant metastases.
8. Females or males ≥ 18 years and ≤ 75 years of age.
9. Performance status ≤ 1, Eastern Cooperative Oncology Group (ECOG) scale.
10. Patient must be willing and able to comply with scheduled visits, treatment plans, laboratory tests, and other study procedures.

It is possible for patients to participate in adjuvant interventional breast cancer trials and in interventional trials that are based on MRD detection without metastases being confirmed via imaging. These interventional trials should be defined therapy-intervention trials that are evidence-based and have to be approved by the SURVIVE Steering Committee.

11.3 Exclusion Criteria

Patients will be ineligible for study participation if they comply with the following criteria:

1. Patients with a history of any secondary primary malignancy are ineligible with the following exceptions:
   - in situ carcinoma of the cervix or
   - adequately treated basal cell carcinoma of the skin or
   - ipsi- or contralateral non-invasive carcinoma of the breast (DCIS).
2. Patients in pregnancy or breastfeeding.
   If a patient gets pregnant during the participation in the interventional phase of the study (Year 1-5), an end of intervention visit will be scheduled and the patient will enter the follow-up phase of the study (see section 15 for further information). Pregnancy during the follow-up phase of the study is to be reported but does not lead to an exclusion of the study.
3. History of significant neurological or psychiatric disorders including psychotic disorders, dementia or seizures that would prohibit the understanding and giving of informed consent.
4. Renal insufficiency with GFR < 30 mL/min.
5. Previous or concomitant cytotoxic or other systemic antineoplastic treatment that is not used for treating the primary breast cancer.

12 STUDY INTERVENTIONS

This is a partially double-blinded, multi-center, randomized, controlled superiority study. Patients will be randomly assigned to one of the two groups (**Intensive Surveillance** versus **Standard Surveillance**) in a 1:1 ratio.

We will acquire peripheral blood samples from patients in both groups as a tertiary diagnostic intervention every 3 months for the first 3 years after study inclusion, thereafter every 6 months for another 2 years. Peripheral blood samples from patients in the **Standard Surveillance** group will be stored in a biobank for translational studies and retrospective analyses. Peripheral blood samples from patients in the **Intensive Surveillance** group are collected to test CA27.29, CEA and CA125 serum levels as well as ctDNA and CTC levels. As an abnormal increase in these tumor marker levels or detection of ctDNA or CTC correlate closely with tumor load, further examinations will be initiated for patients with abnormal tests in the Intensive Surveillance group. Patients with standard follow-up on the other hand will have access to further examinations if metastasis-specific symptoms occur, according to current German guidelines.

12.1 Standard Surveillance

Current aftercare for early breast cancer in Germany begins after completion of primary anti-tumor therapy, which can be adjuvant chemotherapy, surgery or radiotherapy, whichever occurs last. For the first 3 years of aftercare, visits at the aftercare specialist (gynecologist/family physician/oncologist) should be scheduled every 3 months. For the following 2 years, visits every 6 months are recommended. After five years, the frequency of visits is usually reduced to once a year, noting that no official guidelines currently address intervals after the first 5 years after primary diagnosis. In these visits, patients should be asked about their well-being, about possible symptoms for distant metastases and about their therapy adherence if still under therapy with e.g., endocrine treatment. Recommendations about lifestyle should be given, i.e., preferring a healthy diet, reduction of alcohol consumption, abstention from nicotine and practising physical activity on a regular basis. Furthermore, a clinical examination of the breasts should be performed including inspection and palpation of the breasts and locoregional lymph nodes. Imaging in the form of mammography with or without supplementary sonography of the breasts is recommended every 12 months. Magnet resonance imaging (MRI) is only to be considered, if mammography and sonography are inconclusive. Monthly self-palpation of the breasts is recommended for patients.

Other imaging modalities, including CT scans, are only recommended if there are symptoms suspicious of local recurrence or distant metastases. Blood tests should only be performed to assess side effects, e.g., when the patient is under endocrine treatment.

Determining tumor markers and imaging other than breast imaging is explicitly not advised in asymptomatic patients.

The aims of current standard aftercare are stated by the AGO in Germany to be the early detection of locoregional recurrence, of contralateral breast malignancy, and of symptomatic metastases. In addition, aftercare should try to improve overall QoL, ensure adherence to adjuvant treatment, manage side effects and include psychosocial care (55). Detection of asymptomatic metastases is currently not of interest.

12.2 Intensive Surveillance

All measures are taken in addition to standard surveillance.
Staging examinations will be performed if one or more of the markers (tumor markers, CTC, ctDNA) show an abnormal result. A normal result of one marker cannot prevent further measures, if not indicated differently below.

12.2.1 Determination of Tumor markers

For tumor marker analysis, no absolute cut-offs will be used, but a delta in tumor marker increase (CA27.29 +75% or CEA +100% or CA125 +150%) from baseline. The baseline will be defined as the mean value of two blood drawings four weeks apart after randomization for each patient individually (28 $\pm$ 5 days). After determination of the individual baseline, blood samples will be drawn every three months for three years, thereafter every six months for another two years.
Abnormal tumor marker serum level increase from baseline (CA27.29 +75% or CEA +100% or CA125 +150%) will trigger complete standard staging examinations (CT scan of the chest and abdomen as well as SPECT bone scan). In case of M0, the patient will continue liquid-biopsy based tumor marker testing. If tumor marker elevation continues, a maximum of three consecutive negative staging examinations will be performed before another rise in tumor marker will be mandatory (according to the deltas stated above) to trigger further imaging (compare the complete workflow in Figure 4). If repeated, the staging examination will be performed via CT scan +/- a SPECT bone scan. The minimum interval between two CT scans should be at least 3 months, and the minimum interval between two SPECT bone scans should be at least 6 months at any time point.
All staging examinations are done according the national guidelines for breast cancer.
CA27.29, CEA and CA125 will be measured with the AIA®-CL1200 by TOSOH BIOSCIENCE (TOSOH CORPORATION, Tokyo, Japan). CA125 corresponds to OVCA TEST CUP. Specimen will be collected in one standard serum sampling tube (7,5 mL whole blood), and shipped for centralized evaluation. Serum probes will be stable for 48h at 20-25°C, for 7 days at 2-8°C and thereafter at -20°C. Principles of analysis and probe handling is explained further in 13.1.2.1.1 and in the according leaflets (56).

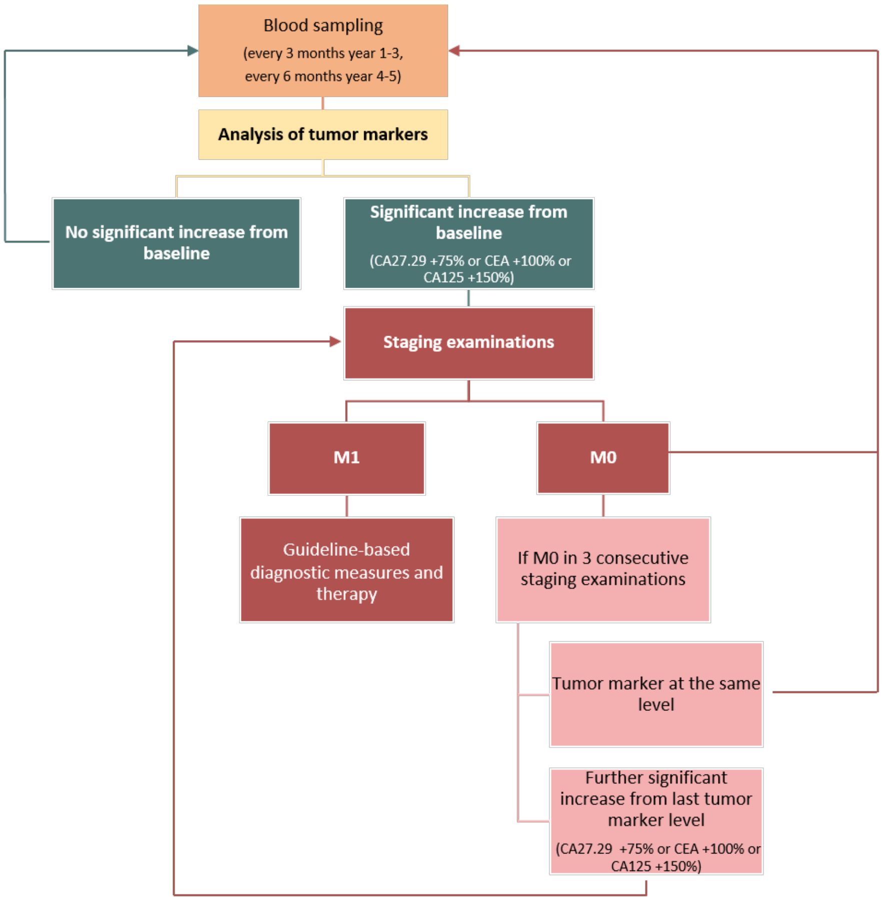

Figure 4: Pathway for Tumor Marker Analysis and Consequences

12.2.2 Determination of CTC levels

If an abnormal tumor marker value is detected in a patient in the Intensive Surveillance arm for the first time, CTC analysis will be initiated at the next regular study visit. Furthermore, all patients in the Intensive Surveillance arm will receive CTC testing one year after study enrollment. If ≥ 1 CTC is detected, patients will undergo complete standard staging examinations. In case of M0, the patient will continue standard liquid-biopsy based testing.

CTCs will be analyzed using the FDA-approved, standardized semiautomatic CellSearch® System (Menarini Silicon Biosystems). Whole blood samples will be collected in 3 x 10 mL CellSave preservative tubes and analyzed centrally within 96 h of collection (stable at room temperature) aiming for a numeration of CTCs – nucleated cells of epithelial origin (CD45-, EpCAM+, cytokeratin 8+ / 18+ and/or 19+) (41).

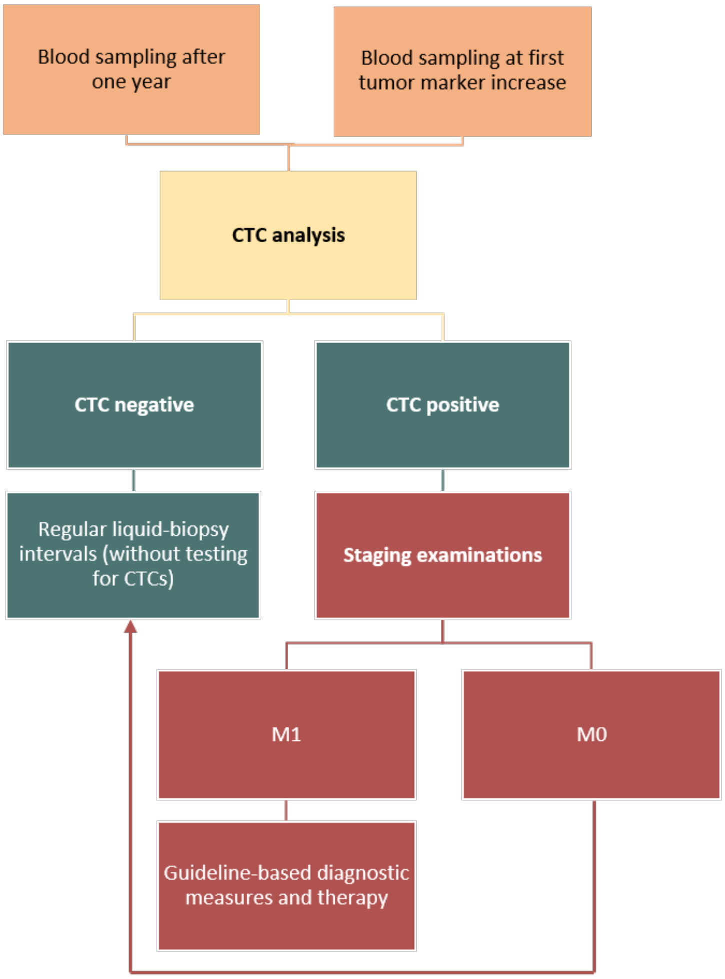

Figure 5: Pathway for CTC Analysis

12.2.3 Determination of ctDNA levels

If ctDNA is detected, patients will undergo complete standard staging examinations. In case of M0, the patient will continue liquid-biopsy based ctDNA testing. In case of persisting ctDNA, a maximum of three consecutive negative staging examinations will be performed before staging intervals will be extended to every six months for another three consecutive scans. If ctDNA is persisting and patients show no radiological sign of recurrence, staging intervals will be extended to once a year (compare the complete workflow in Figure 6). If repeated, the staging examination will be performed via CT scan +/- a SPECT bone scan. If repeated, the minimum interval between two CT scans should be at least 3 months, and the minimum interval between two SPECT bone scans should be at least 6 months at any time point.
All staging examinations are done according the national guidelines for breast cancer.
Peripheral blood specimens will be collected in two Streck Cell-Free DNA BCT® tubes. ctDNA will be stable for up to 14 days at 6 °C to 37 °C. Presence of ctDNA will be analyzed centrally at Inivata Inc. using the RaDaR™ assay. Therefore, primary tumor tissue and peripheral blood specimens will be shipped for centralized analysis to Inivata Inc. RaDaR™ is a tumor-informed approach, beginning with whole exome sequencing (WES) of a tumor specimen from a patient's biopsy or surgical resection.

Somatic variants [single nucleotide variants (SNVs) and small insertions and deletions (indels)] identified from the exome sequencing are prioritized to build a patient specific primer panel of up to 48 tumor-specific somatic variants. This design is unique to each patient and is used as a molecular signature that can be tracked from diagnosis, in order to monitor presence of tumor DNA throughout the course of the patient's disease. Patient specific primers are combined with common Single-Nucleotide Polymorphism (SNP) primers for Next Generation Sequencing (NGS) for quality control purposes. To detect patient specific ctDNA, NGS testing is performed with the RaDaR™ assay using a multiplex PCR based on the InVision® platform (57,58).

Primary tumor tissue is mandatory for WES (FFPE Slide (≥ 6 mm³, min. 10 slides, thickness: 5 µm-10 µm, area >150 mm² and 1 H&E-stained slide, minimum 20% tumor content) or FFPE Block (≥ 6 mm³ thickness: 100 µm, area: >150 mm² and 1 H&E-stained slide, minimum 20% tumor content) or Genomic DNA extracted from FFPE slides or block (≥ 600 ng, Minimum volume: 25 µL, concentration: 20 ng/µL, buffer: 10 mM Tris pH 8, 1 mM EDTA)). If this cannot be provided, the patient will be accounted for as a screening failure.

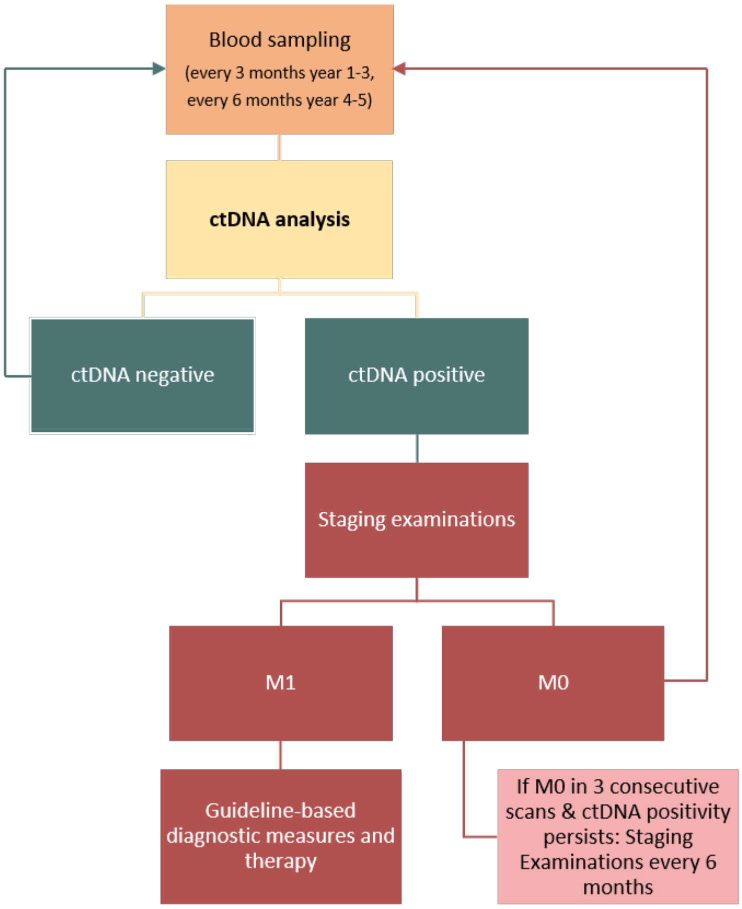

Figure 6: Pathway for ctDNA Analysis

12.2.4 Overview of Requirements for Collection of Biomaterials

Details for collection and handling of biomaterials are outlined in the Lab Manual/Sample Management Guide and in Table 4.

12.2.5 Blinding

This study will be performed as partially double-blinded. This means, all patients and doctors are blinded initially as blood sampling is done in all patients, irrespective of randomization to the Standard Surveillance arm or the Intensive Surveillance arm. If one of the biomarkers (CA27.29, CEA, CA125, CTC, ctDNA) is abnormal and triggers imaging, unblinding is the consequence as these patients will be asked to undergo further assessments and the responsible doctor will arrange these. Unblinding in this case is the ethical consequence of not letting all patients undergo additional imaging without elevated biomarkers. If no imaging is necessary, patients in the Intensive Surveillance arm will not be unblinded as there is no purpose to serve. Patients in the Standard Surveillance arm will not be unblinded altogether.

12.3 Staging Examinations

Staging will be triggered by symptoms of metastases as indicated by physician's choice (i.e., pathological fractures and associated symptoms, icterus, hepatic capsule pain, dyspnea, etc.) in both arms, as intended by current standard of care. Moreover, in the **Intensive Surveillance** arm staging will be triggered by biomarker findings indicating MRD as defined in 12.2.1, 12.2.2 and 12.2.3.
Staging examinations should as a minimum include a CT scan of the chest and abdomen as well as a SPECT bone scan. In cases of consecutive negative staging examinations or severe allergy to the contrast agent used for the CT scan, a PET scan (Positron emission tomography) or whole-body MRI may be considered as well and present a suitable addition. Additional body imaging like sonography can be used for further examination but should not replace a CT scan.
The minimum interval between two CT scans should be at least 3 months, and two SPECT bone scans should be at least 6 months apart at any time point.
The examinations will be performed in the radiological practice or hospital of the patients' choice. A medical radiological report with RECIST finding is not required.

12.4 Study visits

All study visits requiring blood sampling should be performed at the participating study centers. The standard aftercare examination with the recommended intervals of every three months for the first three years and every six months for the consecutive two years should take place at the patients' aftercare specialist (gynecologist/family physician/oncologist).

12.4.1 Study Inclusion / Baseline Visit

Patients should be thoroughly informed and need to give (written) consent to participate in the study. Furthermore the patient history, current health status and physical examination should be noted (see section 12.5.2). Eligible patients should show no clinical signs of metastatic disease.

At study inclusion, primary tumor tissue is required in form of a FFPE Slide, FFPE Block or Genomic DNA extracted from FFPE slides or block for sending to Inivata Inc. to establish the patient-specific RaDaR™ assay for later ctDNA testing. Tumor tissue of both sides will be needed in case of bilateral breast cancer. As the study is blinded, tissue is required from all patients in both arms. After randomization, tissue not used for establishing the RaDaR™ will be stored in a biobank. If a personalized ctDNA assay cannot be established for a patient in the Intensive Surveillance arm due to problems related to quality and/or quantity of the collected primary tumor tissue, a second primary tumor tissue sample from this patient will be obtained to facilitate another try at establishing a personalized ctDNA assay. If it still is not possible, this already randomized patient will be accounted for as drop-out.

Baseline tumor marker blood sampling of whole blood in 1 x 7,5 mL Serum tube will be done at randomization (Baseline visit 1) and 28 ± 5 days later (Baseline visit 2). Baseline ctDNA blood sampling of whole blood in 2 x 10 mL Streck Cell-Free DNA BCT® tubes will be done at randomization (Baseline visit 1).

Again, in order to maintain a double-blinded approach, biomarkers will only be analyzed in the Intensive Surveillance arm, while the biomaterial from the Standard Surveillance arm will be stored in a biobank.

Besides blood sampling, patients in both arms will get two questionnaires to assess their current QoL (EORTC QLQ-C30 and PA-F12).

12.4.2 Visits During Interventional Part of the Study

In the five years of interventional study participation, blood sampling will be done every three months for the first three years and every six months for the following two years after randomization in both arms. Blood sampling for all patients will contain whole blood in one 7,5 mL Serum tube as well as two 10 mL Streck Cell-Free DNA BCT® tubes in both arms (total of 27,5 mL whole blood). After one year, all patients will have an additional blood sampling of three 10 mL CellSave tubes (at study center visit for month 12 – a total of 57,5 mL whole blood).

Biomarkers will only be analyzed in the Intensive Surveillance arm, the biomaterial from the Standard Surveillance arm will be stored in a biobank.

If tumor marker, CTC or ctDNA testing is abnormal in the Intensive Surveillance arm, the study center will be notified to initiate a complete staging (compare to section 12.3).

Besides blood sampling, patient's QoL will be assessed in both arms with two questionnaires (EORTC QLQ-C30 and PA-F12) every six months. Furthermore, patients will be asked questions addressing examinations outside this study.

12.4.3 End of Intervention (EOI)

At the end of intervention, patients’ current health status should be noted.
If the detection of recurrence is the reason for the end of intervention, the site of recurrence, the site of metastases and – if possible – the planned therapy should be noted. In this case, biomaterial collection and submission are highly endorsed. If possible, tumor tissue in form of a FFPE Slide, FFPE Block or Genomic DNA extracted from FFPE slides or block for sending to Inivata Inc. would be appreciated.

One last blood sampling of whole blood in form of one 7,5 mL Serum tube as well as two 10 mL Streck Cell-Free DNA BCT® tubes, (± 3 x 10 mL CellSave tubes, if applicable, see section 12.2.2) is mandatory. Besides blood sampling, patients in both arms will get two questionnaires to assess their current QoL (EORTC QLQ-C30 and PA-F12).
If the end of the follow-up visits is the reason for the end of intervention, the EOI corresponds to Visit 16 (see Figure 8).

12.5 Study Procedures

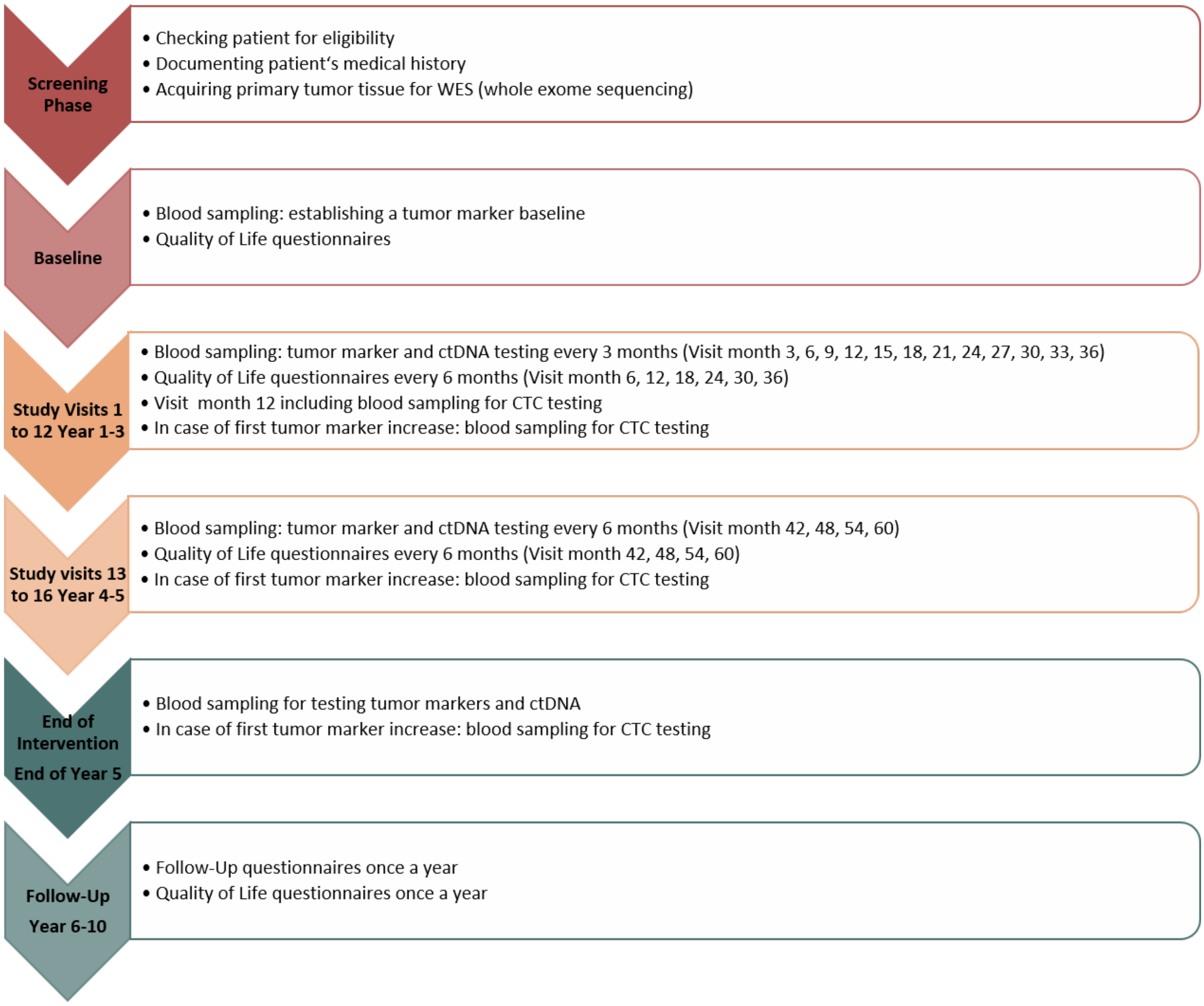

Figure 7: Study Procedures

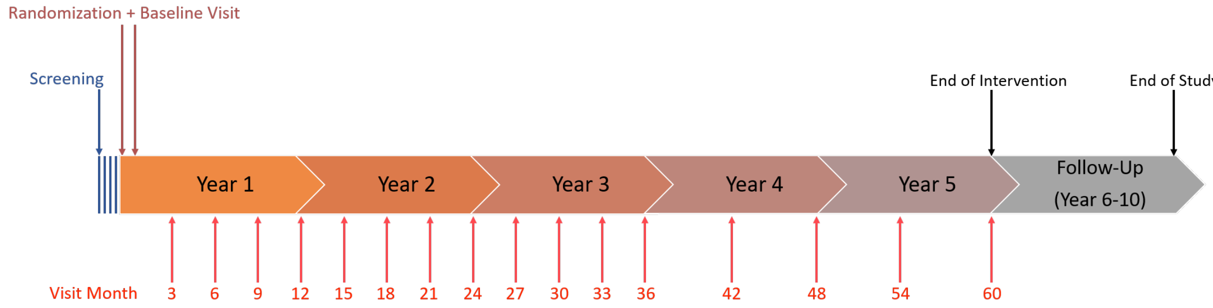

Figure 8: Timeline for Study Procedures during the interventional Phase

Every effort should be made to ensure that protocol required tests and procedures are performed as described.

Blood sampling and performing questionnaire will be done as seen in the flow chart (see Figure 7). The whole study timeline can be observed in Figure 9.

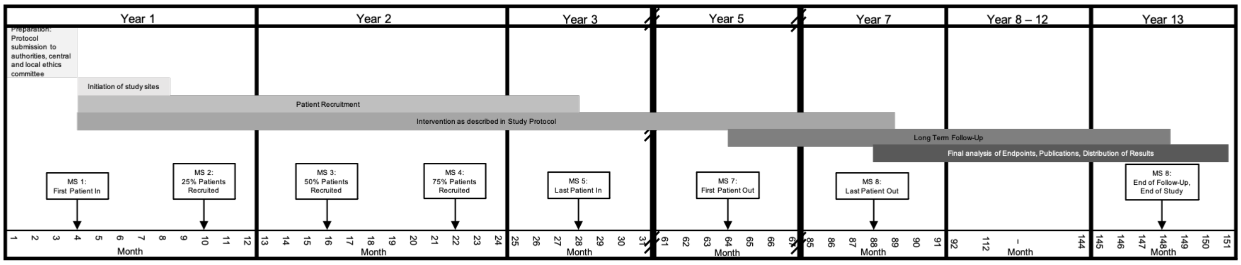

Figure 9: Study Timeline

To ensure standardization of biomarker testing, all tubes of blood samples will be sent from the study centers to the Central Study Center at University Hospital Ulm where the respective tubes will be distributed to the corresponding laboratories. All tumor marker and CTC testing will take place centrally with standardized assays. Blood for ctDNA-testing will be sent to Inivata Inc. for further analysis.

Unused and excess biomaterial will be stored in a biobank.

In case of an abnormal biomarker result, the Central Study Center will be informed by the corresponding institution. The Central Study Center will then inform the patient's respective study center which measures are to be taken (see Figure 10 + Figure 11).

If imaging is triggered, staging examinations (see section 12.3) will be performed in the radiological practice or hospital of the patients' choice as we trust in the professional knowledge of trained doctors to find metastases. A medical radiological report with RECIST finding is not required.

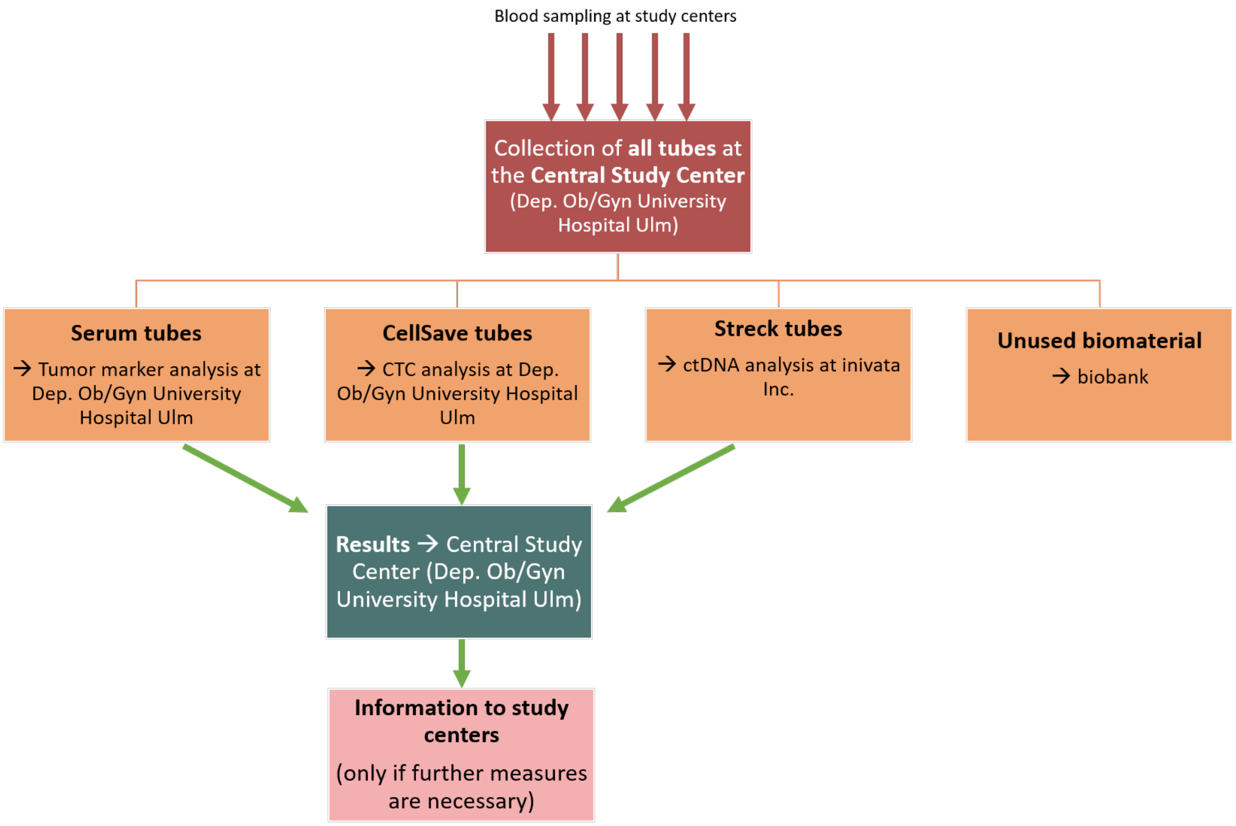

Figure 10: Processing of Biomaterial

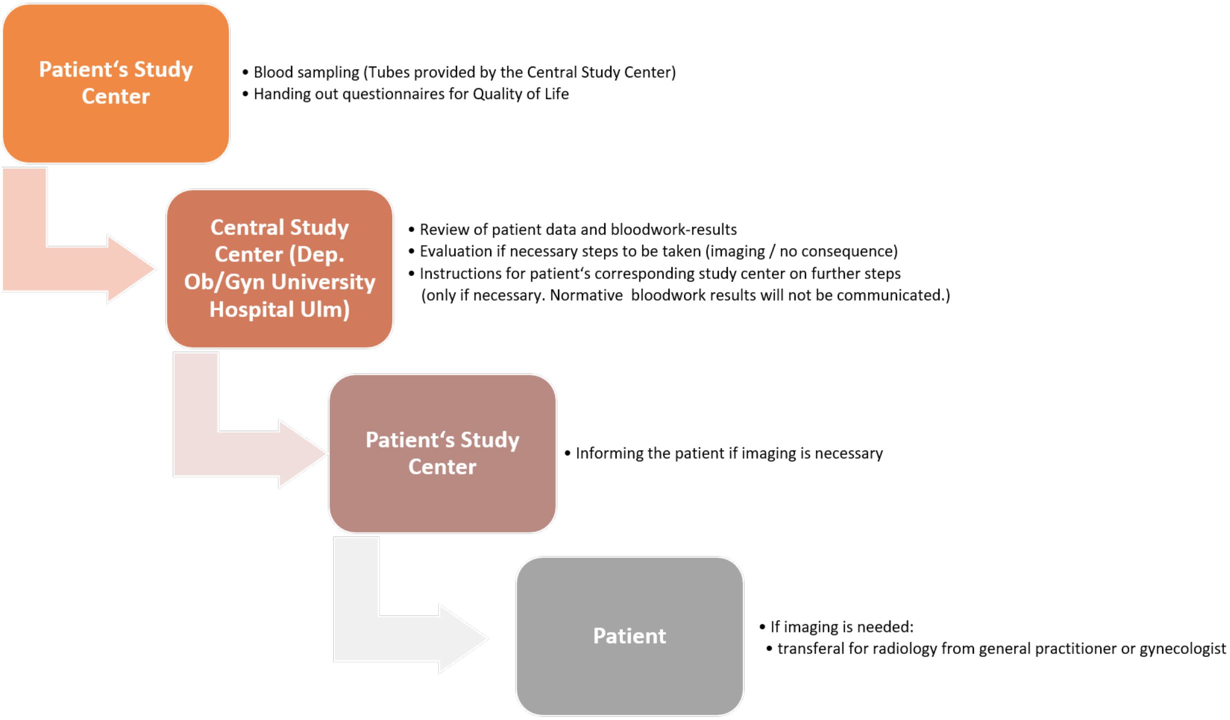

Figure 11: Responsibilities in Case of Positive Findings in Biomarkers

12.5.1 Patient Entry Procedures

12.5.1.1 Patient Consent Form and Screening

Prior to study entry, the consent form including any addenda must be signed and dated by the patient and the person obtaining informed consent. After written informed consent has been obtained, the patient has to be registered via an electronic data capturing (EDC) system (see section 12.5.1.2). The study site will obtain a unique patient number which will stay the same throughout the entire study covering all study periods. At this time point, the patient is enrolled into the study.

After a patient has completed the necessary screening visit procedures, the corresponding baseline case report forms (CRFs) have to be completed by the patient's study center using the EDC system. Eligibility of the patient will be automatically and visually checked in the EDC system.

It is possible for patients to participate in adjuvant interventional breast cancer trials and in interventional trials that are based on MRD detection without metastases being confirmed via imaging. These interventional trials should be defined therapy-intervention trials that are evidence-based and have to be approved by the SURVIVE Steering Committee.

12.5.1.2 Randomization and Stratification

Once eligibility has been established and the primary tumor tissue has been obtained, patients will be randomized in the EDC system in a 1:1 ratio, and the site will obtain the confirmation of the patient's randomization (without knowledge of the study arm) from the EDC system. Neither the investigators/co-investigators nor the patient can influence the randomization. A partially double-blinded study design will be used, where only patients in the Intensive Surveillance arm with detection of MRD resulting in imaging or additional blood sampling will be informed about their randomization arm. Correspondingly, the patient's study center will also be informed about the randomization arm in case of positive biomarker findings as they will be the link between the Central Study Center and the patient and have to organize required imaging and/or additional blood sampling.
Patients will be randomized centrally only if they fulfil the inclusion criteria and are not precluded from participation by any of the exclusion criteria. The randomization list will be generated by the IFG GmbH Erlangen using adequate and validated algorithms.

Randomization will be stratified using block randomization by:

- Hormone receptor status (negative vs positive)
- HER2 status (negative vs positive)
- Histological lymph node status at surgery ((y)pN0 vs (y)pN+)

12.5.2 Screening

Table 1: Screening exams and tests prior randomization

	**Investigations at Screening[a]**	**Timing (prior to randomization)**
Patient informed consent	Obtained yes/no	Prior to any protocol investigations or procedures
History and physical examination	History including:<br>• Gender / Sex<br>• ethnicity<br>• diagnosis of breast cancer<br>• menopausal status<br>• therapy history of breast cancer<br>• current aftercare status<br>• family history of cancer<br>• known genomic mutation<br>• general medical history including cardiac history and allergy<br>• concurrent illnesses<br>• concomitant medications (any prescription medications or over-the-counter medications),	Within 84 days prior to randomization

	Investigations at Screening[a]	Timing (prior to randomization)
	and their indication, used within one month prior to study entry<br>• current pregnancy<br><br>Physical examination including:<br><br>• ECOG performance status<br>• Weight<br>• Height<br>• Palpation of breasts, axillary, supra- and infraclavicular region<br>• Pre-existing signs and symptoms as in no clinical signs of distant metastases	
Biomaterials[b]	Primary tumor tissue as either of the following:<br><br>• FFPE Slide (≥ 6 mm³, min. 10 slides, thickness: 5 µm-10 µm, area >150 mm² and 1 H&E stained slide, minimum 20 % tumor content) or<br>• FFPE Block (≥ 6 mm³ thickness: 100 µm, area: >150 mm² and 1 H&E stained slide, minimum 20 % tumor content) or<br>• Genomic DNA extracted from FFPE slides or block (≥ 600 ng, minimum volume: 25 µL, concentration: 20 ng/µL, buffer: 10 mM Tris pH 8, 1 mM EDTA)	After ICF signature
	20 mL whole blood (2 x 10 mL Streck tubes)<br>7,5 mL whole blood (1 Serum tube)	baseline visit 1 (randomization visit)
	7,5 mL whole blood (1 Serum tube)	baseline visit 2: 28 days after baseline visit 1 (± 5 days)
Biochemistry	Serum creatinine and resulting renal clearance	Within 84 days prior to randomization
Breast Imaging[c]	Bilateral mammography and supplementary bilateral breast ultrasound with axillary lymph nodes (where applicable)<br>and/or MRI of the breasts	Within 1 year prior to randomization

	Investigations at Screening[a]	Timing (prior to randomization)
a. Results of screening tests or examinations performed as standard of care prior to obtaining informed consent but within the timelines may be used rather than repeating required test.<br>b. Materials for blood collection and transportation devices will be provided by the Central Study Center at Dep. Ob/Gyn University Hospital Ulm.<br>c. Examination is performed externally, no study physician needed.		

12.5.3 Evaluation during Interventional Phase

The investigator may schedule visits (unplanned visits) in addition to those listed on the schedule of activities, in order to conduct evaluations or assessments required to protect the wellbeing of the patient.
Patients who stop study interventions due to breast cancer recurrence (distant or local), second primary breast cancer, or diagnosis of another cancer or who discontinue all protocol requirements for reasons other than disease recurrence must complete the end of intervention (EOI) assessments. In case of recurrence or other primary malignancy, biomaterial collection and submission are strongly endorsed.

Table 2: Requirements during Interventional Phase

	Evaluation during Interventional Phase	Timing
History[d]	• Last aftercare visits at the aftercare specialist (gynecologist/family physician/oncologist).<br>• Current adjuvant therapy regarding breast cancer.<br>• Last breast imaging (mammography/ ultrasound)<br>• Any test for tumor markers or imaging done outside of study.	Every 3 months (year 1–3)<br><br>Every 6 months (year 4–5)<br><br>EOI
Breast Cancer Aftercare[e]	• Contents of interview depending on the external aftercare specialist's (gynecologist/family physician/oncologist) approach<br>• Breast examination with palpation of breasts and locoregional lymph nodes<br>• Laboratory tests as needed<br>• Initiation/Scheduling of yearly breast imaging	Every 3 months (year 1–3)<br><br>Every 6 months (year 4–5)
Breast Imaging[e]	Bilateral mammography and supplementary bilateral breast ultrasound with axillary lymph nodes (where applicable)<br>and/or MRI of the breasts	Once a year

	Evaluation during Interventional Phase	Timing
Biomaterials[a, b]	7,5 mL whole blood (1 Serum tube)<br><br>20 mL whole blood (2 x 10 mL Streck tubes)	Every 3 months<br>(year 1–3)<br><br>Every 6 months<br>(year 4–5)<br><br>EOI
	30 mL whole blood (3 x 10 mL CellSave tubes)	Visit month 12<br><br>In case of first abnormal increase in tumor marker[c]
Questionnaires[d]	EORTC QLQ-C30<br>PA-F12	Every 6 months (year 1-5)
Imaging tests for staging[e]	• CT scan of chest and abdomen<br>• SPECT bone scan<br>• If not possible due to e.g., allergy to the contrast agent: PET scan or whole-body MRI<br>• Complementary imaging tests if indicated	• Symptomatic patients: physician's choice<br>• Asymptomatic patients: only if center is advised to
a. Materials for blood collection and transportation devices will be provided by the Central Study Center at Dep. Ob/Gyn University Hospital Ulm.<br>b. The corresponding blood kit will be sent to the study center by the Central Study Center.<br>c. Only if CTC assessment was not done at regular visit.<br>d. Done by questionnaire.<br>e. Examination is performed externally, no study physician needed.		

12.5.4 Evaluation after End of Intervention - Follow-up

The investigator may schedule visits (unplanned visits) in addition to those listed on the schedule of activities, in order to conduct evaluations or assessments required to protect the wellbeing of the patient.
Patients who discontinue all protocol interventions for reasons other than disease recurrence must follow the exams and assessments outlined in this section.

Follow-up data will be collected via questionnaires which will be provided in paper form or digitally (via E-Mail / Digital Health Application or collected via telephone interview.

Table 3: Evaluation during Follow-up

	Evaluation during Follow-up	Timing
History[b]	• Aftercare visits at the aftercare specialist (gynecologist/family physician/oncologist).<br>• Current status of breast cancer (recurrence?)<br>• Current (adjuvant) therapy regarding breast cancer<br>• Last breast imaging (mammography/ultrasound)<br>• Any test for tumor markers or imaging done	Once a year (year 6-10)
Breast Cancer Aftercare[a]	• Contents of interview depending on the external aftercare specialist's (gynecologist/family physician/oncologist) approach<br>• Breast examination with palpation of breasts and locoregional lymph nodes<br>• Laboratory tests as needed<br>• Initiation of yearly breast imaging	At least once a year (year 6-10)
Breast Imaging[a]	Bilateral mammography and supplementary bilateral breast ultrasound with axillary lymph nodes (where applicable)<br>and/or MRI of the breasts	Once a year (year 6-10)
Questionnaires[b]	EORTC QLQ-C30<br>PA-F12	Once a year (year 6-10)
a. Examination is performed externally, no study physician needed.<br>b. Done by questionnaire.		

Patients who stop study therapy due to breast cancer progression/relapse, second breast primary cancer, or diagnosis of another cancer or who discontinue all protocol requirements for reasons other than disease recurrence must complete the end of intervention (EOI) assessments.
Follow-up visits after the EOI are once yearly and include no biomaterial sampling.
In case of recurrence or other primary malignancy, biomaterial collections in form of blood sampling and tumor tissue are endorsed.

12.5.5 Long-Term Follow-up After End of Study

In terms of long-term follow-up after end of study, patients have the possibility to participate in the patient self-reporting registry (Patientenselbstauskunft)

12.5.6 Study visits table

	Trial Period	Screening[a]	Randomisation visit	Baseline visit	Surveillance Phase																End of Intervention	Follow Up	End of Study
			Baseline 1	Baseline 2	Year 1-3												Year 4-5				Month 60 or premature discontinuation[b]	Year 6-10	
	Visit schedule	Prior to randomisation	Tumorblock in central pathology	28 d post randomisation	Every 3 months												Every 6 months					Every 12 months	
	Day/Month	-84 d to 0 d	0	28 d ± 5 d	3	6	9	12	15	18	21	24	27	30	33	36	42	48	54	60		72 to 120	120
**APPLICABLE FOR BOTH ARMS**																							
**Registration requirements**	Informed Consent Form signed	X																					
	Inclusion/Exclusion criteria evaluation	X																					
	Medical history	X																					
	Documentation Disease Baseline Characteristics	X																					
	Physical examination	X																					
	Primary archived (FFPE) tumor tissue[c]	X																					
**Biochemistry[d]**	Serum creatinine and renal clearance	X																					
**Biomaterials[e]**	Tumormarkers 1x 7.5 mL serum tube		X	X	X	X	X	X	X	X	X	X	X	X	X	X	X	X	X	X	X		
	ctDNA 2x 10 mL Streck tubes		X		X	X	X	X	X	X	X	X	X	X	X	X	X	X	X	X	X		
	CTCs[f] 3x 10 mL cell save tubes							X															
	Archived (FFPE) Tumor tissue upon progression[g]																				X		

	Trial Period	Screening[a]	Randomisation visit	Baseline visit	Surveillance Phase																End of Intervention	Follow Up	End of Study
			Baseline 1	Baseline 2	Year 1-3												Year 4-5				Month 60 or premature discontinuation[b]	Year 6-10	
	Visit schedule	Prior to randomisation	Tumorblock in central pathology	28 d post randomisation	Every 3 months												Every 6 months					Every 12 months	
	Day/Month	-84 d to 0 d	0	28 d ± 5 d	3	6	9	12	15	18	21	24	27	30	33	36	42	48	54	60		72 to 120	120
	**Trial Period**	**Screening[a]**	**Randomisation visit**	**Baseline visit**	**Surveillance Phase**																**End of Intervention**	**Follow Up**	**End of Study**
			Baseline 1	Baseline 2	Year 1-3												Year 4-5				Month 60 or premature discontinuation[b]	Year 6-10	
	Visit schedule	Prior to randomisation	Tumorblock in central pathology	28 d post randomisation	Every 3 months												Every 6 months					Every 12 months	
	Day/Month	-84 d to 0 d	0	28 d ± 5 d	3	6	9	12	15	18	21	24	27	30	33	36	42	48	54	60		72 to 120	120
**APPLICABLE FOR BOTH ARMS**																							
**Staging examinations[h,i]**	CT scan of chest and abdomen and SPECT bone scan				X	X	X	X	X	X	X	X	X	X	X	X	X	X	X	X	X		
**Questionnaires[j]**	EORTC QLQ-C30 (QOL)		X			X		X		X		X		X		X	X	X	X	X	X		
	PA-F12 (QOL)		X			X		X		X		X		X		X	X	X	X	X	X		
	History				X	X	X	X	X	X	X	X	X	X	X	X	X	X	X	X	X		
	Follow-up																					X	

a: Results of screening tests or examinations performed as standard of care prior to obtaining informed consent but within the timelines may be used rather than repeating required test

b: Premature study intervention discontinuation due to either of the following:
1. via imaging verified recurrence
2. pregnancy
3. safety or medical reasons
4. patients' withdrawal of consent

c: Primary tumor tissue according to the requirements stated in the study protocol section 11.2. Important note: in case of bilateral breast cancer, tissue from both sides is needed.

d: Results of serum creatinine and renal clearance performed within 84 days prior to obtaining informed consent may be used rather than repeating required test

e: Materials for blood collection and transportation devices will be provided by the Central Study Center at Dep. Ob/Gyn University Hospital Ulm

f: Mandatory at Month 12, additionally required at visit after first verified tumormarker increase, whenever that occurs

g: Highly endorsed in case of detection of recurrence/metastatic disease

h: Referral to radiologist from investigator or aftercare specialist (gynecologist/family physician/oncologist) - Can be triggered by either of the following:
1. clinical indication (both arms)

2. increase in tumormarkers (only intensive surveillance arm)
3. if presence of ctDNA was detected (only intensive surveillance arm)

i: If CT and SPECT not possible due to e.g., allergy to the contrast agent: PET scan or whole-body MRI, Complementary imaging tests if indicated

j: Available on paper, handed out at study visits

13 COLLECTION OF BIOMATERIALS AND TRANSLATIONAL RESEARCH

By signing the informed consent form, the patient agrees to collection and submission of required sample collections as outlined below. The tumor and blood samples will be used for testing at the institutions stated and/or translational research as described in the protocol.
Material (i.e., test tubes) for blood collection and transportation devices in the study centers will be provided by the Central Study Center.

13.1 Overview of Requirements for Collection of Biomaterials

Details for collection and handling of biomaterial are outlined in the Lab Manual/Sample Management Guide and in the table below.

Table 4: Schedule of biomaterial collection

Biomaterial	Screening Phase		Surveillance Phase	
	**Before randomization**	**Baseline** (after randomization)	**Year 1-3** (every 3 months) **Year 4-5** (every 6 months) **EOI**	**Visit month 12**
**Primary Tumor Tissue**	**X**[a]			
**Whole Blood (7,5 mL Serum)**		**X**[b]	**X**	
**Whole Blood (3 x 10 mL CellSave)**			**X**[c]	**X**
**Whole Blood (2 x 10 mL Streck)**		**X**[d]	**X**	

a. FFPE Slide, FFPE Block or Genomic DNA extracted from FFPE slides or block (technical requirement for establishing ctDNA testing). Tumor tissue of both sides will be needed in case of bilateral breast cancer.
b. Two blood samples with four weeks apart
c. In case of first abnormal tumor marker increase
d. At Randomization visit/Baseline visit 1 only

13.1.1 Tumor Material

13.1.1.1 Mandatory tumor material

Primary tumor tissue in form of a FFPE or a FFPE Block or genomic DNA extracted from FFPE slides or block will be shipped for centralized analysis to Inivata Inc. for the establishment of the patient-specific RaDaR™ assay that is necessary for further detection of ctDNA during the time of the study. In case of bilateral breast cancer, tumor tissue of both sides is mandatory. For establishing this assay, whole exome sequencing (WES) of the patient's tumor tissue is performed. Somatic variants [single nucleotide variants (SNVs) and small insertions and deletions (indels)] identified from the exome sequencing are prioritized to build a patient specific primer panel of up to 48 tumor-specific somatic variants. This design is unique to each patient and is used as a molecular signature that can be tracked from diagnosis, in order to monitor presence of tumor DNA throughout the course of the patient's disease. Patient specific primers are combined with common Single-Nucleotide Polymorphism (SNP) primers for Next Generation Sequencing (NGS) for quality control purposes. This creation process is only necessary at the beginning of the study as the assay can then be used for the whole duration of the study.
Tumor tissue which is not used will be stored in a biobank.

13.1.1.2 Optional tumor material

In case of local or distant recurrence or in case of a second primary malignancy, biomaterial collection and submission is highly endorsed. Biomaterial in form of biopsy of tumor or metastasis would be appreciated (FFPE or a FFPE Block or genomic DNA extracted from FFPE slides or block).

13.1.2 Blood Samples

Blood sampling will be performed by the established standard of care in the corresponding study centers and the biomaterial will then be sent to the Central Study Center for further distribution to the respective institutions who will perform the further analysis specified in this study protocol (tumor markers, CTC and ctDNA). Excess blood samples and blood samples not used for analysis will be stored in a biobank.

13.1.2.1 Mandatory blood samples for all patients

All patients in both arms will receive blood sampling every three months for the first three years and every six months for the following two years after randomization. The sampling will at least include an amount of 27,5 mL whole blood (1 x 7,5 mL Serum and 2 x 10 mL Streck) for regular visits for patients in both arms. As a one-time event, at Visit month 12 (after one year of study participation), an extra 30 mL whole blood will be taken for CTC-testing (3 x 10 mL CellSave) in the intensive surveillance arm.
In the Intensive Surveillance arm, an extra 30 mL whole blood (3 x 10 mL CellSave) is required if a tumor marker increase is detected for the first time and if no CTC testing was done at the regular visit.

13.1.2.1.1 Tumor Marker Analysis

As no cut-offs but a delta in increase from baseline (CA27.29 +75% or CEA +100% or CA125 +150%) will be used to determine significant tumor marker elevation, two blood samplings for tumor marker testing will be performed in a 28 ± 5 days interval to establish a baseline. The baseline will be defined as the mean tumor marker value of two blood drawings four weeks apart (28 ± 5 days) at the timepoint of randomization for each patient individually. After determination of the baseline, blood samples will be drawn in the intervals described above.

Depending on the result of this blood test and the result of the imaging, if necessary, planned intensive surveillance will be continued or not (for details see section 12.2.1).

CA27.29, CEA and CA125 will be measured with the AIA®-CL1200 by TOSOH BIOSCIENCE (TOSOH CORPORATION, Tokyo, Japan). CA125 corresponds to OVCA TEST CUP. Specimen will be collected in one standard serum sampling tube (7,5 mL whole blood), and shipped for centralized evaluation. Serum probes will be stable for 48h at 20-25°C, for 7 days at 2-8°C and thereafter at -20°C. For each analysis, the sample volume needed is 5, 10 and 20 µL respectively for CA27.29, CEA and OVCA and up to 120 results per hour may be generated.

The CL AIA-PACK CA27.29/CEA/OVCA assays are two-step chemiluminescence enzyme immunoassay (CLEIA) kits. The assays are performed in the CL AIA-PACK CA27.29/CEA/OVCA TEST CUP respectively. CA27.29/CEA/CA125 present in a test sample is bound to anti- CA27.29/CEA/CA125 mouse monoclonal antibody immobilized on the magnetic microparticles in one cell (Cell-I). After first incubation, the magnetic microparticles are washed to remove unbound materials and then a specific volume of the enzyme-labeled anti- CA27.29/CEA/CA125 mouse monoclonal antibody that has been reconstituted in another cell (Cell-II) is dispensed into Cell-I. After second incubation, the magnetic microparticles are washed again to remove unbound enzyme-labeled monoclonal antibody and are incubated with a chemiluminescent substrate, DIFURAT® (3-(5-*tert*-Butyl-4,4-dimethyl-2,6,7-trioxabicyclo[3.2.0]hept-1-yl) phenylphosphate disodium salt). The amount of enzyme-labeled antibodies that bind to the magnetic microparticles is directly proportional to the CA27.29/CEA/CA125 concentration in the test sample. A standard curve is constructed, unknown sample concentrations are calculated by using this curve.

Principles of analysis and probe handling is explained detailed in the according leaflets (56).

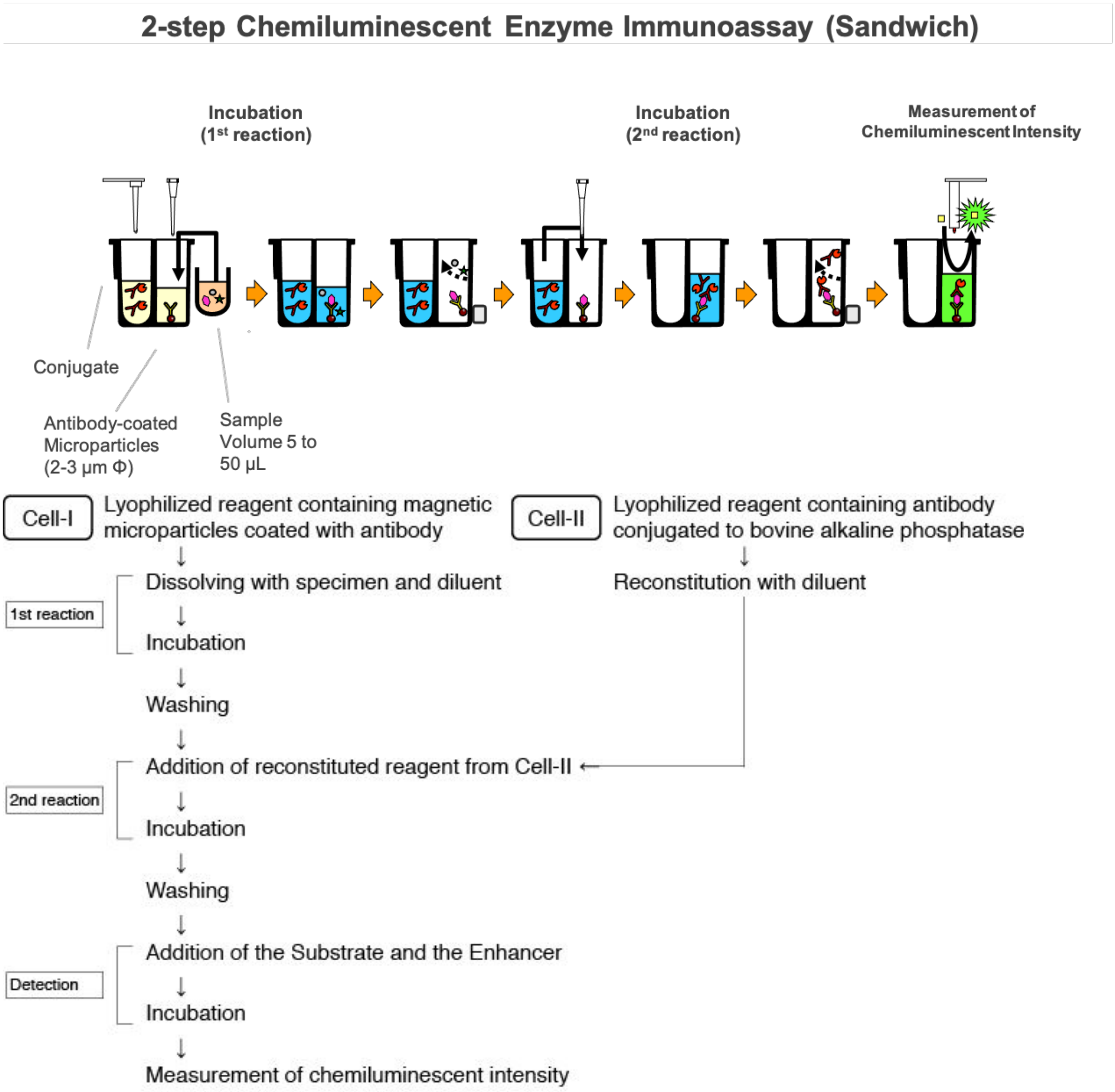

Figure 12: Principle of the CA27.29, CEA and CA125 (OVCA) Analysis Workflow (provided by Tosoh Bioscience)

13.1.2.1.2 CTC Analysis

If an abnormal tumor marker value is detected for the first time, CTC analysis will be initiated. Furthermore, all patients in the Intensive Surveillance arm will receive CTC testing one year after study-enrollment.

Whole blood samples will be collected in 3 x 10 mL CellSave preservative tubes at the study centers at specific timepoints and transported with the other biomaterial to the Central Study Center.

CTCs in CellSave tubes will be stable for 96 h at 20-25°C.
CTCs will be analyzed using the FDA-approved, standardized semiautomatic CellSearch® System by Menarini Silicon Biosystems.
The CellSearch® system is designed to enumerate CTCs of epithelial origin (CD45-, EpCAM+, cytokeratin 8+ / 18+ and/or 19+)(59). The basic principle is linking a magnetic ferrofluid reagent that contains i. a. antibodies targeting the EpCAM antigen to CTCs. After steps of immunomagnetic capture and enrichment as well as addition of fluorescent reagents (that contain anti-CK-PE, DAPI and anti-CD45-APC), the automatic dispersion to a magnetic cartridge holder takes place. Via strong magnetic field, the magnetically labeled epithelial cells are attracted to the surface of the cartridge where they can be scanned automatically.
Images of events where CK-PE and DAPI fluorescence are co-located are presented to the user for final classification. An event is classified as a tumor cell when its morphological features are consistent with that of a tumor cell and it exhibits the phenotype EpCAM+, CK+, DAPI+ and CD45- (59).

13.1.2.1.3 ctDNA Analysis

ctDNA will be measured every three months for three years, thereafter every six months for another two years. Peripheral blood specimens will be collected in two Streck Cell-Free DNA BCT® tubes by the study centers and transported with the other biomaterial to the Central Study Center. At this point, distribution to Inivata Inc. will take place for further analysis. ctDNA will be stable for up to 14 days at 6 °C to 37 °C. Depending on the result of the test and the result of the imaging, further controls may be necessary in the regular interval (for details see section 12.2.3).
Presence of ctDNA will be analyzed using the personified RaDaR™ assay consisting of the patient specific-primer panel that was designed with the primary tumor tissue send at baseline.
Plasma cell- free DNA and buffy coat DNA will be acquired from the whole blood sample.
To detect patient specific ctDNA, NGS testing is performed with the RaDaR™ assay using a multiplex PCR based on the InVision® platform (57,58).

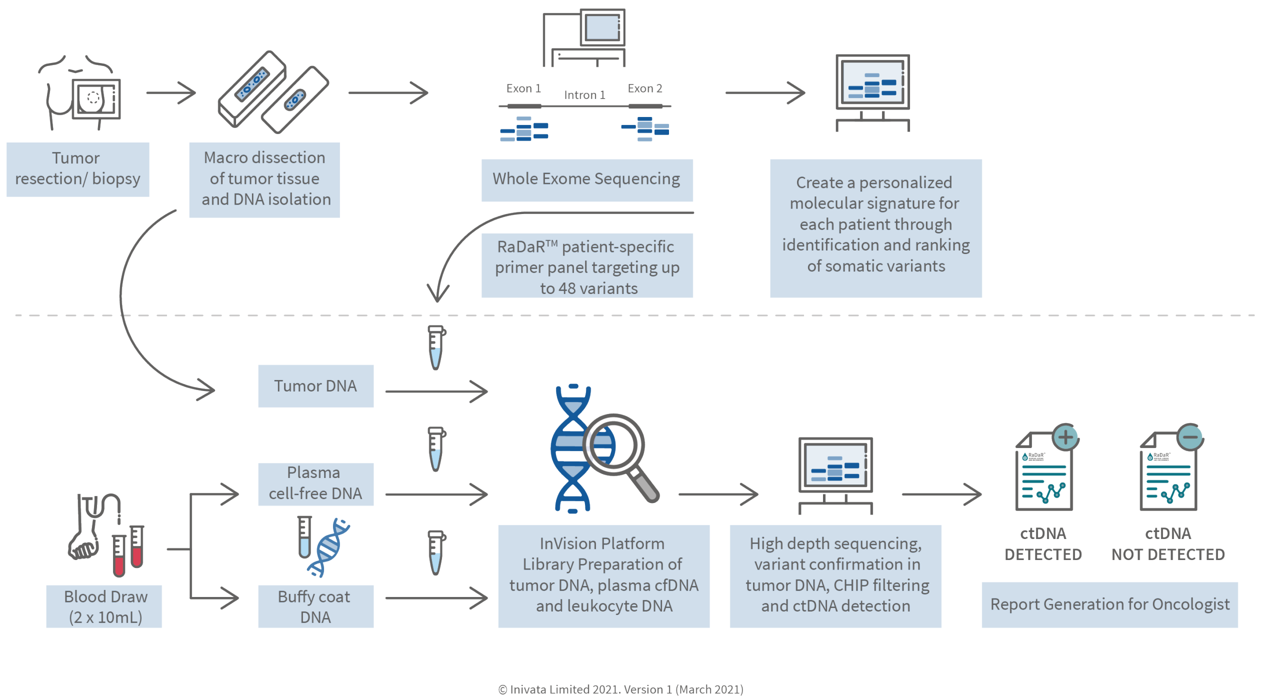

Figure 13: Principle of the RaDaR™ Workflow (provided by Inivata Ltd.)

13.1.2.2 Optional blood samples

Optional blood samples in form of 57,5 mL whole blood (1 x 7,5 mL Serum, 3 x 10 mL CellSave, 2 x 10 mL Streck) would be highly endorsed when breast cancer recurrence is observed. The blood samples will be sent to the Central Study Center for further distribution to the respective institutions who will perform further analysis.

14 DISCONTINUATION OF STUDY INTERVENTION, PATIENT, STUDY AND SITE

14.1 Patients without Intervention

This study is conducted according to the principle of intent-to-treat (ITT).
Patients who have been randomized in the study but withdrew their consent or are withdrawn by the investigator from the study prior to first blood sampling are to be included in the intent-to-treat analysis and will not be replaced. The reasons for not starting blood sampling are collected.
If a personalized ctDNA assay cannot be established for a patient in the Intensive Surveillance arm due to problems related to quality and/or quantity of the collected primary tumor tissue, a second primary tumor tissue sample from this patient will be obtained to facilitate another try at establishing a personalized ctDNA assay. If it still is not possible, this already randomized patient will be accounted for as drop-out.

14.2 Premature Intervention Discontinuation

Patients who have been randomized and have received study intervention and, for whatever reason, did not participate throughout the entire study are classified as premature intervention discontinuation.
Investigators must attempt to contact patients who fail to attend scheduled visits by telephone, letter, visit, etc. Reasons for discontinuation must be documented in the CRF and in the patient’s medical records.

14.3 Investigator-Initiated Discontinuation of Study Intervention

In addition to the conditions outlined in the protocol, the investigator may require a patient to discontinue study participation if one of the following occurs:

- The patient’s health deteriorates.
- The patient is unable to meet the study requirements.
- New information about the study interventions for breast cancer becomes available.
- Any medical condition that may jeopardize the patient’s safety if he or she continues receiving study intervention.

14.4 Patient-Initiated Study Discontinuation vs Withdrawal of Consent

If a patient chooses to have no further interaction regarding the study, reason of discontinuation should be documented. The Investigator should not urge the patient to withdraw their consent. If a patient wants to discontinue all study related activities and withdraws her consent actively, withdrawal of consent should be documented. Biomaterials which have already been collected will be kept at the biobank and results from analyzed blood samples will be analyzed in final analysis unless the patient disagrees.

14.5 Premature Termination of Study

The University Hospital Ulm as the sponsor has the right to close this study due to, but not limited to the following reasons:

- If risk-benefit ratio becomes unacceptable owing to, for example:
  - safety findings from this study
  - results of interim analysis
  - results of futility analysis
  - results of parallel/other clinical studies
- If the study conduct (e.g., recruitment rate; drop-out rate; data quality; protocol compliance) does not suggest a proper completion of the study within a reasonable and expected time frame

The Independent Data Monitoring Committee (IDMC) and the Protocol Board will provide advice. The decision to premature termination of the study is binding to all investigators of all study sites. Responsible ethics committees will be informed about the reason(s) and time of termination according to the applicable laws and regulations.
If the study is terminated prematurely, all investigators have to inform their patients and take care of appropriate follow-up and further treatment of patients.

14.6 Premature Termination of Study at a Particular Site

The University Hospital Ulm as the sponsor has the right to close this study at a particular study site which may be due but not limited to the following reasons:

- Non-compliance with the protocol, GCP and/or regulatory requirements
- Insufficient number of recruited patients
- False documentation in the CRF due to carelessness or deliberately
- Inadequate co-operation with University Hospital Ulm or its representatives
- The Investigator request to close of his/her study site

The reasons will be discussed with the investigator. If the study is prematurely terminated in a study site, the responsible investigators have to inform their patients and take care of appropriate follow-up and ensure further treatment. The responsible ethics committee will be informed about the reason and time of termination according to the applicable laws and regulations.

15 END OF INTERVENTION (EOI)

EOI is defined as last withdrawal and analysis of blood samples and - if staging is triggered - last staging examination (whichever comes last).
EOI is possible in different scenarios:

- The patient was diagnosed with local recurrence of breast cancer, breast cancer metastases or a secondary malignancy. Guideline-based diagnostics and treatments will take place outside of the study.
- The patient becomes pregnant. Care at an obstetrician is advised.
- Patient or study center discontinues intervention. Patient is willing to participate in follow-up phase.
- All scheduled visits have been accomplished (end of Year 5).
- Death of the patient.

Afterwards, patients will enter the follow-up phase which will last for five years and does not contain any interventions in form of blood sampling or staging examinations triggered by biomarkers.
Follow-up data will be collected once a year via Digital Health Application, via Telephone interviews or in written via post and include the assessments mentioned in section 12.5.4.

If a patient shows a relevant finding in any biomarker (CA27.29, CEA, CA125, CTC, ctDNA) at the end of intervention but the consecutive staging shows no evidence of disease, the result of the biomarker will be told to the patient’s study center so that further staging modalities can be decided per Investigator’s discretion.

16 END OF STUDY (EOS)

Planned end of study is the last follow-up visit of the last patient being in the 5-year follow-up phase.

After end of study, final analysis of endpoints, publications and distribution of results will take place if not already happened.

In terms of long-term follow-up after end of study, patients have the possibility to participate in the patient self-reporting registry (Patientenselbstauskunft).

17 ASSESSMENT OF OUTCOME

17.1 Evaluation of Local Recurrence

Breast examination with palpation including locoregional lymph nodes will be performed at each aftercare visit at the aftercare specialist (gynecologist/family physician/oncologist; every three months for the first three years of aftercare, every six months for the following two years). Additionally, patients should be advised to perform monthly self-palpation of the breasts and lymph nodes to detect any potential tumor. As such clinical examination cannot be 100% accurate and also benign breast tumors can be palpable, additional mammography and, where needed, additional breast ultrasound is done when a recurrence is suspected. If palpation is inconspicuous during aftercare, mammography and ultrasound of both breasts are advised once a year. If mammography and/or ultrasound is inconclusive, an additional MRI of the breasts may be necessary.
Clinical Examinations and performance of mammography and breast ultrasound are recorded in the eCRF at every scheduled visit. If a recurrence of local breast cancer is diagnosed, the study center has to be notified immediately and the study center in turn reports to the IFG on eCRF.

17.2 Evaluation of Distant Metastases

Distant metastases can be diagnosed with any imaging available (CT scan, MRI, SPECT bone scan, sonography, etc.). In some cases, these examinations may be initiated due to other causes like back pain or elevated liver enzymes with the accidental finding of metastases of breast cancer.
In symptomatic patients with suspected metastases, a CT scan of chest and abdomen as well as a SPECT bone scan is advised. If another type of imaging was performed before that does not depict the whole patient, these examinations should be performed additionally.
In patients randomized to the Intensive Surveillance arm, a complete staging examination with CT scan of chest and abdomen as well as a SPECT bone scan will also be initiated in asymptomatic patients when a significant increase of tumor markers from baseline (CA27.29 +75% or CEA +100% or CA125 +150%) is verified after confirmatory blood sampling or when CTCs or ctDNA are detected.
Staging examinations done outside of the study are recorded with the patient questionnaire completed at every scheduled visit. If distant metastases are diagnosed, the study center has to be notified immediately and the study center in turn reports to the IFG GmbH Erlangen on CRF.

17.3 Evaluation of Biomarkers

The biomarkers used in this study (CA27.29, CEA, CA125, CTC, ctDNA) are in part well-known and in part new as diagnostic measures. Tumor markers are in use for monitoring metastatic breast cancer patients, usually in the combination of CA27.29 (or other MUC1-epitope CA15.3) and CEA as recommended by the ASCO. Hints of the additional tumor marker CA125 as a source of information have been seen so that we added it in the combination of tumor markers used in this study (24). However, absolute levels of tumor markers are only an indirect hint at possible tumor spread and can be elevated due to other reasons (e.g., renal insufficiency) which is why the kinetics of these markers instead of absolute numbers are of importance. CTCs as well as ctDNA are evidence that tumor cells can be found in the peripheral blood and therefore are a more direct way of detecting tumor

recurrence. As no comparison between these markers has been made so far, we chose to examine all three types to identify the most reliable one.

One of our primary endpoints is the Overall Lead Time defined as molecular to via imaging verified recurrence lead time in addition to the DFS-difference between the two arms (see Figure 2). This will be analyzed for each marker separately and also - depending on the biomarker - in combination.
For the calculation of overall lead time, a positive biomarker result is defined as an abnormal finding for any of the biomarkers used (i.e., either CA27.29 or CA125 or CEA or CTC or ctDNA). However, to specifically evaluate the performance with regard to early detection of MRD for single biomarkers, overall lead time will also be calculated separately for the conventional tumor markers (CA27.29, CA125, CEA), for CTCs and for ctDNA. In addition to that, the secondary endpoint of Molecular to via Imaging verified Recurrence Lead Time in the Intensive Surveillance arm is analyzed to determine whether a lead time effect can be generated between molecular to via imaging verified recurrence through liquid biopsy.
For another measurement of efficacy of these biomarkers, we analyze as secondary endpoints specificity and sensitivity of all the markers individually and also in combination regarding the tumor markers. The reason for the latter is the evidence that the combination of tumor markers may show a higher sensitivity than seen individually (26). As an additional secondary objective, the False-Positive Liquid Biopsy Rate being defined as no clinical relapse 36 months after first and persistent molecular relapse shall be analyzed.
We expect the availability of reliable data from all patients shortly after the end of study.

18 ASSESSMENT OF HEALTH-RELATED QUALITY OF LIFE

18.1 Rationale

QoL may be influenced in different ways in both arms of the study as patients and doctors are blinded with the exception of positive findings in the Intensive Surveillance arm.
In the Standard Surveillance arm, QoL could either be influenced by the assurance of being closely monitored by being in a study or by the uncertainty of the possibility of being in the Intensive Surveillance arm and possibly have a positive finding in the biomarkers.
In the Intensive Surveillance arm – in case of unblinding due to positive findings – patients could show an increase in QoL by the reassurance that they are free of disease.
Furthermore, QoL may be increased hypothetically by detecting disease before development of symptoms, by virtue of early intervention, by delaying the time to the onset of symptoms, by prolonging survival and potentially even cure patients. QoL might improve by the intervention alone, but also in case of an increased OS. Moreover, earlier detection of distant recurrence and (oligo-)metastatic disease might reduce the need for aggressive cytotoxic chemotherapy regiments and may provide a chance of full recovery in the future, even at the stage of secondary disease.
However, a positive finding in biomarkers and consecutive staging examinations, which - in case of a molecular lead time - show no evidence of disease, could lead to an increase in fear of progression and therefore a deterioration of QoL.
Thus, QoL is to be evaluated in both groups with two different questionnaires: EORTC QLQ-C30 asking about general wellbeing in breast cancer patients and PA-F12 focusing on the fear of disease progression.
The questionnaires will be completed every six months during the interventional phase as well as every year during the follow-up phase.
An interim analysis will be performed one year after 50% of patients (n = 1750) have been enrolled in to the study.

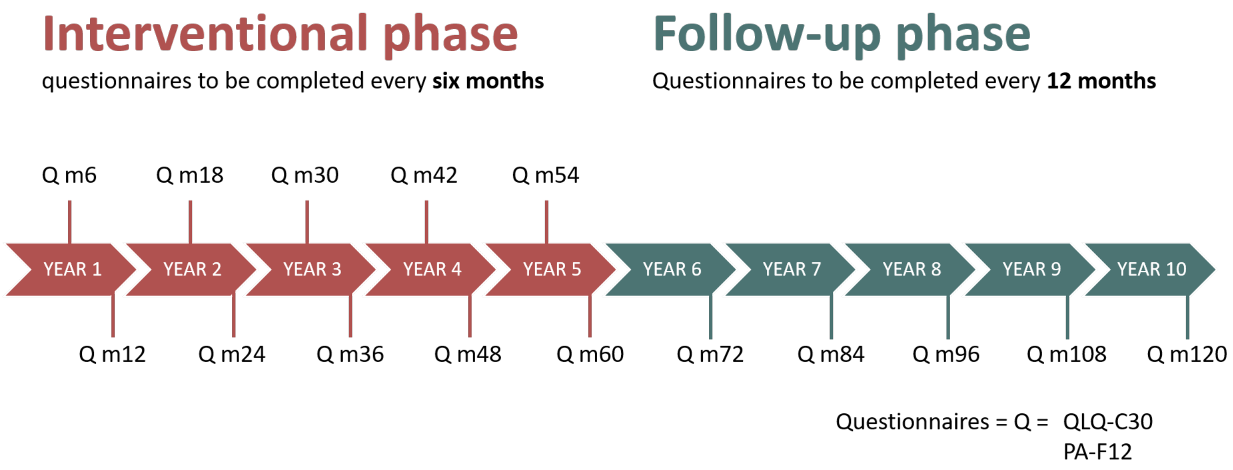

Figure 14: Timeline for QoL-Assessment

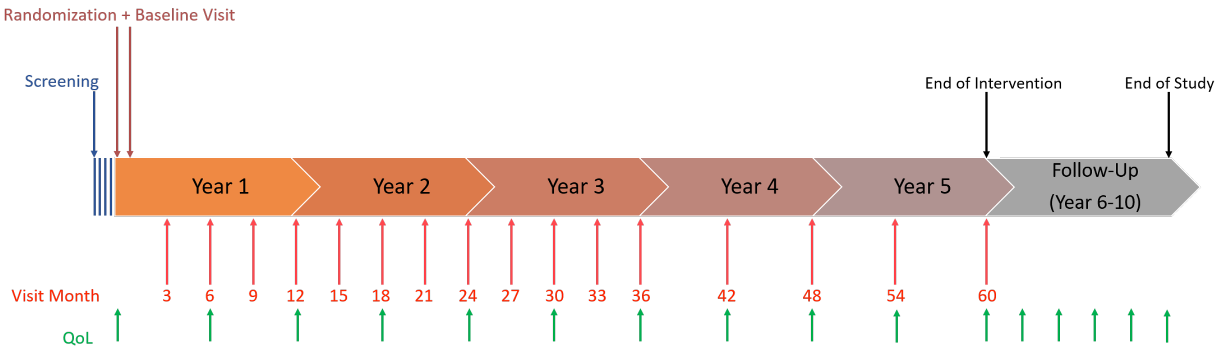

Figure 15: Timeline for QoL-Assessment respective to study visits

18.2 Questionnaires

QoL will be assessed every six months over the five years of interventional phase and every year in the five year follow-up phase in both arms. The validated and well-evaluated questionnaires EORTC QLQ-C30 and PA-F12 are used to analyze QoL with a focus on fear of progression as well as to evaluate any deterioration due to additional measures in the Intensive Surveillance arm at an early stage. The main issues affecting QoL in breast cancer follow-up will be fear (of progression/recurrence) and depression. The EORTC QLQ-C30 is a standardized and widely used questionnaire specifically for cancer patients with very good comparability, used since 1993. It consists of 30 questions to evaluate QoL in a multidimensional way with 10 different sub scales (60). The fear of progression will also be evaluated in the PA-F12 (German for FoP-Q-SF), which is a validated and reliable instrument and has been widely studied in breast cancer patients as well. In this short form of the PA-F, 12 items are inquired which form five sub scales. The general value for fear of progression can be seen as a sum of all items with a higher value corresponding to a higher level of fear of progression (61).
The questionnaires will be supplied to patients on paper and should be answered at the study center appointment. After submission, data will be entered in the eCRF.

18.3 Digital Health Application

The Digital Health Application may be used for Follow-Up questionnaires and may be used for study-related appointment planning.
If the participants decide to use the Digital Health Application, it is required to install the specific application on the participant's smart phone and consent to the data processing through the application. An individual username and password will be generated for each patient.
With the Digital Health Application, the participant will get a reminder on the due date of the Follow-Up questionnaire and the questionnaires can be answered digitally. If the questionnaires are not answered, the participant will get another friendly reminder. If the participant does not want to continue to use the application, it can be deleted any time from the smart phone in use. The study center should be informed by the patient if paper-based questionnaires are needed.

18.4 Analysis

QoL data will be analyzed according to the instructions of the validated questionnaires used.

19 DATA HANDLING AND DATA QUALITY ASSURANCE

19.1 Data Management and Documentation

Data management, which comprises CRF design, database creation, data processing and data validation will be performed by the IFG GmbH Erlangen for all study sites.
The IFG GmbH Erlangen will provide the investigator site with a web-based EDC system that is fully validated and conforms to 21 CFR Part 11 requirements. Investigator site staff will not be given access until they have been trained on the EDC system.

19.1.1 Data Entry and Queries

All CRF data will be entered into the study database by trained members of the investigator site using the web-based EDC system, which will perform automated plausibility and value range checks before accepting the data into the database.
Data that doesn't match the study guidelines will be queried and followed up on with the sites until satisfactory resolution.

19.1.2 Data Validation

Visual and computerized methods of data validation are applied in order to ensure accurate, consistent and reliable data for the subsequent analyses.

19.1.3 Registration / Recruitment Stop

At the end of recruitment, new patient registration and randomization functionality via EDC system is stopped and locked, although documentation for registered patients is possible until database lock.

19.1.4 Database Lock

Database lock for the primary analysis will be initiated at EOS, after all data has been entered and queries have been handled. After database lock no data can be changed. The database is locked to any kind of manipulation and then handed over to the Statistics Department. Further database locks for exploratory analyses or quality control measures will be done when necessary.

19.2 Privacy Protection and Data Safety

19.2.1 Pseudonymization

In order to protect patient data confidentiality and for safeguarding the privileged doctor patient relationship, each participating patient is assigned a unique patient number. Instead of the true patient identity the pseudonym is used in all communication.

19.2.2 User Access Control

Every user is provided with a unique personal username and unique password which defines their access to the web-based EDC system. Access control is based on the users' role in the web-based EDC system. Therefore, users can only access and amend those datasets necessary for them to fulfil their tasks.

19.3 Record retention after study completion

After completion of the study, all documents and data relating to the study will be kept in an orderly manner by the investigator in a secure study file. This file will be available for inspection by the sponsor or their representatives. Essential documents must be retained for twenty-five (25) years after completion of the study (here: End of Follow-Up).

20 STATISTICS

The statistical analysis of the present study is performed in accordance with the principles stated in the Consensus Guideline E9 (Statistical Principles for Clinical Trials) of the International Conference on Harmonization (ICH) including E9 (R1) addendum.

20.1 Randomization and stratification

Patients will be randomized centrally using permuted block list only if they fulfil the inclusion criteria and are not precluded from participation by any of the exclusion criteria.
Randomization will be stratified using block randomization by:

- Hormone receptor status (negative vs positive)
- HER2 status (negative vs positive)
- Histological lymph node status at surgery ((y)pN0 vs (y)pN+)

20.2 Analysis Sets

The following study population sets will be examined.
If necessary, further data sets required for additional analyses may be specified in separate analysis plans.

20.2.1 Intent-to-Treat (ITT) Set

The ITT set includes all patients who are randomized with signed informed consent. Patients will be analyzed according to the group they were randomized to.

20.2.2 Per Protocol (PP) Set

The PP set includes all patients of the ITT set who did not violate any inclusion or exclusion criterion and who were treated strictly according to protocol.

20.3 Sample Size Determination

The study is designed as a two-arm parallel, (partially) double-blinded randomized superiority study. To detect a 2.35% improvement in 5-year OS rate from 92.65% in the standard surveillance arm to 95.0% in the tumor marker, ctDNA and CTC guided surveillance arm (equivalent to a hazard ratio of 0.672), approximately 3500 randomized patients (1750 in both arms) will be required to achieve 80% power at a 2-sided significance level of 5%, assuming a uniform accrual pattern (accrual time 2 years), a dropout rate of 15% in both arms and a total study duration of 10 years. The reference value of 92.65% 5-year OS rate is based on the recently published results from the randomized phase III Success A study (Clinicaltrials.gov NCT02181101) that reported 5-year OS rates for 3754 intermediate-to-high-risk early breast cancer patients receiving standard chemotherapy regimen.

20.4 Patient disposition, data re-coding and display

Patients' demographics, baseline characteristics, protocol compliance and study withdrawals will be summarized by randomization arm in the intent-to-treat population. For categorical variables, frequency tables with absolute and relative frequencies will be presented with descriptive listings of details specified in text fields, when appropriate. Continuous variables will be reported using mean and standard deviation and/or median, range and interquartile range when appropriate. A CONSORT diagram will be used to document the flow of patients through the various stages of the study. The number of patients included in the various analysis populations will be presented in a table and reasons for exclusions will be detailed in listings.

20.5 Analysis of Primary Endpoint(s)

The primary endpoint overall survival will be analyzed based on the ITT set, estimated by the Kaplan Meier product limit method, and 5-year OS rates, 95% confidence intervals and survival plots will be provided. Overall survival will be compared between patients in the two randomization arms using the log-rank test, and univariable cox regression as well as additional multivariable analyses adjusted for other factors will be performed using suitable regression models (cox proportional hazard regression model). Hazard ratios and the corresponding 95% confidence intervals will be reported as obtained by both univariable and adjusted multivariable analyses.
The co-primary endpoint Overall Lead Time Effect will be calculated as described in chapter 9.1. As it is a purely descriptive composite measure consisting of two unrelated median values calculated for different patient cohorts, no confidence intervals will be provided.

20.6 Analysis of Secondary Endpoints

All analyses regarding the secondary objectives will have exploratory character only. All secondary endpoints and other outcomes that are calculated based on frequencies/rates will be summarized using frequency tables presenting absolute and relative frequencies together with appropriate confidence intervals. Comparisons between groups will be performed using suitable non-parametric statistical tests such as Fisher's exact test, $\chi 2$-test, or Cochran-Mantel-Haenszel test. Continuous variables will be summarized by descriptive statistics (number of valid and missing observations, mean, standard deviation, median, interquartiles, minimum, and maximum) and compared between arms using suitable non-parametric tests such as Wilcoxon-Mann-Whitney. Secondary survival endpoints (IDFS, DDFS, DRFS, BCSS) will be analyzed in the same way as the primary endpoint OS. Tumor marker (CA27.29, CEA, CA125) levels, presence and number of CTCs as well as presence of ctDNA measured at different time points will be evaluated in a descriptive way, and the temporal changes will be described and analyzed using appropriate statistical models (e.g., generalized linear mixed models). Quality of life data will be analyzed according to the instructions of the validated questionnaires used (EORTC QLQ-C30 and PA-F12).
More details regarding the statistical analyses will be provided in a Statistical Analysis Plan (SAP) which will be finalized prior to data base lock.

20.7 Pre-planned subgroup analyses

Subgroup analyses will be conducted to assess the consistency of the results (primary and secondary endpoints) across the following prespecified key subgroups: Hormone receptor status (negative vs positive), HER2 status (negative vs positive), histological lymph node status at surgery ((y)pN0 vs (y)pN+). These subgroup analyses will be exploratory. The presence of treatment heterogeneity regarding the primary endpoint OS will be examined by testing the presence of an interaction in a Cox model with the key variable of interest, treatment indicator and the interaction term, stratifying for the other stratification factors. The hazard ratio and associated confidence interval for the treatment effect will be calculated within the subgroups of interest from the model. The same level of confidence as for the overall population will be used for the confidence intervals in the subgroups. Furthermore, the estimated hazard ratios in all subgroups and the p-value for interaction will be displayed on a forest plot. Furthermore, possible heterogeneity of the results between patients below and above 70 years old will be similarly investigated. Additional exploratory subgroup analyses will be performed as appropriate.

The results of these subgroup analyses should be interpreted with caution. No type I error adjustment will be made for multiple testing.

20.8 Interim/Futility Analyses

20.8.1 Interim analyses for efficacy

No formal efficacy interim analysis is planned.

20.8.2 Futility analyses

No formal efficacy interim analysis is planned.

A three-step futility analysis for ctDNA is planned (as per request of the contributing company):

<u>Step 1:</u>

Analysis of minimum requirement regarding ctDNA positivity rate.

- Analysis is performed based on the first 150 patients enrolled in the experimental arm to calculate the ctDNA positivity rate.
- Analysis is considered to be affirmative to continue ctDNA testing within the study,
    - if the ctDNA positivity rate measured 3 months after recruitment is > 5% for these 150 patients and
    - if the ctDNA positivity rate measured 12 months after recruitment is > 10% for these 150 patients.

Step 2:

Analysis of minimum requirement regarding molecular to via imaging verified distant recurrence lead time (from the time of ctDNA positive result – i.e., molecular relapse - to the time of image-verified distant relapse; see detailed definition in "Secondary Objectives and Endpoints")

- Analysis is performed after 30 patients in the experimental arm had a ctDNA positive result and a via imaging verified distant recurrence (without additional treatment intervention) and a minimum follow-up time of 6 months (as from the first ctDNA-positive result), to calculate the median lead time from the time of the first ctDNA positive result to the time of via imaging verified distant relapse for these 30 patients.
- Analysis is considered to be affirmative to continue ctDNA testing within the study, if this lead time is > 6 months.

Step 3:

Analysis of minimum requirement regarding overall lead time (molecular relapse to via imaging verified distant recurrence lead time + Difference in median time to distant recurrence between the study arms; see detailed definition in "Primary Objectives and Endpoints" above)

- Analysis is performed after 50 patients in the experimental arm had a ctDNA positive result and a via imaging verified distant recurrence and a minimum follow-up time of 12 months (as from the first ctDNA positive result), to calculate the overall lead time (molecular relapse to via imaging verified distant recurrence lead time + Difference in median time to distant recurrence between the study arms) for these 50 patients.
- Analysis is considered to be affirmative to continue ctDNA testing within the study, if this overall lead time is > 9 months.

If these requirements for continuation of ctDNA testing are not fulfilled, termination of ctDNA testing in this trial might be considered pending decision of the steering board and the company

20.8.3 Interim analysis for Quality of Life (QoL)

An informal, nonbinding interim analyses of QoL will be performed one year after 50% of patients (n = 1750) have been enrolled. Based on the individual data from the PA-F12 and EORTC QLQ C30 questionnaires obtained from these patients 12 months after enrollment, differences in QoL between the two randomization arms will be evaluated (with the main focus being fear of progression and depression) to ensure that patients in the Intensive Surveillance arm do not suffer from unacceptable emotional burden as compared to the standard arm. The findings will be presented to the IDMC to decide if (and if yes, which) measures should be taken to lessen the emotional burden for patients in the Intensive Surveillance arm. This analysis will be repeated at regular intervals according to DSMB requirements to ensure that there is no long-term impairment on patients' emotional well-being because of the study. Regular study monitoring regarding compliance of blood sampling and evaluation of the reasons for incompliance or even drop-outs will complement the QoL assessments.

21 INDEPENDENT DATA MONITORING COMMITTEE (IDMC)

21.1 IDMC Members and Mission

In addition to the Protocol Board and Steering Committee, the IDMC and the IFG GmbH Erlangen review and observe the conduct of the study.

The members are independent of the study and familiar with the methodology of oncology trials. They are aware of the risks of conclusions based on immature data and have agreed with the design and the goals of this protocol. IDMC meetings are held regularly; if necessary (for example, if results of an interim safety analysis become available between regular meetings), ad hoc IDMC meetings may be organized. The mission of the IDMC is to ensure the ethical conduct of the study and to protect patients' safety interests in this study.

21.2 Documentation Provided to the IDMC

Before any meeting of the IDMC, the statistician should provide the IDMC with at least the following key documents:

- Study protocol
- Recruitment summary
- Patient baseline characteristics
- Narratives of deaths
- (Distant) disease recurrence rates
- Summary of follow-up

All data will be broken down by treatment arm, participating institution and patient (whenever necessary).

21.3 Recommendations of the IDMC

After each meeting, the IDMC will provide the Protocol Board/Steering Committee with a written recommendation to either modify the study (with reasons), or discontinue the study (with reasons), or continue the study unchanged. The final decision to amend the protocol or to discontinue the study will be taken only by the Protocol Board.

Early termination of the study will be considered by the Protocol Board/Steering Committee based on the suggestion of the IDMC if less than 500 patients are recruited within 12 months.

For all the participants of the Protocol Board and Steering Committee, see sections 2 and 3.

22 ADMINISTRATIVE EXECUTION

22.1 Monitoring and Auditing

All source data verification (SDV) and source data review (SDR) is conducted according to ICH-GCP guidelines, the and the Trial Monitoring Plan (TMP). The investigator must permit study monitors, the sponsor's auditors to inspect all study-related documents and pertinent hospital or medical records for confirmation of data contained within the CRFs.
Source documentation will be reviewed and verified in a risk-based approach for adequacy and consistency.

22.2 Sponsor's Responsibilities

The University Hospital Ulm as the sponsor

- agrees to provide the investigator with sufficient material and support to permit the investigator to conduct the study according to the agreed protocol.
- reserves the right to request the withdrawal of a patient due to protocol deviations, administrative or other reasons.
- reserves the right to terminate the study prematurely due to persistent protocol deviations of the site, administrative or other reasons. Should this be necessary, the procedures will be arranged after review and after consultation by both parties to ensure protection of the patients' interests.

22.3 Investigator's Responsibilities

The investigator agrees to conduct the study in accordance with and the procedures and requirements laid out in this protocol and by ICH-GCP guidelines. In particular, the investigator agrees to conduct the study in accordance with strict ethical principles. Any modification to the agreed protocol must be approved in writing by both University Hospital Ulm as the sponsor and, if appropriate, the ethics committee(s) approving the original protocol before any modifications are put into effect.
It is the responsibility of the investigator to complete the CRFs for each patient in the study, and when a patient completes the study, the investigator must (electronically) sign all CRFs.
In addition to the CRFs, the investigator will maintain adequate records that fully document the progress of the study. The investigator has to state that the patient has taken part in a study and record the study number in the patient's medical records. The exact dates of the beginning and the end of intervention should be given as well.
Copies of these study records (and all study-related documents) shall be kept by the investigator for 25 years by the hospital, institution or private practice. All documentation and materials provided by IFG GmbH Erlangen and the University Hospital Ulm for this study are to be retained in a secure place and treated as confidential material.

The investigator has the right to request termination of the study for administrative or other reasons. Should this be necessary and agreed upon, the procedures will be arranged after review and after consultation by both parties, to ensure protection of the patients' interests.
By signing this document, the investigator indicates that he/she has read the protocol, fully understands the requirements and agrees to abide by all protocol requirements.
Further obligations of the investigator are agreed on in the investigator's contract with the University Hospital Ulm as the sponsor.
The investigator will be responsible for retaining sufficient information about each patient (e.g., informed consent form, name, address, phone number, and identity in the study) so that the auditor of the University Hospital Ulm as the sponsor may access this information should the need to do so arise. These records should be retained in a confidential manner for as long as legally mandated according to local requirements.

22.4 Patient Informed Consent

Prior to the beginning of specific protocol procedures, the patient is informed about the nature of the study intervention and is given pertinent information as to the intended purpose, possible benefits, and possible adverse experiences. The procedures and possible hazards to which the patient will be exposed are explained. Patient insurance for the compensation of patients for possible study-related injury as well as a commuting accidents insurance policy is provided by the University Hospital Ulm as the sponsor according to local law.
An approved informed consent (ICF) statement will then be read and signed by the patient, and, if required, a witness, as well as the investigator. The patient will be provided with a copy of the signed ICF. The patient may withdraw from the study at any time in any way without prejudicing future medical treatment.
Patients are informed that pseudonymized data from their case may be stored electronically and that such data will not be revealed to any unauthorized third party. Data will be reviewed by the monitor, an independent auditor and possibly by representatives of ethics committees. The terms of the local data protection legislation will be applied as appropriate.

22.5 Ethics and Regulatory Considerations

This study will be conducted according to the ICH-GCP guideline in compliance with the recommendation of the German task force of medical ethical committees regarding studies that are neither part of the German drug law nor part of the German law on medical products (Arbeitskreis Medizinischer Ethikkommissionen; „Empfehlung für den Umgang mit multizentrischen Studien außerhalb von AMG oder MPG durch Ethik-Kommissionen“) and applicable regulations in all aspects of preparation, monitoring, reporting, auditing, and archiving along the § 15 Musterberufsordnung (MBO). The final approved protocol and the ICF is reviewed by a properly constituted Ethics Committee. The decision of Ethics Committee concerning the conduct of the study is made in writing to the investigator.

22.6 Declaration of Helsinki

This study is to be performed in accordance with the Declaration of Helsinki (62).

22.7 Study Conduct

This study will be conducted according to the ICH-GCP in compliance with the recommendation of the German task force of medical ethical committees regarding studies that are neither part of the German drug law nor part of the German law on medical products (Arbeitskreis Medizinischer Ethikkommissionen; „Empfehlung für den Umgang mit multizentrischen Studien außerhalb von AMG oder MPG durch Ethik-Kommissionen“) and their regulatory requirements.

22.8 Modification of the Protocol

Any modifications to the protocol which may impact on the conduct of the study, on the potential benefit of the patient or may affect patient safety, including changes of study objectives, study design, patient population, sample sizes, study procedures, or significant administrative aspects, will require a formal amendment to the protocol. Such an amendment will be agreed upon by the Protocol Board, and approved by the Ethics Committee prior to implementation.
Administrative changes of the protocol are minor corrections and/or clarifications that have no effect on the way the study is to be conducted. These administrative changes will be agreed upon by the Protocol Board and will be documented in a memorandum. The Ethics Committee may be notified of administrative changes at the discretion of the investigator.

22.9 Study Documents

All information concerning the intervention and study conduction, such as scientific data and material not previously published are considered confidential and shall remain the sole property of University Hospital Ulm.
The investigator agrees to use the information provided for the conduct of this study only and to use it for no other purposes unless she/he obtains the written consent of the University Hospital Ulm as the sponsor.

22.10 Case Report Forms

All key study information must be recorded in the patient’s hospital chart.
Study procedures will be fully online documented on the electronic CRFs (eCRF) provided through the web-based EDC system. The data from the questionnaires of the study (study visit questionnaires, QoL questionnaires) will also be fully documented and the data collected via Digital Health Application will be transmitted to the IFG GmbH Erlangen. Both, data from questionnaires via Digital Health Application and on-paper questionnaires will be transferred into the eCRF.

Relevant laboratory reports with study number, patient number and medical interpretation of results have to be signed and dated by the investigator.
The CRFs remain in the property of the University Hospital Ulm and the IFG GmbH Erlangen at all times. On the CRFs, patients should be identified only by their patient number.
Patients' data entries may only be made by the persons registered on the form "Delegation of responsibilities and signature list of investigators and medical staff".
For details concerning the CRF submission process, please refer to the application manual and training material.

22.11 Necessary Documents

The following documents are collected from the investigator's site:

- Signed Investigator's Agreement
- Scientific Curriculum vitae of Principal Investigator
- Form "Delegation of responsibilities and signature list of investigators and medical staff"
- Responsibility of data handling and protection according to DSGVO

22.12 Archiving

Any records and documents relating to the conduct of this study, including ICFs, eCRFs, QoL data, laboratory test results, and imaging records, must be retained by the Principal Investigator for a minimum of twenty-five (25) years after completion (here: End of Follow-Up) or discontinuation of the study, or for the length of time required by relevant national or local health authorities, whichever is longer. After that period of time, the documents may be destroyed, subject to local regulations. Notification should be provided to the Sponsor prior to transferring any records to another party or moving them to another location.

22.13 Use of Information and Publication

To allow for the use of the information derived from this clinical study and to ensure compliance to current regulations, the investigator is obliged to provide the sponsor with complete test results and all data obtained in this study. The final statistical study report will be prepared by the responsible biostatistician and the final medical report by the coordinating investigator and the sponsor.
The final study report will be a publication in a peer-reviewed journal under the responsibility of the Sponsor according to publication regulations.
The Protocol Board will review the manuscript to prevent forfeiture of patent rights to data not in the public domain. The authorship list will be agreed upon by University Hospital Ulm and the investigators prior to publication.

22.14 Finance and Insurance

Details on finance and insurance will be outlined in a separate agreement between the investigator and the sponsor.

23 INSURANCE COVER

The sponsor takes out an insurance policy for all study participants covering all injuries patients suffer from due to the study participation. Furthermore, the sponsor takes out a commuting accidents insurance policy for all study participants covering all injuries patients suffer on their direct commute to and from study centers. A certificate of insurance will be provided to the investigator.
Compensation is limited to 500.000,00 Euro at most per patient and 5.000.000,00 Euro at most for the whole study. Additional limits for travel insurance cover 100.000,00 Euro at most per person for invalidity or death.

Insurance provider:	Chubb European Group SE, Direktion für Deutschland, Baseler Straße 10, 60329 Frankfurt am Main, Germany
Police-No.:	DELSCA45932
Policy Holder:	Universitätsklinikum Ulm, Albert-Einstein-Allee 29, 89081 Ulm, Germany

The investigator informs each patient on his responsibilities resulting from the terms of insurance.

24 GLOSSARY OF ABBREVIATIONS AND ACRONYMS

°C	degree Celsius
µg	microgram
AGO	Arbeitsgemeinschaft für Gynäkologische Onkologie (German study group for
APC	Allophycocyanin (APC)
ASCO	American Society of Clinical Oncology
BCSS	Breast cancer specific survival
CA	Cancer antigen
CD	Cluster of differentiation
CDK	cyclin-dependent kinase
CEA	Carcinoembryonic antigen
CFR	Code of Federal Regulations
CI	confidence interval
CK-PE	cytokeratin-phycoerythrin
CRF	Case Report Form
CT	computed tomography
CTC	Circulating tumor cell
ctDNA	Circulating tumor deoxyribonucleic acid
DALYs	disability-adjusted life-years
DAPI	4′,6-diamidino-2-phenylindole
DCIS	ductal carcinoma in situ
DDFS	distant disease-free survival
DFS	disease-free survival
DNA	deoxyribonucleic acid
DRFS	distant recurrence-free survival
DSGVO	Datenschutzgrundverordnung der Europäischen Union
DSMB	Data Safety Monitoring Board
DSUR	Development Safety Update Report
e. g.	exempli gratia
e. V.	eingetragener Verein (registered organization)
ECLIA	ElectroChemiLuminescence-ImmunoAssay
ECOG	Eastern Cooperative Oncology Group
eCRF	electronic Case Report Form
EDC	electronic data capture
EDTA	Ethylenediaminetetraacetic acid
EpCAM	epithelial cellular adhesion molecule
EOI	End of Intervention
EORTC	European Organisation for Research and Treatment of Cancer
EORTC QLQ-C30	EORTC Core Quality of Life questionnaire with 30 question
EOS	end of study
EOT	end of treatment
ER	estrogen receptor
ET	endocrine therapy
Etc.	etcetera

EudraCT	European Union Drug Regulating Authorities Clinical Trials
FDA	Food and Drug Administration
FFPE	Formalin-fixed, paraffin-embedded
FIGO	The International Federation of Gynecology and Obstetrics
FoP-Q-SF	Fear of Progression Questionnaire-Short Form
FPI	first patient in
FU	Follow up
GCP	Good Clinical Practice
GmbH	Gesellschaft mit beschränkter Haftung (Limited Company)
GnRH	Gonadotropin-releasing Hormone
H	hour
H&E	Hematoxylin and eosin
HER	human epidermal growth factor receptor
HR	hormone receptor
HTTP	Hypertext Transfer Protocol
i. a.	inter alia
i. e.	id est
IBCFS	invasive breast cancer free survival
IB	Investigator's Brochure
ICF	informed consent form
ICH	International Conference on Harmonization of Technical Requirements for Registration of Pharmaceuticals for Human Use
iDFS	invasive disease-free survival
IDMC	Independent Data Monitoring Committee
IFG GmbH Erlangen	Institut für Frauengesundheit GmbH Erlangen
LP	last patient
LPI	last patient in
mL	milliliter
MRD	molecular residual disease
MRI	magnetic resonance imaging
MUC	mucin
mTOR	mechanistic Target of Rapamycin
NCI	National Cancer Institute
ng	nanogram
NGS	Next Generation Sequencing
Ob/Gyn	Obstetrics and Gynecology
OS	overall survival
PA-F12	Progredienzangstfragebogen mit 12 Fragen (fear of progression questionnaire with 12 questions)
PARP	Poly ADP (Adenosine Diphosphate)-Ribose Polymerase
PCR	polymerase chain reaction
PET	positron emission tomography
PgR	progesterone receptor
PI	principle investigator
PI3K	phosphatidylinositol 3-kinase

PP	Per Protocol
PRO	Patient reported outcome
QoL	Quality of life
RaDaR™	Residual Disease and Recurrence
SAP	Statistical analysis plan
SDR	source data review
SDV	source data verification
SERMs	Selective estrogen receptor modulators
SNP	Single-nucleotide polymorphism
SNV	single nucleotide variants
SOP	standard operations procedures
SPECT	single-photon emission computerized tomography
TDM-1	Trastuzumab emtansine
T-Dx	Trastuzumab deruxtecan
TM	Tumor marker
TMP	Trial Monitoring Plan
TNBC	triple-negative breast cancer
U	Unit
USA	United States of America
vs.	versus
WES	whole exome sequencing
WMA	World Medical Association
YLD	years lived with disability
YLL	years of life lost

25 INVESTIGATOR SIGNATURE PAGE

SURVIVE (Standard Surveillance vs. Intensive Surveillance in Early Breast Cancer) – a partially double-blinded, multi-center, randomized, controlled superiority study

**Protocol Version:**	**2.0**
**Protocol Version Date:**	**02. March 2023**
**BMBF Funding Code:**	**01KD2202**
**ClinicalTrials.gov-ID:**	**NCT05658172**
**DRKS-ID:**	**DRKS00030745**

I confirm that I have read the above-mentioned protocol and its attachments and agree that it contains all necessary details for carrying out the study as described. I agree to conduct this study in accordance to the procedures specified in the ICH-GCP in compliance with the recommendation of the German task force of medical ethical committees regarding studies that are neither part of the German drug law nor part of the German law on medical products (Arbeitskreis Medizinischer Ethikkommissionen; „Empfehlung für den Umgang mit multizentrischen Studien außerhalb von AMG oder MPG durch Ethik-Kommissionen“) as well as to the regulations of the basic regulatory regarding data security of the European Union (DSGVO – Datenschutzgrundverordnung). I will make a reasonable effort to complete the study within the time designated. I will ensure that all associates, colleagues and employees assisting in the conduct of the study are informed of their obligations and will be kept informed throughout the study: I confirm that I and study personnel participating under my supervision have adequate resources to fulfill their responsibilities as outlined in this protocol. I will maintain documentation of any investigator responsibilities assigned to participating study personnel. I confirm that all data will be submitted in a timely manner and will be accurate, complete and supported by source documents. I will provide copies of the protocol and access to all further available information on the study to study personnel under my supervision. I will discuss this material with them to ensure that they are fully informed about the study.

The contents may not be used in any other clinical study and may not be disclosed to any person except study personnel who will be committed to secrecy. Any supplemental information that may be added to this document is also confidential and must be kept in confidence in the same manner as the contents of this protocol.

I understand that the protocol may not be modified without written approval by the sponsor.

I am informed that I may terminate or suspend enrollment of the study at any time if it becomes necessary to protect the best interests of the study subjects. However, I will give prompt notice to the coordinating investigator. The sponsor and the coordinating investigator may terminate the study at any time with or without stating a cause.

I understand that this study has to obtain a favorable opinion from the competent local ethics committee. Before these have been obtained no patient will be included in the study.

Principal Investigator’s Name **(CAPITAL LETTERS)**

Site Address, Phone Number and E-Mail

Principal Investigator’s Signature

Date

26 APPENDICES

26.1 Appendix A: Quality of Life Questionnaires

26.1.1 EORTC-QLQ C30

EORTC QLQ-C30 (Version 3)

Wir sind an einigen Angaben interessiert, die Sie und Ihre Gesundheit betreffen. Bitte beantworten Sie die folgenden Fragen selbst, indem Sie die Zahl einkreisen, die am besten auf Sie zutrifft. Es gibt keine „richtigen“ oder „falschen“ Antworten. Ihre Angaben werden streng vertraulich behandelt.

Bitte tragen Sie Ihre Initialen ein: |_|_|_|_|
Ihr Geburtsdatum (Tag, Monat, Jahr): |_|_|_|_|_|_|_|_|
Das heutige Datum (Tag, Monat, Jahr): 31 |_|_|_|_|_|_|_|_|

		Überhaupt nicht	Wenig	Ziemlich	Sehr
1.	Bereitet es Ihnen Schwierigkeiten, sich körperlich anzustrengen (z. B. eine schwere Einkaufstasche oder einen Koffer zu tragen)?	1	2	3	4
2.	Bereitet es Ihnen Schwierigkeiten, einen längeren Spaziergang zu machen?	1	2	3	4
3.	Bereitet es Ihnen Schwierigkeiten, eine kurze Strecke außer Haus zu gehen?	1	2	3	4
4.	Müssen Sie tagsüber im Bett liegen oder in einem Sessel sitzen?	1	2	3	4
5.	Brauchen Sie Hilfe beim Essen, Anziehen, Waschen oder Benutzen der Toilette?	1	2	3	4

Während der letzten Woche:

		Überhaupt nicht	Wenig	Ziemlich	Sehr
6.	Waren Sie bei Ihrer Arbeit oder bei anderen tagtäglichen Beschäftigungen eingeschränkt?	1	2	3	4
7.	Waren Sie bei Ihren Hobbys oder anderen Freizeitbeschäftigungen eingeschränkt?	1	2	3	4
8.	Waren Sie kurzatmig?	1	2	3	4
9.	Hatten Sie Schmerzen?	1	2	3	4
10.	Mussten Sie sich ausruhen?	1	2	3	4
11.	Hatten Sie Schlafstörungen?	1	2	3	4
12.	Fühlten Sie sich schwach?	1	2	3	4
13.	Hatten Sie Appetitmangel?	1	2	3	4
14.	War Ihnen übel?	1	2	3	4
15.	Haben Sie erbrochen?	1	2	3	4
16.	Hatten Sie Verstopfung?	1	2	3	4

**Während der letzten Woche:**	**Überhaupt nicht**	**Wenig**	**Ziemlich**	**Sehr**
17. Hatten Sie Durchfall?	1	2	3	4
18. Waren Sie müde?	1	2	3	4
19. Fühlten Sie sich durch Schmerzen in Ihrem alltäglichen Leben beeinträchtigt?	1	2	3	4
20. Hatten Sie Schwierigkeiten, sich auf etwas zu konzentrieren, z. B. auf Zeitunglesen oder Fernsehen?	1	2	3	4
21. Fühlten Sie sich angespannt?	1	2	3	4
22. Haben Sie sich Sorgen gemacht?	1	2	3	4
23. Waren Sie reizbar?	1	2	3	4
24. Fühlten Sie sich niedergeschlagen?	1	2	3	4
25. Hatten Sie Schwierigkeiten, sich an Dinge zu erinnern?	1	2	3	4
26. Hat Ihr körperlicher Zustand oder Ihre medizinische Behandlung Ihr Familienleben beeinträchtigt?	1	2	3	4
27. Hat Ihr körperlicher Zustand oder Ihre medizinische Behandlung Ihr Zusammensein oder Ihre gemeinsamen Unternehmungen mit anderen Menschen beeinträchtigt?	1	2	3	4
28. Hat Ihr körperlicher Zustand oder Ihre medizinische Behandlung für Sie finanzielle Schwierigkeiten mit sich gebracht?	1	2	3	4

Bitte kreisen Sie bei den folgenden Fragen die Zahl zwischen 1 und 7 ein, die am besten auf Sie zutrifft:

29. Wie würden Sie insgesamt Ihren Gesundheitszustand während der letzten Woche einschätzen?

1	2	3	4	5	6	7
sehr schlecht						ausgezeichnet

30. Wie würden Sie insgesamt Ihre Lebensqualität während der letzten Woche einschätzen?

1	2	3	4	5	6	7
sehr schlecht						ausgezeichnet

26.1.2 PA-F12

PA-F

Im folgenden finden Sie eine Reihe von Aussagen, die sich alle auf Ihre Erkrankung und mögliche **Zukunftssorgen** beziehen. Bitte kreuzen Sie bei jeder Aussage an, was für Sie zutrifft. Sie können wählen zwischen "nie", "selten", "manchmal", "oft" und "sehr oft". Bitte lassen Sie keine Frage aus.

Sie werden sehen, daß einige Fragen nicht auf Sie zutreffen. Wenn Sie beispielsweise keine Familie haben können Sie Fragen zur Familie eigentlich nicht beantworten. Wir bitten Sie, in diesen Fällen ein Kreuz bei "nie" zu machen.

	**nie**	**selten**	**manch-mal**	**oft**	**sehr oft**
1. Wenn ich an den weiteren Verlauf meiner Erkrankung denke, bekomme ich Angst.	☐	☐	☐	☐	☐
2. Vor Arztterminen oder Kontrolluntersuchungen bin ich ganz nervös.	☐	☐	☐	☐	☐
3. Ich habe Angst vor Schmerzen.	☐	☐	☐	☐	☐
4. Der Gedanke, ich könnte im Beruf nicht mehr so leistungsfähig sein, macht mir angst.	☐	☐	☐	☐	☐
5. Wenn ich Angst habe, spüre ich das auch körperlich (z.B. Herzklopfen, Magenschmerzen, Verspannung).	☐	☐	☐	☐	☐
6. Die Frage, ob meine Kinder meine Krankheit auch bekommen könnten, beunruhigt mich.	☐	☐	☐	☐	☐
7. Es beunruhigt mich, daß ich im Alltag auf fremde Hilfe angewiesen sein könnte.	☐	☐	☐	☐	☐
8. Ich habe Sorge, daß ich meinen Hobbies wegen meiner Erkrankung irgendwann nicht mehr nachgehen kann.	☐	☐	☐	☐	☐
9. Ich habe Angst vor drastischen medizinischen Maßnahmen im Verlauf der Erkrankung.	☐	☐	☐	☐	☐
10. Ich mache mir Sorgen, daß meine Medikamente meinem Körper schaden könnten.	☐	☐	☐	☐	☐
11. Mich beunruhigt, was aus meiner Familie wird, wenn mir etwas passieren sollte.	☐	☐	☐	☐	☐
12. Der Gedanke, ich könnte wegen Krankheit in der Arbeit ausfallen, beunruhigt mich.	☐	☐	☐	☐	☐

26.2 Appendix B: Assessment of Performance status and activities of daily living

26.2.1 Determination of Performance Status

ECOG or Zubrod Scale		Karnofsky Score
0	Fully active; able to carry on all pre-disease performance without restriction	90–100%
1	Restricted in physically strenuous activity but ambulatory	70–80%
2	Ambulatory and capable of self-care, but unable to carry out any work activities	50–60%
3	Capable of only limited self-care; confined to bed or chair more than 50% of waking hours	30–40%
4	Completely disabled	10–20%

26.3 Appendix C: Deutsche Synopse

**Titel der Studie**	SURVIVE (Standard Surveillance vs. Intensive Surveillance in Early Breast Cancer) - Standard Nachsorge im Vergleich mit Intensivierter Nachsorge bei frühem Brustkrebs
**Sponsor**	Universitätsklinikum Ulm
**Phase**	Phase III
**Rationale**	Brustkrebs ist die häufigste Krebsart weltweit mit einem mittleren Erkrankungsalter von 64 Jahren bei Erstdiagnose. Allgemein sind jedoch alle Altersklassen ab der Pubertät betroffen. Das Auftreten von Brustkrebs ist in den letzten Jahrzehnten stetig angestiegen, jedoch sehen wir auch eine kontinuierliche Zunahme an neuen Behandlungsmethoden, die ein breites Spektrum an Therapiemöglichkeiten im Sinne von neuen Substanzen bieten.<br>Nach Abschluss der Primärtherapie ist die aktuelle Leitlinien-basierte Nachsorge bei BrustkrebspatientInnen auf die klinische Untersuchung und der Bildgebung der Brust (Mammographie) limitiert. Ein Screening auf Fernmetastasen wird nur initiiert, wenn ein Verdacht auf Metastasen durch klinische Symptome besteht. Grundlage für dieses Vorgehen sind zwei groß-angelegte Kohortenstudien, aus den 1980er Jahren, die keinen Vorteil eines regulären Screenings auf Fernmetastasen im Vergleich zur Standard-Nachsorge auf das Gesamtüberleben zeigen konnten.<br>In diesem Stadium der Nachsorge fühlen sich PatientInnen heute häufig allein gelassen. Ferner haben sich die Therapieoptionen für die Behandlung von früh diagnostiziertem, metastasiertem Brustkrebs in den letzten Jahren drastisch zum Guten verbessert, zum Beispiel mit der Einführung von Her2-gerichteter Therapie, der antihormonellen Kombinationstherapie mit CDK4/6- Inhibitoren, Antikörper-Drug-Konjugaten, Immuntherapien, PARP-Inhibitoren oder auch stereotaktischer Strahlentherapie – um nur eine Auswahl zu nennen.<br>Wir denken, dass in Hinblick auf die zur Verfügung stehenden, modernen therapeutischen Möglichkeiten eine frühere Erkennung von asymptomatischen Fernmetastasen (welche ggf. nur an einzelnen Lokalisationen vorliegen) und damit auch früherer Start einer zielgerichteten Therapie zu einer Verbesserung des Gesamtüberlebens führen kann.<br>In dieser Studie wollen wir hierzu Biomarker im Blut bestimmen (Liquid biopsy), welche als Hinweis zur Erkennung von asymptomatischen Fernmetastasen dienen könnten. Aufgrund vorausgehender Studien werden wir hierfür die konventionellen Tumormarker CA27.29 (entspricht CA 15.3), CA 125 und CEA testen sowie die Proben auf zirkulierende Tumorzellen (CTCs) und zirkulierende Tumor-DNA (ctDNA) testen.<br>Die Studie wird als multizentrische, prospektive, teilweise doppelt-verblindete, kontrollierte, randomisierte Studie bei Brustkrebspatientinnen mit mittlerem

	bis hohem Rezidivrisiko durchgeführt und könnte zu einem Paradigmenwechsel in der aktuellen Nachsorge führen.
**Überblick über die Studie**	Diese Studie wird als teilweise doppel-blinde, multizentrische, randomisierte und kontrollierte Überlegenheitsstudie durchgeführt, um den potenziellen Vorteil einer intensivierten Nachsorge gegenüber der Standard-Nachsorge bei Brustkrebspatientinnen mit mittlerem bis hohem Rezidivrisiko zu untersuchen. Nach Ende der primären Krebstherapie (adjuvante Chemotherapie, Operation oder Bestrahlung, je nachdem was zuletzt stattfand) sollen insgesamt 3500 Patientinnen in diese Studie 1:1 in einen der folgenden Arme randomisiert werden:<br><br>- **Standard Nachsorge** analog der nationalen Leitlinien oder<br>- **Intensivierte Nachsorge** mit zusätzlicher Testung von Blutproben auf Tumormarker (CA27.29, CA125, CEA), CTC und ctDNA<br><br>In beiden Studienarmen werden PatientInnen die Standard-Nachsorge analog der nationalen Leitlinien über Ihren behandelnden Nachsorge-Arzt erhalten, welche Nachsorgetermine alle 3 Monate in den ersten drei Jahren sowie alle 6 Monate in den folgenden zwei Jahren umfasst. In den Studienvisiten werden in beiden Studienarmen zusätzlich Blutproben entnommen und die Lebensqualität der Probandinnen analysiert.<br>In dem **Standard Nachsorge** Arm werden die Blutproben in einer Biobank gelagert und nicht direkt analysiert.<br>In dem **Intensivierten Nachsorge** Arm werden die Blutproben auf Tumormarker (CA27.29, CA125, CEA), CTCs und ctDNA getestet. Sollten sich auffällige Befunde in einem oder mehrerer der Biomarker zeigen (CA27.29 oder CA125 oder CEA oder CTC oder ctDNA), wird eine diagnostische Bildgebung zur Metastasensuche veranlasst. Zusätzlich werden in diesem Arm Blutproben, welche nicht direkt analysiert werden, in einer Biobank gesammelt und gelagert.<br>In beiden Armen wird die durch Bildgebung bestätigte Diagnose eines Rezidivs die Nachsorge beenden und die leitlinien-gerechte Therapie wird initiiert.<br>Der geplante Rekrutierungszeitraum umfasst ca. 24 Monate, die Gesamt-Studiendauer kalkulieren wir mit ca. 144 Monaten (bestehend aus einer zweijährigen Rekrutierungsdauer, einer fünfjährigen Interventionsphase und einer fünfjährigen Nachbeobachtungsphase).<br>Zur Langzeit-Nachbeobachtung werden die PatientInnen dazu angehalten, eine Patientenselbstauskunft zu nutzen.
**Intervention**	Allen StudienteilnehmerInnen ohne klinischen Nachweis eines Rezidives (Lokalrezidiv oder Fernmetastasen) wird nach Beendigung ihrer primären Brustkrebstherapie (adjuvante Chemotherapie, Operation oder Bestrahlung, je nachdem was als letztes durchgeführt wurde) zusätzlich zur regulären Nachsorge insgesamt 27,5 mL Blut abgenommen (2 x 10 mL Streck Cell-Free

	DNA BCT® Röhrchen, 1 x 7,5 mL Serum Monovette; alle 3 Monate für die ersten drei Jahre nach Einschluss, danach alle 6 Monate für zwei weitere Jahre). Sollten Fernmetastasen oder ein Lokalrezidiv festgestellt werden, sollen die leitlinien-gerechten diagnostischen und therapeutischen Maßnahmen eingeleitet werden.<br>Die Lebensqualität wird alle 6 Monate mit zwei anerkannten Fragebögen (EORTC QLQ C30 und PA-F12) abgefragt.<br>Alle StudienteilnehmerInnen werden bei den Studienvisiten über die stattgefundenen Untersuchungen (Blutabnahmen oder Bildgebung) außerhalb der Studie sowie Symptome abgefragt, welche auf Metastasen hindeuten könnten.<br>StudienteilnehmerInnen werden 1:1 in den Intensivierten oder Standard-Nachsorge Arm randomisiert. Die Zuteilung zu den Armen ist für die PatientInnen und das Zentrum verblindet, solange keine diagnostischen Konsequenzen aus den Ergebnissen der Blutproben entstehen. Sobald bei PatientInnen im Intensivierten Nachsorge Arm weitere Maßnahmen wie eine Bildgebung bei auffälligen Biomarkern nötig werden, wird die Verblindung aufgehoben.<br>**Intensivierte Nachsorge:** Testung der Blutproben auf Tumormarker (CA27.29, CA125, CEA), CTC und ctDNA). Auffällige Befunde eines oder mehrerer der Biomarker (CA27.29 oder CA125 oder CEA oder CTC oder ctDNA) lösen die Bildgebung zur Metastasensuche aus.<br>**Tumormarker:** Zur Bestimmung des Ausgangswertes (Baseline) der Tumormarker werden nach der Randomisierung zwei Blutproben im Abstand von 28 ± 5 Tagen abgenommen, wobei der Durchschnitt der jeweiligen Werte als Ausgangswert für den entsprechenden Tumormarker gilt. Als auffälliger Befund wird ein Anstieg des entsprechenden Tumormarkers von diesem Ausgangswert definiert (CA27.29 +75% oder CEA +100% oder CA125 +150%).<br>Wenn sich der auffällige Befund zeigt, wird eine Bildgebung zum Ausschluss von Metastasen veranlasst, welche analog der nationalen Leitlinien aus mindestens einer Computertomographie von Brust- und Bauchraum (CT Thorax und Abdomen) sowie einem Knochenszintigramm besteht. Sollte sich der Verdacht auf Metastasen nicht bestätigen, werden die StudienteilnehmerInnen weiterhin mittels Liquid-Biopsy in den regulären Studienvisiten überwacht. Sollte die auffällige Tumormarker-Erhöhung fortbestehen, sollten maximal drei CT-Untersuchungen nacheinander ausgeführt werden. Danach ist eine erneute Erhöhung der Tumormarker um die oben genannten definierten Anstiege nötig, um eine erneute Bildgebung zu initiieren. Das minimale Intervall zwischen zwei CT scans sollte mindestens 3 Monate und zwischen zwei SPECT Knochen scans mindestens 6 Monate betragen.<br>**CTC:** Bei erstmalig auffälligem Anstieg der Tumormarker wird eine zusätzliche Analyse auf CTCs zum Zeitpunkt der nächsten Visite durchgeführt. Zudem wird

	bei allen PatientInnen im Intensivierten Nachsorge-Arm ein Jahr nach Studieneinschluss eine Testung auf CTCs durchgeführt. Sollte ≥ 1 CTC nachgewiesen werden (unabhängig von den anderen Biomarkern), wird eine Bildgebung zur Metastasensuche initiiert. Sollten sich in der Bildgebung keine Metastasen zeigen, wird die PatientIn die Testung auf Biomarker (Tumormarker und ctDNA) im Blut in denselben Intervallen wie zuvor fortführen. Das minimale Intervall zwischen zwei CT scans sollte mindestens 3 Monate und zwischen zwei SPECT Knochen scans mindestens 6 Monate betragen.<br>**ctDNA:** Bei einem Nachweis von ctDNA wird eine Bildgebung zur Metastasensuche initiiert. Sollten in der Bildgebung keine Metastasen nachweisbar sein, wird die Bestimmung der ctDNA in denselben Intervallen wie zuvor durchgeführt. Wenn ctDNA weiterhin nachweisbar ist, dann sollten maximal drei aufeinander folgende CTs im dreimonatlichen Intervall durchgeführt werden. Danach wird sich das Kontrollintervall mittels Bildgebung auf alle sechs Monate für weitere drei aufeinander folgende Zeitpunkte erweitert. Sollte weiterhin ctDNA nachweisbar sein, ohne dass ein Rezidiv in der Bildgebung detektiert wird, wird das Kontrollintervall mittels Bildgebung auf einmal jährlich erweitert. Das minimale Intervall zwischen zwei CT scans sollte mindestens 3 Monate und zwischen zwei SPECT Knochen scans mindestens 6 Monate betragen.<br>**Standard Nachsorge:** Die entnommenen Blutproben werden nicht im Rahmen dieser Studie analysiert und werden daher keinen Einfluss auf die Initiierung einer Bildgebung zur Metastasensuche haben. Für diese Proben wird eine Biobank eingerichtet, welche retrospektive und translationale Forschung ermöglichen soll.<br>**Dauer der Intervention pro PatientIn:** 5 Jahre.<br>Die PatientInnen werden in einem Verhältnis von 1:1 in entweder auf den Intensivierten oder den Standard Nachsorge Arm verteilt. Diese Zufallsverteilung wird zunächst für die PatientInnen und die Studienzentren verblindet sein. Eine Entblindung erfolgt nur indirekt, wenn durch auffällige Befunde der analysierten Blutproben in der intensivierten Nachsorge Gruppe weitere Maßnahmen wie eine Bildgebung erforderlich werden.
**Primäre Zielsetzung und Endpunkte**	**Zwei primäre Endpunkte werden untersucht:**<br>**Gesamtüberleben (Overall Survival – OS)**<br>Zielsetzung:<br>Vergleich des Gesamtüberlebens nach fünf Jahren in dem Standard Nachsorge Arm mit dem Intensivierten Nachsorge Arm.<br>Endpunkt:<br>Das Gesamtüberleben ist definiert als die Zeit ab Randomisierung bis zum Tod der PatientIn, unabhängig von der Todesursache. Wenn einE PatientIn gestorben ist, wird das Gesamtüberleben zum Zeitpunkt des letzten Kontaktes zensiert.

	**Effekt der Gesamt-Vorlaufzeit (Overall Lead Time Effect)**<br><u>Zielsetzung:</u><br>Bestimmung einer Vorlaufzeit (Lead-Time), welche durch Tumormarker / CTC /ctDNA bei der Intensivierten Nachsorge im Vergleich zur Standardnachsorge nach Beendigung der Primärtherapie bei BrustkrebspatientInnen generiert wird.<br><u>Endpunkt:</u><br>Dieser Endpunkt ist ein zusammengesetzter Wert, welcher definiert wird als die Vorlaufzeit des Medians der Zeit vom molekularen bis mittels Bildgebung bestätigtem Rezidiv (welcher nur im Arm mit Intensivierter Nachsorge berechnet werden kann, siehe Definition bei Sekundären Endpunkten) plus den Unterschied zwischen der Zeit bis zur Fernmetastasierung in beiden Armen (d.h. der Unterschied zwischen dem Median der Zeit ab Randomisierung bis zur Fernmetastasierung bei allen PatientInnen mit Fernmetastasierung im Standard Nachsorge Arm sowie dem Median der Zeit ab Randomisierung bis zur Fernmetastasierung bei allen PatientInnen mit Fernmetastasierung im Intensivierten Nachsorge Arm). Dieser Effekt der Vorlaufzeit wird für alle Biomarker zusammen sowie, soweit anwendbar, für die einzelnen Biomarker (Tumormarker/CTC/ctDNA) separat bestimmt (d.h. Berechnung des molekularen Rückfalls basierend auf das Ergebnis nur eines dieser Marker).
**Sekundäre Zielsetzung und Endpunkte**	**Invasives krankheitsfreies Überleben (Invasive disease-free survival - IDFS)**<br><u>Zielsetzung:</u><br>Vergleich des invasiven krankheitsfreien Überlebens zwischen den beiden Studienarmen.<br><u>Endpunkt:</u><br>IDFS ist definiert als die Zeit zwischen Randomisierung bis zum ersten IDFS Ereignis, welches sowohl jegliches invasives ipsilaterales, regionales sowie kontralaterales Rezidiv als auch eine Fernmetastasierung, sekundäre primäre Tumorerkrankungen oder Tod unabhängig von der Todesursache beinhaltet. Ausgenommen hiervon sind nicht invasive in-situ Krebsarten. Wenn einE PatientIn kein solches Ereignis hatte, dann ist IDFS zum Zeitpunkt der letzten adäquaten Tumoreinschätzung zensiert.<br><br>**Fernerkrankungs-freies Überleben (Distant disease-free survival - DDFS)**<br><u>Zielsetzung:</u><br>Vergleich von DDFS zwischen den beiden Studienarmen.<br><u>Endpunkt:</u><br>DDFS ist definiert als Zeit zwischen Randomisierung bis zum ersten DDFS Ereignis, welches Metastasen, sekundäre primäre Tumorerkrankungen und Tod unabhängig von der Todesursache beinhaltet. Wenn einE PatientIn kein solches

Ereignis hatte, dann ist DDFS zum Zeitpunkt der letzten adäquaten Tumoreinschätzung zensiert.

Fernrezidiv-freies Überleben (Distant recurrence-free survival - DRFS)
Zielsetzung:
Vergleich von DRFS zwischen den beiden Studienarmen.
Endpunkt:
DRFS ist definiert als Zeit zwischen Randomisierung bis zum ersten DRFS Ereignis, welches Metastasen und sekundäre primäre Tumorerkrankungen beinhaltet. Tod unabhängig von der Todesursache wird nicht als Ereignis gezählt. Wenn einE PatientIn kein solches Ereignis hatte, dann ist DRFS zum Zeitpunkt der letzten adäquaten Tumoreinschätzung zensiert.

Brustkrebs-spezifisches Überleben (Breast cancer specific survival - BCSS)
Zielsetzung:
Vergleich von BCSS zwischen den beiden Studienarmen.
Endpunkt:
BCSS ist definiert als Zeit zwischen Randomisierung bis zum Brustkrebs-assoziierten Tod. Wenn einE PatientIn nicht gestorben ist, dann ist BCSSS zum Zeitpunkt des letzten PatientInnenkontaktes zensiert. Wenn einE PatientIn aus anderen Gründen gestorben ist, welche nicht in Zusammenhang mit der Brustkrebserkrankung stehen (bestimmt durch die klinische Einschätzung), dann ist BCSS zum Todestag zensiert.

Invasives Brustkrebs-freies Überleben (Invasive breast cancer free survival - IBCFS)
Zielsetzung:
Vergleich von IBCFS zwischen den beiden Studienarmen.
Endpunkt:
IBCFS ist definiert als Zeit zwischen Randomisierung bis zum ersten IBCFS Ereignis, welches sowohl jegliches invasives ipsilaterales, regionales sowie kontralaterales Rezidiv als auch eine Fernmetastasierung oder Tod unabhängig von der Todesursache beinhaltet. Ausgenommen hiervon sind nicht invasive in-situ Karzinome. Wenn einE PatientIn kein solches Ereignis hatte, dann ist IBCFS zum Zeitpunkt der letzten adäquaten Tumoreinschätzung zensiert.

Gesamtüberleben (OS) nach 10 Jahren
Zielsetzung:
Vergleich der 10-Jahresüberlebensraten zwischen den beiden Studienarmen.
Endpunkt:
Das Gesamtüberleben ist definiert als die Zeit ab Randomisierung bis zum Tod der PatientIn, unabhängig von der Todesursache. Wenn einE PatientIn

	gestorben ist, wird das Gesamtüberleben zum Zeitpunkt des letzten Kontaktes zensiert.<br><br>**Molekulare bis bildgebend verifizierte Rückfall Vorlaufsrate (Lead-Time) im Intensivierten Nachsorgearm**<br>Zielsetzung:<br>Bestimmung der Vorlaufzeit (Median, Interquartile, Verteilung, 95% Konfidenzintervall), welche durch die Liquid Biopsy im Intensivierten Vorsorgearm generiert wird.<br>Endpunkt:<br>Die Vorlaufzeit (Lead Time) ist definiert als Zeit zwischen dem ersten molekularen Rückfall bis zum bildgebend verifizierten Rezidiv im Sinne einer Fernmetastasierung bei allen PatientInnen im Intensivierten Narchsorge Arm, bei welchen ein bildgebend verifiziertes Rezidiv innerhalb der fünfjährigen Interventionsphase dokumentiert wurde.<br>Wenn einE PatientIn ein mittels Bildgebung diagnostiziertes Rezidiv ohne molekulares Rezidiv hatte, wird die Vorlaufzeit dieser PatientIn auf „0“ gesetzt. Dieser sekundäre Endpunkt wird für alle Marker als Kombination sowie für die einzelnen Marker ausgewertet.<br><br>**Lebensqualität mittels der Fragebögen EORTC QLQ-C30 und PA-F12 (Quality of Life – QoL)**<br>Zielsetzung:<br>Vergleich der Lebensqualität zwischen dem Intensivierte Nachsorge Arm und dem Standard Nachsorge Arm.<br>Endpunkt:<br>Die Lebensqualität wird in beiden Armen mittels zweier Fragebögen (EORTC QLQ-C30 and PA-F12) alle sechs Monate in der fünfjährigen Interventionsphase erfasst sowie einmal im Jahr in der fünfjährigen Nachbeobachtungsphase. Die Daten können mittels Papierversionen oder digital im Rahmen einer Digital Health Application erfasst werden.<br><br>**Liquid biopsy Sensitivität (CA27.29, CEA, CA125, CTC und ctDNA)**<br>Zielsetzung:<br>Bestimmung der Sensitivität (d.h. die wahre Positivrate) der Liquid Biopsy Biomarker im Intensivierten Nachsorge Arm (in Kombination oder einzeln).<br>Endpunkt:<br>Anteil der PatientInnen im Intensivierten Nachsorge Arm mit bildgebend bestätigtem Rezidiv in der fünfjährigen Interventionsphase mit einem auffälligen Biomarkerergebnis (d.h. molekularen Rückfall) innerhalb von 36 Monaten bevor das bestätigte Rezidiv diagnostiziert wurde (oder innerhalb von

	60 Monaten bevor das Rezidiv bestätigt wurde, wenn innerhalb der ersten 36 Monate mindestens ein Jahr ein positives Biomarkerergebnis vorlag).<br><br>**Liquid biopsy Spezifität (CA27.29, CEA, CA125, CTC und ctDNA)**<br>Zielsetzung:<br>Bestimmung der Spezifität (d.h. die wahre Negativrate) der Liquid Biopsy Biomarker im Intensivierten Nachsorge Arm (in Kombination oder einzeln).<br>Endpunkt:<br>Anteil der PatientInnen im Intensivierten Nachsorge Arm mit keinem bildgebend bestätigten Rezidiv in der fünfjährigen Interventionsphase, die kein auffälliges Biomarkerergebnis hatten (d.h. kein molekularer Rückfall) innerhalb von 36 Monaten vor Ende der fünfjährigen Interventionsphase.<br><br>**Liquid Biopsy Falsch-Positiv Rate (CA27.29, CEA, CA125, CTC und ctDNA)**<br>Zielsetzung:<br>Bestimmung der Falsch-Positiv Rate der genutzten Liquid Biopsy Biomarker im Intensivierten Nachsorge Arm (in Kombination oder einzeln).<br>Endpunkt:<br>Anteil aller PatientInnen mit ausschließlich auffälligen Biomarker-Ergebnissen (somit Anhalt für einen molekularen Rückfall), die innerhalb der fünfjährigen Interventionsphase kein bildgebend nachgewiesenes Rezidiv hatten, welches innerhalb von 36 Monaten nach dem ersten auffälligen Biomarker-Ergebnis auftrat.<br><br>**Liquid Biopsy Falsch-Negativ Rate (CA27.29, CEA, CA125, CTC und ctDNA)**<br>Zielsetzung:<br>Bestimmung der Falsch-Negativ Rate der genutzten Liquid Biopsy Biomarker im Intensivierten Nachsorge Arm (in Kombination oder einzeln).<br>Endpunkt:<br>Anteil aller PatientInnen mit ausschließlich unauffälligen Biomarkerergebnissen (somit kein Anhalt für einen molekularen Rückfall), die ein bildgebend nachgewiesenes Rezidiv innerhalb der fünfjährigen Interventionsphase hatten.<br><br>**Häufigkeit der Positivität der Liquid Biopsy Biomarker (CA27.29, CEA, CA125, CTC und ctDNA)**<br>Zielsetzung:<br>Bestimmung der gesamten Häufigkeit von positiven Liquid Biopsy Biomarkern im Intensivierten Nachsorge Arm (in Kombination oder einzeln).<br>Endpunkt:<br>Anzahl aller PatientInnen im Intensivierten Nachsorge Arm, die mindestens ein positives Biomarkerergebnis (entsprechend eines molekularen Rückfalls) in dem Zeitraum der fünfjährigen Interventionsphase hatten.

**Translationale Forschung**	<u>Zielsetzung:</u><br>Eine Biobank wird aufgebaut, um die Möglichkeit von retrospektiven Analysen während und nach der Studie zu ermöglichen.<br><u>Endpunkt:</u><br>Eine Biobank wird zur Aufbewahrung von ungenutzten Materialien von beiden Studienarmen genutzt, um zukünftige retrospektive Analysen und Studien zu ermöglichen.
**Einschlusskriterien**	Ein Einschluss von PatientInnen ist nur möglich, wenn alle nachfolgenden Kriterien erfüllt werden:<br>1. Unterschriebene Einverständniserklärung für alle Studienmaßnahmen analog der bestehenden lokalen regulatorischen Anforderungen bevor Studien-spezifische Maßnahmen stattfinden.<br>2. Primärer, unilateraler oder bilateraler, histologisch gesicherter Brustkrebs.<br>3. PatientInnen mit einem mittleren bis hohen Rezidivrisiko, definiert als eines der folgenden Kriterien<br>- Indikation für (neo-)adjuvante Chemotherapie (unabhängig davon, ob diese durchgeführt wurde) oder<br>- Großer Tumor (> 50 mm), oder<br>- Positive Lymphknoten (> pN1mi), oder<br>- Hohes Grading (G3).<br>4. Komplettresektion des Primärtumors mit freien Schnitträndern.<br>5. Beendigung der primären Tumortherapie (adjuvante Chemotherapie, Operation oder Strahlentherapie, je nachdem was als Letztes stattfand) maximal vor 24 Monaten. Ein Einschluss von PatientInnen unter adjuvanter Therapie, welche keine Chemotherapie ist (zum Beispiel antihormonelle Therapie, Antikörper-Therapie, Therapie mit CDK4/6-Inhibitoren, PARP-Inhibitoren oder PI3K Inhibitoren, sowie Antikörper-Drug-Konjugate oder andere neue Substanzen) ist erlaubt. PatientInnen mit luminalem A/B Brustkrebs (ER/PGR positiv, HER2 negative/low) dürfen bis zu 60 Monate nach beendeter Primärtherapie eingeschlossen werden.<br>6. Verfügbarkeit von Primärtumor-Gewebe (entweder als FFPE Glasträger (≥ 6 mm³, mind. 10 Träger, Dicke: 5 µm-10 µm, Oberfläche >150 mm² und 1 H&E gefärbter Träger, mind. 20% Tumorgehalt) oder FFPE Block (≥ 6 mm³ Dicke: 100 µm, Oberfläche: >150 mm² und 1 H&E gefärbter Träger, mind. 20% Tumorgehalt) oder genomische DNA extrahiert von FFPE Trägern oder Block (≥ 600 ng, Mindestvolumen: 25 µL, Konzentration: 20 ng/µL, Buffer: 10 mM Tris pH 8, 1 mM EDTA)) zum Zeitpunkt des Studieneinschlusses<br>- PatientInnen mit primär systemischer Therapie: Tumorgewebe aus der initialen Biopsie bei Erstdiagnose

	- PatientInnen mit primär operativer Therapie: chirurgisch entferntes Tumorgewebe.<br>Wichtig: Bei PatientInnen mit bilateralem Mammakarzinom ist Gewebe von beiden Seiten erforderlich!<br>7. Kein klinischer Hinweis auf eine Fernmetastasierung (cM0).<br>8. Frauen oder Männer ≥ 18 Jahre und ≤ 75 Jahre.<br>9. Guter Allgemeinzustand, gemessen als Status ≤ 1 auf der Eastern Cooperative Oncology Group (ECOG) Skala.<br>10. Bereitschaft der PatientInnen zur Einhaltung der geplanten Studienvisiten, Blutabnahmen, Diagnostik sowie weiterer Maßnahmen im Rahmen der Studie.
**Ausschlusskriterien**	Ein Einschluss von PatientInnen ist nur möglich, wenn keines der folgenden Kriterien zutrifft:<br>1. PatientInnen mit einer weiteren Krebserkrankung in der Vorgeschichte, mit Ausnahme der folgenden Erkrankungen:<br>- in situ Karzinom des Gebärmutterhalses<br>- adäquat behandeltes Basalzellkarzinom der Haut<br>- ipsi- oder kontralaterales non-invasives Karzinom der Brust (DCIS).<br>2. Patientinnen in Schwangerschaft oder Stillzeit.<br>3. Vorgeschichte einer signifikanten neurologischen oder psychiatrischen Erkrankung, einschließlich psychiatrischer Störungen, Demenz oder Krampfanfälle, die an dem Verständnis oder dem Einverständnis der Patientin zweifeln lassen.<br>4. Niereninsuffizienz mit GFR < 30 mL/min.<br>5. Vorausgehende oder begleitende zytostatische oder andere systemische Behandlung, welche nicht der Behandlung des primären Brustkrebses dient.
**Bestimmung der PatientInnenzahl**	Die SURVIVE-Studie ist eine parallel-zweiarmig randomisierte, teilweise doppelt-verblindete Überlegenheitsstudie. Um eine 2,35%-ige Verbesserung der 5-Jahres Gesamtüberlebensrate (OS-Rate) von 92,65% im Standard-Nachsorgearm auf 95% im Intensivierten Nachsorgearm zu detektieren, entsprechend einer Hazard Ratio von 0,672, erfordert es einen Einschluss von ungefähr 3500 PatientInnen (1750 in jedem der Arme) um eine 80% Power bei einem zwei-seitigen Signifikanzniveau von 5% zu erreichen, vorausgesetzt einer einheitlichen Rekrutierung über 2 Jahre, eine Drop-Out Rate von 15% in beiden Studienarmen und eine Gesamt-Studiendauer von 10 Jahren.<br>Der Referenzwert der 5-Jahres Gesamt-Überlebensrate von 92,65% basiert auf Daten der randomisierten Phase III Studie Success A (Clinicaltrials.gov NCT02181101), aus welcher die 5-Jahres Überlebensraten für 3754 Mittel- bis Hochrisiko BrustkrebspatientInnen vor kurzem publiziert wurden.

**Randomisation**	Die Verteilung der StudienteilnehmerInnen auf die Studienarme wird zentral mittels permuted block list randomization vorgenommen, wenn die PatientInnen die Ein- und Ausschlusskriterien erfüllen.
**Faktoren zur Stratifikation**	Folgende Stratifikationsfaktoren werden genutzt:<br>• Hormonrezeptor Status (negativ vs. positiv)<br>• HER2 Status (negativ vs. positiv)<br>• Histologischer Lymphknotenstatus zum Zeitpunkt der Operation ((y)pN0 vs. (y)pN+)
**Statistische Methoden:**	Studienpopulation:<br>Die folgenden Sets zur Studienpopulation werden vorgenommen:<br>• Intention to Treat (ITT) Set: Alle randomisierten Patienten mit unterschriebener Einverständniserklärung.<br>• Per Protocol (PP) Set: Alle Patienten des ITT Sets, welche weder Ein- noch Ausschlusskriterien verletzt haben und streng nach Protokoll behandelt wurden.<br><br>Falls nötig werden weitere Datensets für zusätzliche Analysen im separaten Analyseplan weiter ausgeführt.<br><br>**Analyse des primären Endpunktes und der Studienpopulation:**<br>Der primäre Endpunkt des Gesamtüberlebens wird mittels ITT Set analysiert, geschätzt nach der Kaplan Meier Produktgrenzen Methode, und 5-Jahresüberlebensraten, 95% Konfidenzintervalle und Überlebensplots werden zur Verfügung gestellt. Das Gesamtüberleben wird zwischen den Patienten der beiden Studienarme mittels Log-rank Test verglichen, zudem werden univariable Cox Regressionen sowie zusätzliche multivariable Analysen adjustiert an andere Faktoren genutzt, unter Nutzung von passenden Regressionsmodellen (cox proportional hazard regression model). Hazard ratios und die entsprechenden 95% Konfidenzintervalle sowohl der univariablen als auch der adjustierten multivariablen Analysen werden berichtet.<br>Der zusätzliche Endpunkt der Overall Lead Time Effect wird wie unter dem Abschnitt bereits beschrieben berechnet. Hierbei handelt es sich um einen deskriptiven zusammengesetzten Wert, welcher aus zwei zusammenhanglosen Medianen besteht, die für verschiedene Patientenkohorten gelten. Es werden keine Konfidenzintervalle angegeben.<br><br>**Analyse der sekundären Endpunkte:**<br>Alle Analysen der sekundären Zielsetzungen werden explorative sein. Alle sekundären Endpunkte und andere Ergebnisse, die auf Häufigkeiten basieren, werden mittels Häufigkeitstabellen zusammengerechnet, welche absolute und relative Häufigkeiten mit entsprechenden Konfidenzintervallen darstellen werden. Gruppenvergleiche werden mittels entsprechender nicht

	parametrischer statistischer Tests wie dem exakten Fisher-Test, Chi-Quadrat Test oder dem Cochran-Mantel-Haenszel Test. Kontinuierliche Variabeln werden mittels deskriptiver Statistik zusammengefasst (Anzahl von gültigen und fehlenden Beobachtungen, Durchschnitt, Standardabweichung, Median, Interquartilen, Minimum und Maximum) und werden zwischen den Armen mittels entsprechender nicht parametrischer Tests wie Wilcoxon-Mann-Whitney verglichen. Die sekundären Endpunkte, welches ich auf das Überleben beziehen (IDFS; DDFS; DRFS; BCSS) werden auf dieselbe Art und Weise wie der primäre Endpunkt des Gesamtüberlebens analysiert. Die Level der Tumormarker (CA27.29, CEA, CA125) sowie das Vorkommen und die Anzahl von CTCs und die Anwesenheit von ctDNA, welche zu unterschiedlichen Zeitpunkten besteht, wird deskriptiv ausgewertet. Zudem werden die temporären Veränderungen beschrieben und mittels entsprechender statistischer Methoden analysiert (z.B. verallgemeinerte lineare gemischte Modelle).<br>Die Daten zur Lebensqualität werden nach der Anleitung der entsprechenden validierten Fragebögen (EORTC QLQ-C30 und PA-F12) analysiert. Zusätzliche explorative Analysen wie zum Beispiel Subgruppen-Analysen werden entsprechend stattfinden.<br>Für weitere Details der statistischen Analyse kann der Statistische Analyse Plan (SAP) eingesehen werden, welcher vor Verschluss der Datenbank fertig gestellt wird.
**Interimsanalyse**	Es ist keine formale Interimsanalyse für Effektivität vorgesehen.<br>Eine Interimsanalyse bezogen auf Lebensqualität ist im Monat 40 geplant.<br>Eine Analyse zur Überprüfung der Sinnhaftigkeit der ctDNA Bestimmung ist geplant, nähere Details sind im Statistik Kapitel des Protokolls beschrieben.
**Biomaterial**	- Gewebe vom Primärtumor (entweder als FFPE Glasträger (≥ 6 mm³, mind. 10 Träger, Dicke: 5 µm-10 µm, Oberfläche >150 mm² und 1 H&E gefärbter Träger, mind. 20% Tumorgehalt) oder FFPE Block (≥ 6 mm³ Dicke: 100 µm, Oberfläche: >150 mm² und 1 H&E gefärbter Träger, mind. 20% Tumorgehalt) oder genomische DNA extrahiert von FFPE Trägern oder Block (≥ 600 ng, Mindestvolumen: 25 µL, Konzentration: 20 ng/µL, Buffer: 10 mM Tris pH 8, 1 mM EDTA)) zum Zeitpunkt des Studieneinschlusses<br>▪ PatientInnen mit primär systemischer Therapie: Tumorgewebe aus der initialen Biopsie bei Erstdiagnose<br>▪ PatientInnen mit primär operativer Therapie: chirurgisch entferntes Tumorgewebe.<br><br>Wichtig: Bei PatientInnen mit bilaterealem Mammakarzinom ist Gewebe von beiden Seiten erforderlich!<br><br>- Zwei Streck Cell-Free DNA BCT® Röhrchen (je 10 mL Blut) für ctDNA Analyse zum Zeitpunkt des Studieneinschlusses sowie alle drei Monate für die

	ersten drei Jahre und alle 6 Monate für die folgenden zwei Jahre; insgesamt über einen Zeitraum von fünf Jahren.<br>- Eine Serummonovette (7,5 mL Blut) zur Bestimmung von Tumormarkern zum Zeitpunkt des Studieneinschlusses sowie alle drei Monate für die ersten drei Jahre und alle 6 Monate für die folgenden zwei Jahre; insgesamt über einen Zeitraum von fünf Jahren.<br>- Drei CellSave Röhrchen (je 10 mL Blut) zur Bestimmung von CTCs ein Jahr nach Studieneinschluss. Zudem wird bei dem ersten Nachweis von auffälligen Tumormarkern eine zusätzliche Blutprobe zur CTC-Bestimmung entnommen.<br><br>Die Blutproben des Standard Nachsorge Arms werden nicht direkt analysiert, sondern in einer Biobank für retrospektive und translationale zukünftige Studien aufbewahrt.
**Einschlusszeitraum**	Ca. 24 Monate.
**Nachbeobachtung**	Nach der interventionellen Phase der Studie, welche mit der letzten Blutprobe nach insgesamt 5 Jahren abgeschlossen ist, schließt sich eine Nachbeobachtungsperiode von weiteren fünf Jahren an, ohne dass Studien-spezifische Interventionen stattfinden. Die gesammelten Daten werden mittels digitaler Gesundheitsapplikation, Telefonanrufen oder in Papierform abgefragt. Die Daten, welche in den Nachbeobachtungs-Visiten abgefragt werden, werden im Studienprotokoll näher beschrieben.<br>Nach Ende der Studie haben die teilnehmenden PatientInnen die Möglichkeit zur Erhebung von Langzeitdaten die Patientenselbstauskunft zu nutzen.
**Anzahl der Studienzentren**	Es ist geplant, dass die Studie an insgesamt ungefähr 150 Zentren stattfindet, welche Teil der GBG, AGO B Studiengruppe sowie Teil der SUCCESS und DETECT Studiengruppen sind. Das Projekt wird von PatientInnen Interessensgruppen unterstützt (mamazone e.V., PATH-Foundation, Brustkrebs Deutschland e.V., Frauenselbsthilfe Krebs e.V., MammaMia!).
**Zeitplan:**	Einschluss erster PatientInnen Q4/2022<br><br>Einschluss letzter PatientInnen Q4/2024<br><br>Ende der interventionellen Phase Q4/2029<br>Ende der Studie Q4/2035

27 REFERENCES

1. GLOBOCAN 2020: new global cancer data: https://www.uicc.org/news/globocan-2020-new-global-cancer-data.
2. Global Burden of Disease Cancer Collaboration, Fitzmaurice C, Akinyemiju TF, Al Lami FH, Alam T, Alizadeh-Navaei R, u. a. Global, Regional, and National Cancer Incidence, Mortality, Years of Life Lost, Years Lived With Disability, and Disability-Adjusted Life-Years for 29 Cancer Groups, 1990 to 2016: A Systematic Analysis for the Global Burden of Disease Study. JAMA Oncology. 1. November 2018;4(11):1553.
3. Sun L, Legood R, dos-Santos-Silva I, Gaiha SM, Sadique Z. Global treatment costs of breast cancer by stage: A systematic review. Diaby V, Herausgeber. PLOS ONE. 26. November 2018;13(11):e0207993.
4. Ginsburg O, Bray F, Coleman MP, Vanderpuye V, Eniu A, Kotha SR, u. a. The global burden of women's cancers: a grand challenge in global health. The Lancet. Februar 2017;389(10071):847–60.
5. Liberati A. The GIVIO trial on the impact of follow-up care on survival and quality of life in breast cancer patients. Annals of Oncology. 1. Januar 1995;6(suppl 2):S41–6.
6. Del Turco MR. Intensive Diagnostic Follow-up After Treatment of Primary Breast Cancer: A Randomized Trial. JAMA. 25. Mai 1994;271(20):1593.
7. Gao JJ, Cheng J, Prowell TM, Bloomquist E, Tang S, Wedam SB, u. a. Overall survival in patients with hormone receptor-positive, HER2-negative, advanced or metastatic breast cancer treated with a cyclin-dependent kinase 4/6 inhibitor plus fulvestrant: a US Food and Drug Administration pooled analysis. Lancet Oncol. November 2021;22(11):1573–81.
8. Swain SM, Miles D, Kim SB, Im YH, Im SA, Semiglazov V, u. a. Pertuzumab, trastuzumab, and docetaxel for HER2-positive metastatic breast cancer (CLEOPATRA): end-of-study results from a double-blind, randomised, placebo-controlled, phase 3 study. Lancet Oncol. April 2020;21(4):519–30.
9. Murthy RK, Loi S, Okines A, Paplomata E, Hamilton E, Hurvitz SA, u. a. Tucatinib, Trastuzumab, and Capecitabine for HER2-Positive Metastatic Breast Cancer. N Engl J Med. 13. Februar 2020;382(7):597–609.
10. Robson ME, Tung N, Conte P, Im SA, Senkus E, Xu B, u. a. OlympiAD final overall survival and tolerability results: Olaparib versus chemotherapy treatment of physician's choice in patients with a germline BRCA mutation and HER2-negative metastatic breast cancer. Ann Oncol. 1. April 2019;30(4):558–66.
11. Schmid P, Rugo HS, Adams S, Schneeweiss A, Barrios CH, Iwata H, u. a. Atezolizumab plus nab-paclitaxel as first-line treatment for unresectable, locally advanced or metastatic triple-negative breast cancer (IMpassion130): updated efficacy results from a randomised, double-blind, placebo-controlled, phase 3 trial. Lancet Oncol. Januar 2020;21(1):44–59.
12. Bardia A, Mayer IA, Vahdat LT, Tolaney SM, Isakoff SJ, Diamond JR, u. a. Sacituzumab Govitecan-hziy in Refractory Metastatic Triple-Negative Breast Cancer. N Engl J Med. 21. Februar 2019;380(8):741–51.
13. Jung SY, Sereika SM, Linkov F, Brufsky A, Weissfeld JL, Rosenzweig M. The effect of delays in treatment for breast cancer metastasis on survival. Breast Cancer Res Treat. Dezember 2011;130(3):953–64.
14. Moschetti I, Cinquini M, Lambertini M, Levaggi A, Liberati A. Follow-up strategies for women

treated for early breast cancer. Cochrane Database Syst Rev. 27. Mai 2016;(5):CD001768.
15. Lee JS, Park S, Park JM, Cho JH, Kim SI, Park BW. Elevated levels of preoperative CA 15-3 and CEA serum levels have independently poor prognostic significance in breast cancer. Annals of Oncology. Mai 2013;24(5):1225–31.
16. Kumpulainen EJ, Keskikuru RJ, Johansson RT. Serum tumor marker CA 15.3 and stage are the two most powerful predictors of survival in primary breast cancer. Breast Cancer Res Treat. November 2002;76(2):95–102.
17. Robertson JF, Jaeger W, Syzmendera JJ, Selby C, Coleman R, Howell A, u. a. The objective measurement of remission and progression in metastatic breast cancer by use of serum tumour markers. European Group for Serum Tumour Markers in Breast Cancer. Eur J Cancer. Januar 1999;35(1):47–53.
18. Hepp P, Andergassen U, Jäger B, Trapp E, Alunni-Fabbroni M, Friedl TWP, u. a. Association of CA27.29 and Circulating Tumor Cells Before and at Different Times After Adjuvant Chemotherapy in Patients with Early-stage Breast Cancer - The SUCCESS Trial. Anticancer Res. 2016;36(9):4771–6.
19. Huebner H, Häberle L, Müller V, Schrader I, Lorenz R, Forstbauer H, u. a. MUC1 (CA27.29) before and after Chemotherapy and Prognosis in High-Risk Early Breast Cancer Patients. Cancers (Basel). 28. März 2022;14(7):1721.
20. Harris L, Fritsche H, Mennel R, Norton L, Ravdin P, Taube S, u. a. American Society of Clinical Oncology 2007 Update of Recommendations for the Use of Tumor Markers in Breast Cancer. Journal of Clinical Oncology. 20. November 2007;25(33):5287–312.
21. Tormey DC, Waalkes TP. Clinical correlation between CEA and breast cancer. Cancer. September 1978;42(3 Suppl):1507–11.
22. Van Poznak C, Somerfield MR, Bast RC, Cristofanilli M, Goetz MP, Gonzalez-Angulo AM, u. a. Use of Biomarkers to Guide Decisions on Systemic Therapy for Women With Metastatic Breast Cancer: American Society of Clinical Oncology Clinical Practice Guideline. JCO. 20. August 2015;33(24):2695–704.
23. Lauro S, Trasatti L, Bordin F, Lanzetta G, Bria E, Gelibter A, u. a. Comparison of CEA, MCA, CA 15-3 and CA 27-29 in follow-up and monitoring therapeutic response in breast cancer patients. Anticancer Res. August 1999;19(4C):3511–5.
24. Ertl I, Heinemann V, Laessig D, Nagel D, Seidel D, Stieber P. CA 125 in the early detection of metastatic breast cancer. JCO. 20. Mai 2009;27(15_suppl):e12015–e12015.
25. Jensen JL, Maclean GD, Suresh MR, Almeida A, Jette D, Lloyd S, u. a. Possible utility of serum determinations of CA 125 and CA 27.29 in breast cancer management. Int J Biol Markers. März 1991;6(1):1–6.
26. Di Gioia D, Blankenburg I, Nagel D, Heinemann V, Stieber P. Tumor markers in the early detection of tumor recurrence in breast cancer patients: CA 125, CYFRA 21-1, HER2 shed antigen, LDH and CRP in combination with CEA and CA 15-3. Clinica Chimica Acta. Oktober 2016;461:1–7.
27. Berruti A, Tampellini M, Torta M, Buniva T, Gorzegno G, Dogliotti L. Prognostic value in predicting overall survival of two mucinous markers: CA 15-3 and CA 125 in breast cancer patients at first relapse of disease. European Journal of Cancer. Januar 1994;30(14):2082–4.
28. Norum LF, Erikstein B, Nustad K. Elevated CA125 in breast cancer--A sign of advanced disease. Tumour Biol. August 2001;22(4):223–8.
29. Stieber P, Nagel D, Blankenburg I, Heinemann V, Untch M, Bauerfeind I, u. a. Diagnostic efficacy of CA 15-3 and CEA in the early detection of metastatic breast cancer—A retrospective analysis

of kinetics on 743 breast cancer patients. Clinica Chimica Acta. August 2015;448:228–31.
30. Di Gioia D, Stieber P, Schmidt GP, Nagel D, Heinemann V, Baur-Melnyk A. Early detection of metastatic disease in asymptomatic breast cancer patients with whole-body imaging and defined tumour marker increase. Br J Cancer. März 2015;112(5):809–18.
31. Lin DC, Genzen JR. Concordance analysis of paired cancer antigen (CA) 15-3 and 27.29 testing. Breast Cancer Res Treat. Januar 2018;167(1):269–76.
32. Klee GG, Schreiber WE. MUC1 gene-derived glycoprotein assays for monitoring breast cancer (CA 15-3, CA 27.29, BR): are they measuring the same antigen? Arch Pathol Lab Med. Oktober 2004;128(10):1131–5.
33. Fehm T, Braun S, Muller V, Janni W, Gebauer G, Marth C, u. a. A concept for the standardized detection of disseminated tumor cells in bone marrow from patients with primary breast cancer and its clinical implementation. Cancer. 1. September 2006;107(5):885–92.
34. Braun S, Vogl FD, Naume B, Janni W, Osborne MP, Coombes RC, u. a. A pooled analysis of bone marrow micrometastasis in breast cancer. N Engl J Med. 25. August 2005;353(8):793–802.
35. Zhang L, Riethdorf S, Wu G, Wang T, Yang K, Peng G, u. a. Meta-analysis of the prognostic value of circulating tumor cells in breast cancer. Clin Cancer Res. 15. Oktober 2012;18(20):5701–10.
36. Bidard FC, Peeters DJ, Fehm T, Nolé F, Gisbert-Criado R, Mavroudis D, u. a. Clinical validity of circulating tumour cells in patients with metastatic breast cancer: a pooled analysis of individual patient data. Lancet Oncol. April 2014;15(4):406–14.
37. Cristofanilli M, Budd GT, Ellis MJ, Stopeck A, Matera J, Miller MC, u. a. Circulating tumor cells, disease progression, and survival in metastatic breast cancer. N Engl J Med. 19. August 2004;351(8):781–91.
38. Rack BK, Schindlbeck C, Andergassen U, Schneeweiss A, Zwingers T, Lichtenegger W, u. a. Use of circulating tumor cells (CTC) in peripheral blood of breast cancer patients before and after adjuvant chemotherapy to predict risk for relapse: The SUCCESS trial. Journal of Clinical Oncology. 20. Mai 2010;28(15_suppl):1003–1003.
39. Trapp E, Janni W, Schindlbeck C, Jückstock J, Andergassen U, de Gregorio A, u. a. Presence of Circulating Tumor Cells in High-Risk Early Breast Cancer During Follow-Up and Prognosis. J Natl Cancer Inst. 1. April 2019;111(4):380–7.
40. Janni WJ, Rack B, Terstappen LWMM, Pierga JY, Taran FA, Fehm T, u. a. Pooled Analysis of the Prognostic Relevance of Circulating Tumor Cells in Primary Breast Cancer. Clin Cancer Res. 15. Mai 2016;22(10):2583–93.
41. Rack B, Schindlbeck C, Jückstock J, Andergassen U, Hepp P, Zwingers T, u. a. Circulating Tumor Cells Predict Survival in Early Average-to-High Risk Breast Cancer Patients. JNCI: Journal of the National Cancer Institute [Internet]. Mai 2014 [zitiert 23. Februar 2021];106(5). Verfügbar unter: https://academic.oup.com/jnci/article-lookup/doi/10.1093/jnci/dju066
42. The Cancer Genome Atlas Network. Comprehensive molecular portraits of human breast tumours. Nature. Oktober 2012;490(7418):61–70.
43. Leary RJ, Sausen M, Kinde I, Papadopoulos N, Carpten JD, Craig D, u. a. Detection of chromosomal alterations in the circulation of cancer patients with whole-genome sequencing. Sci Transl Med. 28. November 2012;4(162):162ra154.
44. Bettegowda C, Sausen M, Leary RJ, Kinde I, Wang Y, Agrawal N, u. a. Detection of circulating tumor DNA in early- and late-stage human malignancies. Sci Transl Med. 19. Februar 2014;6(224):224ra24.
45. Wan JCM, Heider K, Gale D, Murphy S, Fisher E, Mouliere F, u. a. ctDNA monitoring using patient-

specific sequencing and integration of variant reads. Sci Transl Med. 17. Juni 2020;12(548).
46. Garcia-Murillas I, Schiavon G, Weigelt B, Ng C, Hrebien S, Cutts RJ, u. a. Mutation tracking in circulating tumor DNA predicts relapse in early breast cancer. Sci Transl Med. 26. August 2015;7(302):302ra133.
47. Coombes RC, Page K, Salari R, Hastings RK, Armstrong A, Ahmed S, u. a. Personalized Detection of Circulating Tumor DNA Antedates Breast Cancer Metastatic Recurrence. Clin Cancer Res. 15. Juli 2019;25(14):4255–63.
48. Olsson E, Winter C, George A, Chen Y, Howlin J, Tang ME, u. a. Serial monitoring of circulating tumor DNA in patients with primary breast cancer for detection of occult metastatic disease. EMBO Mol Med. August 2015;7(8):1034–47.
49. Janni W. P144 A personalised sequencing approach for liquid biopsy-based detection of recurrent disease in early-stage breast cancer. Annals of Oncology. (32):S423.
50. Ho PJ, Cook AR, Binte Mohamed Ri NK, Liu J, Li J, Hartman M. Impact of delayed treatment in women diagnosed with breast cancer: A population-based study. Cancer Med. 2020;9(7):2435-2444. doi:10.1002/cam4.2830.
51. https://www.fda.gov/radiation-emitting-products/medical-x-ray-imaging/what-are-radiation-risks-ct (accessed on 16.11.2021).
52. McCollough CH, Bushberg JT, Fletcher JG, Eckel LJ. Answers to Common Questions About the Use and Safety of CT Scans. Mayo Clin Proc. 2015 Oct;90(10):1380-92. doi: 10.1016/j.mayocp.2015.07.011. PMID: 26434964.
53. https://www.leitlinienprogramm-onkologie.de/fileadmin/user_upload/Downloads/Leitlinien/Mammakarzinom_4_0/Version_4.0/LL_Mammakarzinom_Kurzversion_4.0.pdf (accessed 17.11.2021).
54. https://www.ago-online.de/fileadmin/ago-online/downloads/_leitlinien/kommission_mamma/2021/Einzeldateien/2021D_12_Neoadjuvante_systemische_Therapie_MASTER_final_20210301.pdf (accessed 17.11.2021).
55. https://www.ago-online.de/fileadmin/ago-online/downloads/_leitlinien/kommission_mamma/2021/Einzeldateien/2021D_16_Nachsorge_MASTER_final_20210302.pdf (accessed on 16.11.2021).
56. Leaflets for: CL AIA-PACK® 27.29 TEST CUP (EU rev. CL-27.29-020617), CL AIA-PACK® CEA TEST CUP (EU rev. CL-CEA-031118), CL AIA-PACK® OVCA TEST CUP (EU rev. OVCA-030918) for AIA®-CL1200 analyzer by TOSOH BIOSCIENCE (TOSOH CORPORATION, 3-8-2, Shiba-Koen, Minato-ku, Tokyo 105-8623 , Japan / TOSOH EUROPE N.V., Transportstraat 4, B-3980 Tessenderlo, Belgien), www.tosohbioscience.eu.
57. Heider K, Gale D, Ruiz-Valdepenas A, Marsico G, Sharma G, Perry M, u. a. Abstract 735: Sensitive detection of ctDNA in early stage non-small cell lung cancer patients with a personalized sequencing assay. 2020. 735 S.
58. Marsico G, Sharma G, Perry M, Hackinger S, Forshew T, Howarth K, u. a. Abstract 3097: Analytical development of the RaDaR assay, a highly sensitive and specific assay for the monitoring of minimal residual disease. Cancer Res. 15. August 2020;80(16 Supplement):3097.
59. Leaflet for: CELLSEARCH® Circulating Tumor Cell Epithelial Kit by Menarini Silicon Biosystems, Inc,.
60. Aaronson NK, Ahmedzai S, Bergman B, Bullinger M, Cull A, Duez NJ, u. a. The European Organization for Research and Treatment of Cancer QLQ-C30: a quality-of-life instrument for use in international clinical trials in oncology. J Natl Cancer Inst. 3. März 1993;85(5):365–76.

61. Mehnert A, Herschbach P, Berg P, Henrich G, Koch U. Progredienzangst bei Brustkrebspatientinnen - Validierung der Kurzform des Progredienzangstfragebogens PA-F-KF/ Fear of progression in breast cancer patients – validation of the short form of the Fear of Progression Questionnaire (FoP-Q-SF). Zeitschrift für Psychosomatische Medizin und Psychotherapie. 1. Juli 2006;52(3):274–88.

62. World Medical Association, DECLARATION OF HELSINKI – ETHICAL PRINCIPLES FOR MEDICAL RESEARCH INVOLVING HUMAN SUBJECTS; https://www.wma.net/policies-post/wma-declaration-of-helsinki-ethical-principles-for-medical-research-involving-human-subjects, visited 20.04.2022.
